# A Fluorescent Probe Enables the Discovery of Improved Antagonists Targeting the Intracellular Allosteric Site of the Chemokine Receptor CCR7

**DOI:** 10.1021/acs.jmedchem.4c02102

**Published:** 2025-02-12

**Authors:** Silas L. Wurnig, Max E. Huber, Corinna Weiler, Hanna Baltrukevich, Nicole Merten, Isabel Stötzel, Teresa Steffen, Yinshui Chang, René H. L. Klammer, Dirk Baumjohann, Eva Kiermaier, Peter Kolb, Evi Kostenis, Matthias Schiedel, Finn K. Hansen

**Affiliations:** † Department of Pharmaceutical & Cell Biological Chemistry, Pharmaceutical Institute, University of Bonn, An der Immenburg 4, Bonn 53121, Germany; ‡ Department of Chemistry and Pharmacy, Medicinal Chemistry, Friedrich-Alexander-University Erlangen-Nürnberg, Nikolaus-Fiebiger-Straße 10, Erlangen 91058, Germany; § Molecular, Cellular and Pharmacobiology Section, Institute for Pharmaceutical Biology, University of Bonn, Nussallee 6, Bonn 53115, Germany; ∥ Department of Pharmaceutical Chemistry, University of Marburg, Marbacher Weg 8, Marburg 35037, Germany; ⊥ Life and Medical Sciences (LIMES) Institute, Immune and Tumor Biology, University of Bonn, Bonn 53115, Germany; ¶ Medical Clinic III for Oncology, Hematology, Immuno-Oncology and Rheumatology, University Hospital Bonn, University of Bonn, Venusberg-Campus 1, Bonn 53127, Germany; ∇ Institute of Medicinal and Pharmaceutical Chemistry, Technische Universität Braunschweig, Beethovenstraße 55, Braunschweig 38106, Germany

## Abstract

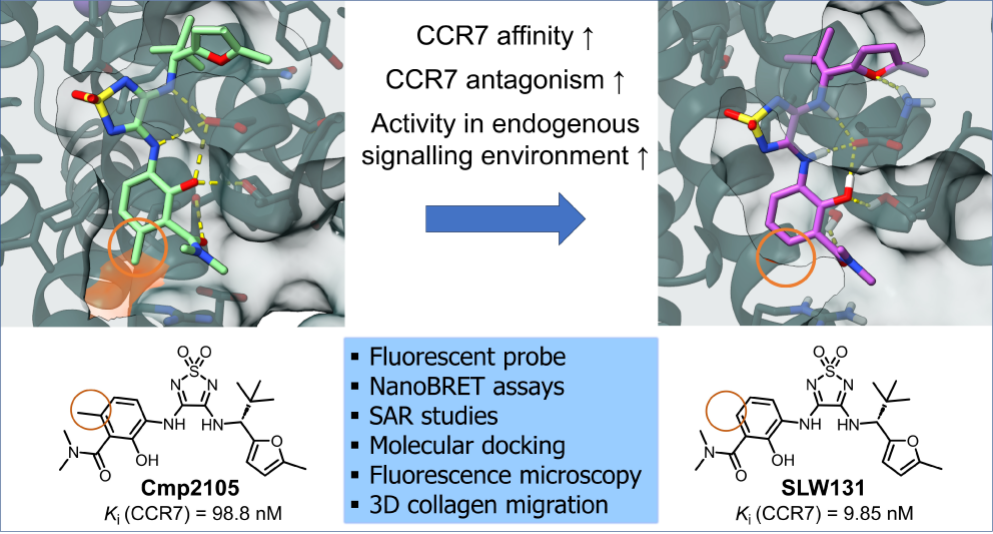

Intracellular ligands of G protein-coupled receptors (GPCRs) are gaining significant interest in drug discovery. Here, we report the development of the fluorescent ligand Mz437 (**4**) targeting the CC chemokine receptor CCR7 at an intracellular allosteric site. We demonstrate its experimental power by applying **4** to identify two improved intracellular CCR7 antagonists, SLW131 (**10**) and SLW132 (**21m**), developed by converting two weakly active antagonists into single- or double-digit nanomolar ligands with minimal modifications. The thiadiazoledioxide **10** was derived from the CCR7 antagonist Cmp2105 by removing a methyl group from the benzamide moiety, while the squaramide **21m** was obtained from the CXCR1/CXCR2 antagonist and clinical candidate navarixin by replacing the ethyl substituent by a *tert*-butyl group to engage a lipophilic subpocket. We show that **10** and **21m** qualify to probe CCR7 biology in recombinant and primary immune cells and expect our novel probes to facilitate the design of next-generation intracellular CCR7 ligands.

## Introduction

**Figure 1:**
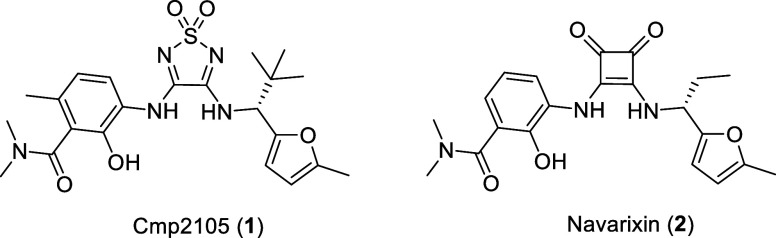
Chemical structures of the thiadiazoledioxide-based intracellular CCR7 antagonist Cmp2105 (**1**) and the squaramide-based CXCR1/CXCR2 antagonist navarixin (**2**).

G protein-coupled receptors (GPCRs) are of high importance for drug development, as they represent more than 30% of the targets of currently used medications.^1,2^ Historically, the majority of GPCR ligands have targeted the orthosteric site, which is located within a conserved helical bundle accessible from the extracellular side.^1^ In addition to the orthosteric site, a conserved intracellular allosteric binding site (IABS) has recently been identified for several GPCRs. So far, the presence of an IABS has been verified through X-ray cocrystallography for the chemokine receptors CXCR2, CCR2, CCR7, and CCR9, as well as the beta-2-adrenergic receptor (β_2_-AR).^3−9^ Furthermore, the existence of a druggable IABS has been proposed for other GPCRs (e.g., CCR1, CCR6, CXCR1).^10,11^ The allosteric nature of these intracellularly binding ligands enables a novel dual-mechanism of action approach to GPCR modulation. This mode of action involves (i) the stabilization of the inactive conformation of the receptor, thereby leading to negative cooperativity with the orthosteric agonist, and (ii) the resulting steric hindrance blocks the binding of intracellular transducers such as G proteins and regulatory molecules like G protein-coupled receptor kinases and β-arrestins, respectively. By blocking receptor:effector interactions directly at their interface, intracellular GPCR antagonists may effectively lock receptors in their inactive state without the need to directly compete with the activating ligand. Notably, most of the receptors identified with an IABS are chemokine receptors, which are implicated in several cancers associated with poor prognosis but also in nononcological conditions such as immune cell trafficking and homing as well as inflammatory processes.^12−15^ Thus, targeting the IABS in chemokine receptors represents a new avenue for therapeutic interventions in a variety of diseases. The CC chemokine receptor type 7 (CCR7) is a receptor specifically interacting with the C–C motif chemokines CCL19 and CCL21.^16^ Its primary expression occurs in diverse lymphoid tissues.^16,17^ CCR7 plays a vital role in lymphocyte and dendritic cell (DC) homing to secondary lymphoid organs and has been recognized as a potential therapeutic target in diseases such as rheumatoid arthritis, pathogenic infections and lymph node metastasis.^17^ Consequently, there is a clinical demand for the discovery and characterization of selective modulators capable of regulating CCR7 downstream signaling pathways. The absence of known orthosteric antagonists for CCR7 has posed challenges in the development of drugs targeting this receptor to date. However, a recent breakthrough by Jaeger et al. resolved the crystal structure of Cmp2105 (**1**, Figure 1) bound to the IABS of CCR7 through X-ray crystallography.^5^ They discovered that Cmp2105 (**1**) binds to the IABS similarly to vercirnon^1,18^ in CCR9 and Cmpd-15PA in β_2_-AR.^7,19^ This, together with the observed inhibitory effect on CCL19 binding, identifies Cmp2105 (**1**) as an intracellular allosteric antagonist that may serve as a promising lead compound for the development of improved CCR7 antagonists. Additionally, Jaeger et al. screened known antagonists of other chemokine receptors and discovered, unexpectedly, that the established CXCR1/CXCR2 antagonist navarixin (**2**, Figure 1) also binds to CCR7. Docking studies suggested that navarixin (**2**) interacts with the IABS of CCR7 in a similar fashion as Cmp2105.^5^ Given their nearly identical substituents attached to the thiadiazoledioxide or squaramide core, these two compounds hold significant promise as lead structures for the development of novel intracellular CCR7 antagonists.

To date, the characterization of CCR7 ligands targeting the IABS has focused on thermofluor stability assays and functional assays (e.g., cellular arrestin recruitment assays).^5,20^ Although thermofluor stability assays are a valuable tool for initial screening of protein–ligand interactions, they have several limitations. These limitations include: (i) applicability only to proteins that denature with heat, (ii) significant dependence on the specific fluorescent dye used, (iii) a substantial likelihood of misinterpretation, and (iv) a rather qualitative or at best semiquantitative nature, making them unsuitable for accurate determination of binding affinities.

We recently developed fluorescent tracers targeting the IABS of CCR2 and CCR9.^21,22^ These tools have been effectively utilized in cell-free and cellular binding studies using the NanoBRET technology.^21,22^ In the present study, we report the development of a fluorescent thiadiazoledioxide-based intracellular CCR7 ligand derived from Cmp2105 (**1**). This molecular tool enabled to probe the binding of established and novel ligands to the IABS of CCR7 both in a cell-free and cellular environment. The most promising and selective CCR7 ligands identified in this screening were further characterized in a panel of functional assays in both recombinant and primary cells. This workflow allowed us to discover intracellular CCR7 antagonists with improved affinity and enhanced antagonistic behavior compared to Cmp2105 (**1**), qualified as probes to investigate contribution of CCR7 to complex biological responses also in the endogenous signaling environment.

## Results and Discussion

### Design and Synthesis of a Fluorescent Probe Targeting the IABS of CCR7

**Figure 2:**
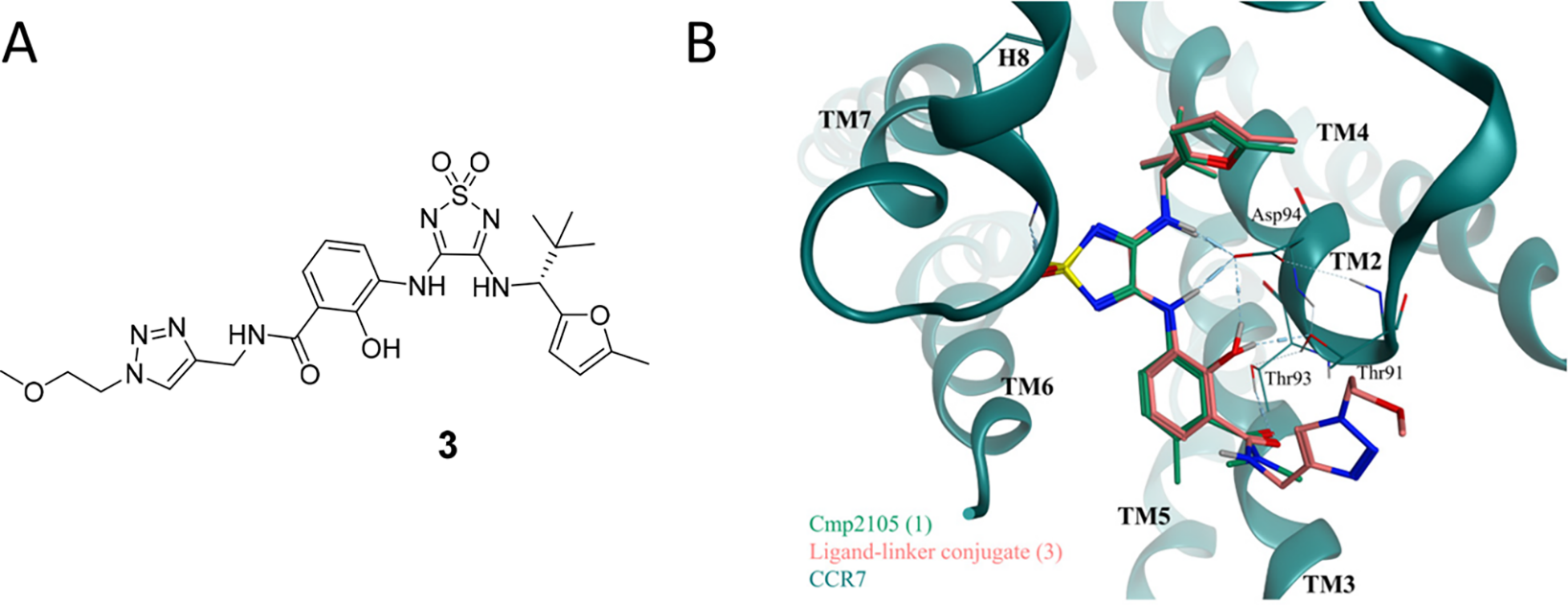
Design of a fluorescent ligand targeting the intracellular allosteric binding site of CCR7. (A) Chemical structure of the triazole-based CCR7 ligand-linker conjugate **3** that was designed by introducing a linker which had already been successfully implemented in ligands targeting the IABS of other chemokine receptors. (B) Overlay of the reported binding mode of Cmp2105 (**1**, green; PDB ID: 6QZH) with the predicted binding mode of the Cmp2105-derived ligand-linker conjugate **3** (salmon) in the IABS of CCR7 (green).

The cocrystal structure of human CCR7 in complex with the intracellular allosteric antagonist Cmp2105 (**1**) (PDB ID: 6QZH) provided the foundation for the structure-based design of a fluorescent tracer for the IABS of CCR7.^5^ The examination of this structure indicated that the *N*,*N*-dimethylamide moiety of Cmp2105 (**1**) is sufficiently solvent exposed to facilitate the attachment of a fluorophore for the development of a fluorescently labeled intracellular CCR7 ligand. To simplify the synthesis and since both moieties are not involved in crucial ligand–receptor interactions in the CCR7-Cmp2105 (**1**) cocrystal structure,^5,20^ we omitted the methyl groups adjacent to the *N*,*N*-dimethylamide group and in position 6 of the benzamide. Our prior research on TAMRA-labeled ligands for other chemokine receptors, including CCR1, CCR2, CCR6, CCR9, CXCR1 and CXCR2, has shown that such ligands are effective molecular tools to investigate the binding of ligands to the IABS of GPCRs in both cell-free and, more importantly, cellular setups.^10,11,21−23^ Based on this experience, we aimed to attach a TAMRA fluorophore using a Cu(I)-catalyzed Huisgen cycloaddition to a Cmp2105 analog. To visualize the correctness of the aforementioned assumptions, we conducted molecular docking studies with the triazole-based CCR7 ligand-linker conjugate **3***post hoc,* indicating a highly similar binding mode as observed for Cmp2105 (**1**) in the cocrystal structure with CCR7 (Figure 2).

**Scheme 1:**
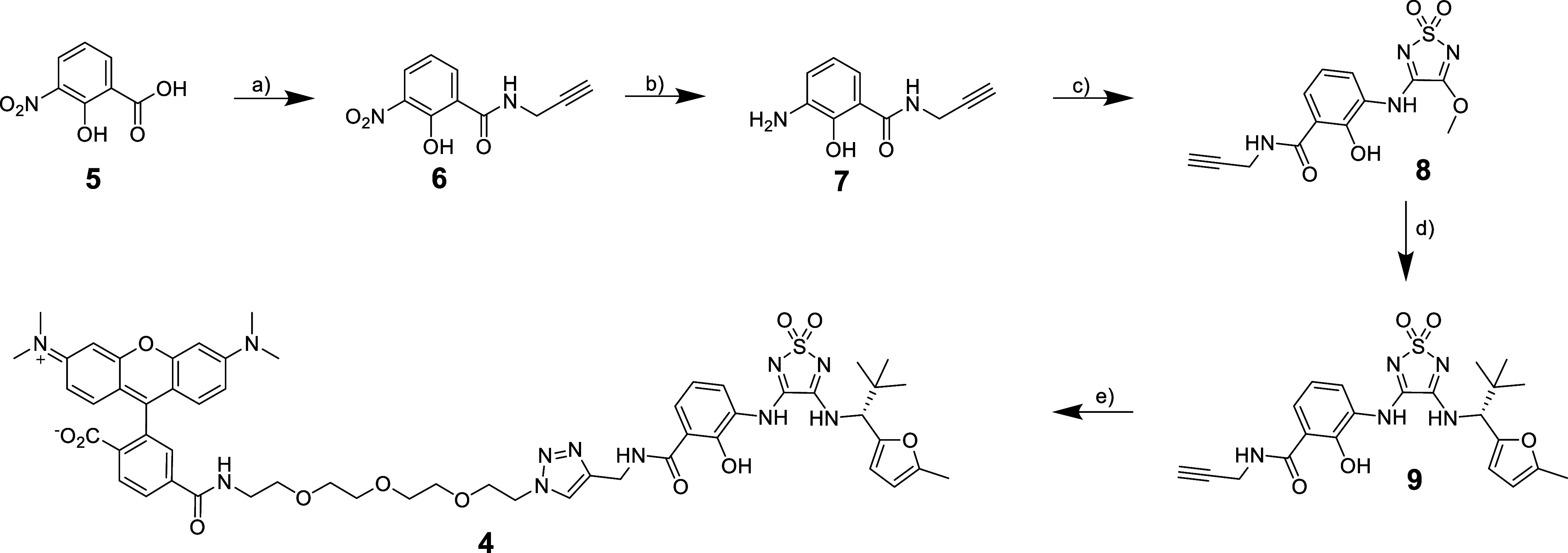
Synthesis of the Fluorescent Tracer **4** Reagents and conditions: (a) Propargylamine, PyBroP, DIPEA, CH_2_Cl_2_, rt, overnight, 79% yield. (b) SnCl_2_·H_2_O, MeOH, reflux, 3 h, quantitative yield. (c) 3,4-Dimethoxy-1,2,5-thiadiazole-1,1-dioxide, MeOH, rt, 3 days, quantitative yield. (d) (*R*)-2,2-Dimethyl-1-(5-methylfuran-2-yl)propan-1-amine hydrochloride, DIPEA, MeOH, rt, 7 days, 66% yield. (e) 6-TAMRA-PEG_3_-azide, CuSO_4_·5H_2_O, sodium ascorbate, TBTA, water/*tert*-BuOH/DMF mixture (1:1:1 (*v/v/v*)), rt, 6 h, 52% yield.

The synthesis of the designed fluorescent CCR7 probe **4** is summarized in Scheme 1. First, 3-nitrosalicylic acid (**5**) was converted into the corresponding propargyl amide **6** by an amide bond formation using PyBroP and DIPEA as coupling system to obtain the desired product. The reduction of the nitro group using tin(II) chloride monohydrate afforded the corresponding aniline **7**. In the next step, **7** was reacted with the readily available building block 3,4-dimethoxy-1,2,5-thiadiazole-1,1-dioxide to obtain the monosubstituted 1,2,5-thiadiazole-1,1-dioxide **8**. The subsequent second substitution reaction of intermediate **8** with (*R*)-2,2-dimethyl-1-(5-methylfuran-2-yl)propan-1-amine provided the clickable CCR7 ligand **9**. This ligand was then subjected to a Cu(I)-catalyzed azide–alkyne cycloaddition (CuAAC) with an azido-functionalized TAMRA analog to furnish the desired fluorescent tracer **4**.

### NanoBRET Assay Development for CCR7

**Figure 3:**
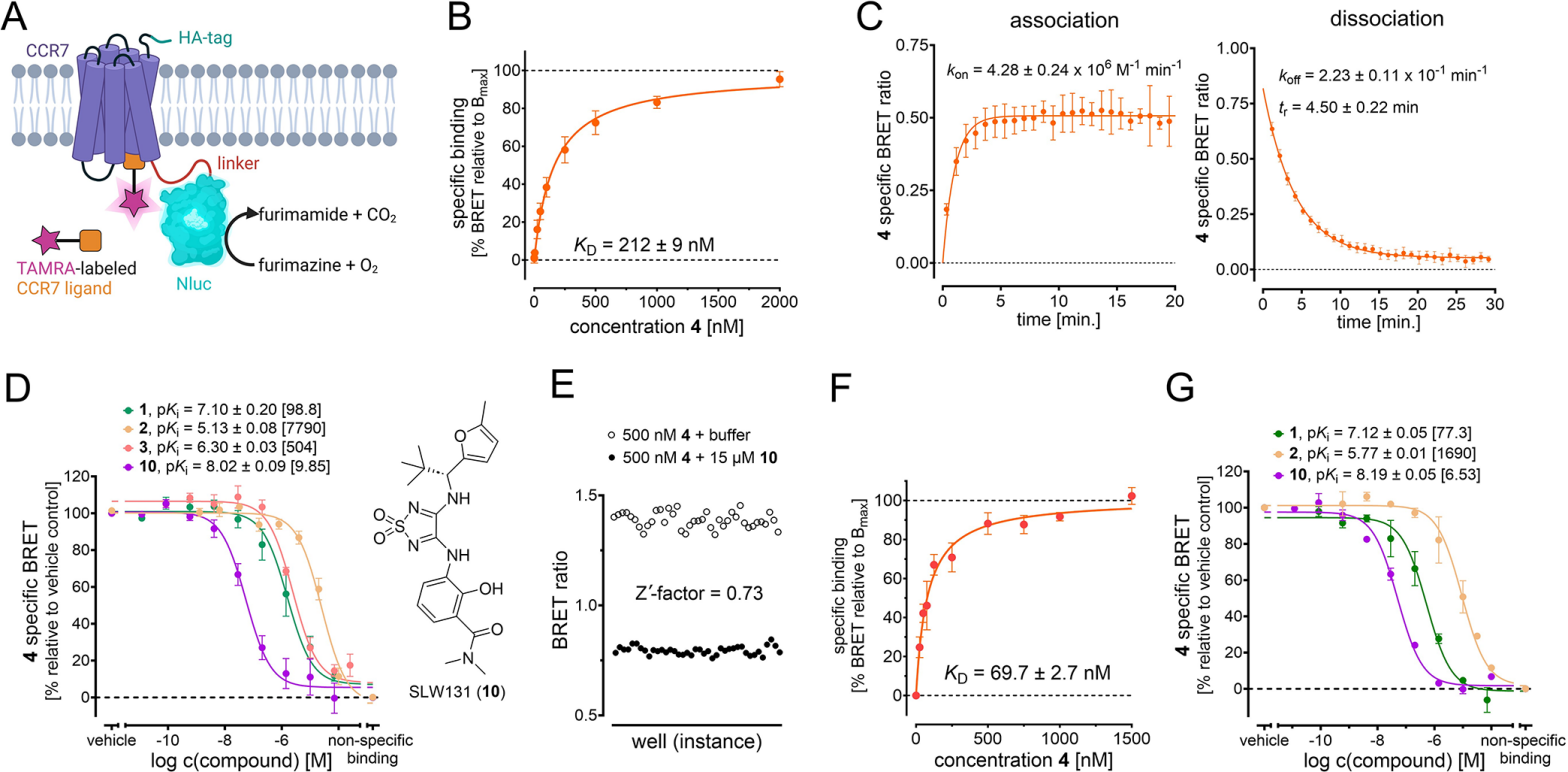
Application of Mz437 (**4**) as a fluorescent tool for cell-free and cellular NanoBRET-based binding studies targeting the IABS of CCR7. (A) Cartoon representation of the NanoBRET strategy to detect binding to the IABS of CCR7. (B) Specific saturation binding curve of the fluorescent tracer **4** in a NanoBRET-based assay using CCR7_GSSG_Nluc membranes (mean ± SEM, triplicate measurement, *n* = 3). (C) Kinetic binding studies at room temperature. Representative association and dissociation curves with **4** (250 nM) using CCR7_GSSG_Nluc membranes (duplicate measurement). (D) Competition binding curves and detected p*K*_i_ values (mean ± SEM, triplicate measurement, *n* ≥ 3) for the intracellular CCR7 antagonist Cmp2105 (**1**),^5^ the CXCR1/CXCR2 antagonist navarixin (**2**) with reported off-target binding to CCR7,^5^ the linker-ligand conjugate SLW151 (**3**), and **10** (chemical structure on the right), which is the desmethyl analogue of Cmp2105 (**1**). Data was obtained with **4** (500 nM) and CCR7_GSSG_Nluc membranes. *K*_i_ values [nM] are given in square brackets. (E) Z’-factor determination for the cell-free NanoBRET-based setup using Mz437 (**4**, 500 nM) and CCR7_GSSG_Nluc membranes (36-fold determination). (F) Saturation binding curve of Mz437 (**4**) in a cellular NanoBRET-based experiment (mean ± SEM, quadruplicate measurement, *n* = 4) using live HEK293T cells transiently expressing CCR7_GSSG_Nluc. (G) Cellular competition binding curve and p*K*_i_ values (mean ± SEM, quadruplicate measurement, *n* ≥ 3) for Cmp2105 (**1**), navarixin (**2**), and **10** obtained with Mz437 (**4**, 500 nM) and live HEK293T cells transiently expressing CCR7_GSSG_Nluc. *K*_i_ values [nM] are given in square brackets.

To evaluate the capacity of Mz437 (**4**) to serve as a molecular tool for studying ligand binding to the IABS of CCR7, we established a NanoBRET-based binding assay (Figures 3A–G and S1A–G). To this end, we fused the small bioluminescent Nanoluciferase (Nluc)^24^ either directly to the intracellular C terminus of CCR7 or with a GSSG linker between CCR7 and the Nluc-tag (Figures 3A and S1A), in a similar manner as reported for CCR2, CCR6, or CCR9.^10,19,21^ Successful surface expression of the CCR7-Nluc fusion proteins, hereafter referred to as CCR7_Nluc and CCR7_GSSG_Nluc, was confirmed by ELISA (Figure S1B). In first cell-free experiments with membranes from HEK293T cells transiently expressing CCR7_Nluc and CCR7_GSSG_Nluc, respectively, we observed a slightly larger assay window for CCR7_GSSG_Nluc (Figure S1E), and thus used this construct for further investigations. In cell-free saturation binding experiments using CCR7_GSSG_Nluc membranes, Mz437 (**4**) was characterized as a high affinity intracellular CCR7 ligand with an equilibrium dissociation constant (*K*_D(eq )_) of 212 nM (Figures 3B and S1F). Membrane-based kinetic binding experiments indicated both fast association (*k*_on_ = 4.28 × 10^6^ M^–1^ min^–1^) and dissociation (*k*_off_ = 2.23 × 10^–1^ min^–1^, *t*_r_ = 4.50 min) of the receptor–ligand complex (Figure 3C and Table S1). The derived kinetic dissociation constant (*K*_D(kin.)_) of 52.2 nM is in a similar range as the detected equilibrium *K*_D_ (*vide supra*). Then, we applied Mz437 (**4**) in a cell-free competition binding setup, to report the binding of nonfluorescent ligands to the IABS of CCR7 (Figure 3D). Using this setup, we detected a two-digit nanomolar affinity for the thiadiazoledioxide Cmp2105 (**1**, *K*_i_ = 98.8 nM). The approximately 3-fold difference between the reported IC_50_ value of 35 nM,^5^ which was determined by means of a membrane-based competition experiment with the radioactively labeled orthosteric agonist CCL19, and the *K*_i_ value from our NanoBRET-based competition binding assay can be rationalized by the strongly different concepts and setups of the applied GPCR binding assays. A much weaker affinity was detected for the squaramide navarixin (**2**, *K*_i_ = 7790 nM), which is in line with the results from dose response thermal shift assays reported by Jaeger et al.^5^ For the thiadiazoledioxide-based linker-ligand conjugate **3** (see Scheme S1 for synthetic details) that shares the same CCR7-binding unit as our fluorescent tracer Mz437 (**4**), we determined a *K*_i_ value of 504 nM. This underlines that all changes at the scaffold of Cmp2105 (**1**) that were done in the course of the design of our fluorescent ligand (i.e., attachment of a linker at the benzamide N atom, switch from a tertiary to a secondary benzamide, removal of the methyl group in position 6 of the benzamide) were well tolerated. To probe the effect of the removal of the methyl group in position 6 of the benzamide, we synthesized and tested the respective desmethyl analogue **10** (Figure 3D, see experimental section for synthetic details) of Cmp2105 (**1**). Intriguingly, we observed an approximately 10-fold increased CCR7 affinity for **10** (*K*_i_ = 9.85 nM) compared to the parent compound Cmp2105 (**1**). Initial selectivity profiling, using previously reported NanoBRET assays to detect intracellular binding to the closely related chemokine receptors CCR2 and CCR9, indicated that **10** displays an improved CCR7 selectivity compared to Cmp2105 (Table S2). In a next step, we assessed the suitability of our cell-free NanoBRET competition binding assay for high-throughput screening (HTS) approaches via a Z’-factor determination according to Zhang et *al.*(25) These investigations resulted in a Z’-factor of 0.73 (Figure 3E), thus indicating that our cell-free CCR7 binding assay can be considered as excellent and suitable for HTS approaches.

After having shown that Mz437 (**4**) is a highly valuable tool for studying ligand binding to the IABS of CCR7 in a high-throughput manner under cell-free conditions, we aimed for transferring our NanoBRET-based assay protocol to a live cell environment. Methods to directly monitor ligand binding to a specific target or target site in a cellular environment, also referred to as cellular target engagement, are highly important in early drug discovery. This is especially important for intracellular target proteins or transmembrane proteins with an intracellular binding site, such as the IABS of GPCRs. To the best of our knowledge, no small-molecule tracers have been reported thus far that enabled cellular binding assays for the IABS of CCR7. In a cellular saturation binding assay setup, using live HEK293T cells overexpressing CCR7_GSSG_Nluc, we detected a *K*_D_ value of 69.7 nM for our fluorescent CCR7 tracer Mz437 (**4**, Figure 3F). With this, we demonstrated that Mz437 (**4**) is able to pass the cell membrane of live cells and bind to the IABS of CCR7 in a live cell environment. Using Mz437 (**4**) as a tracer for cell-based competition binding assays, we detected a two-digit nanomolar affinity for Cmp2105 (**1**, *K*_i_ = 77.3 nM), a strongly reduced affinity for navarixin (**2**, *K*_i_ = 1690 nM), and a single-digit nanomolar affinity for **10** (*K*_i_ = 6.53 nM, Figure 3G). These results are (i) highly consistent with the affinity data from our cell-free experiments (Figure 3D); (ii) clearly showing the suitability of Mz437 (**4**) as a tracer to study cellular target engagement for the IABS of CCR7, and; (iii) highlighting **10** as an intracellular CCR7 ligand with significantly improved affinity in a cell-free and cellular environment, as compared to the prior gold standard Cmp2105 (**1**).

### Docking Studies of Antagonist 10 vs. Cmp2105 (**1**)

**Figure 4:**
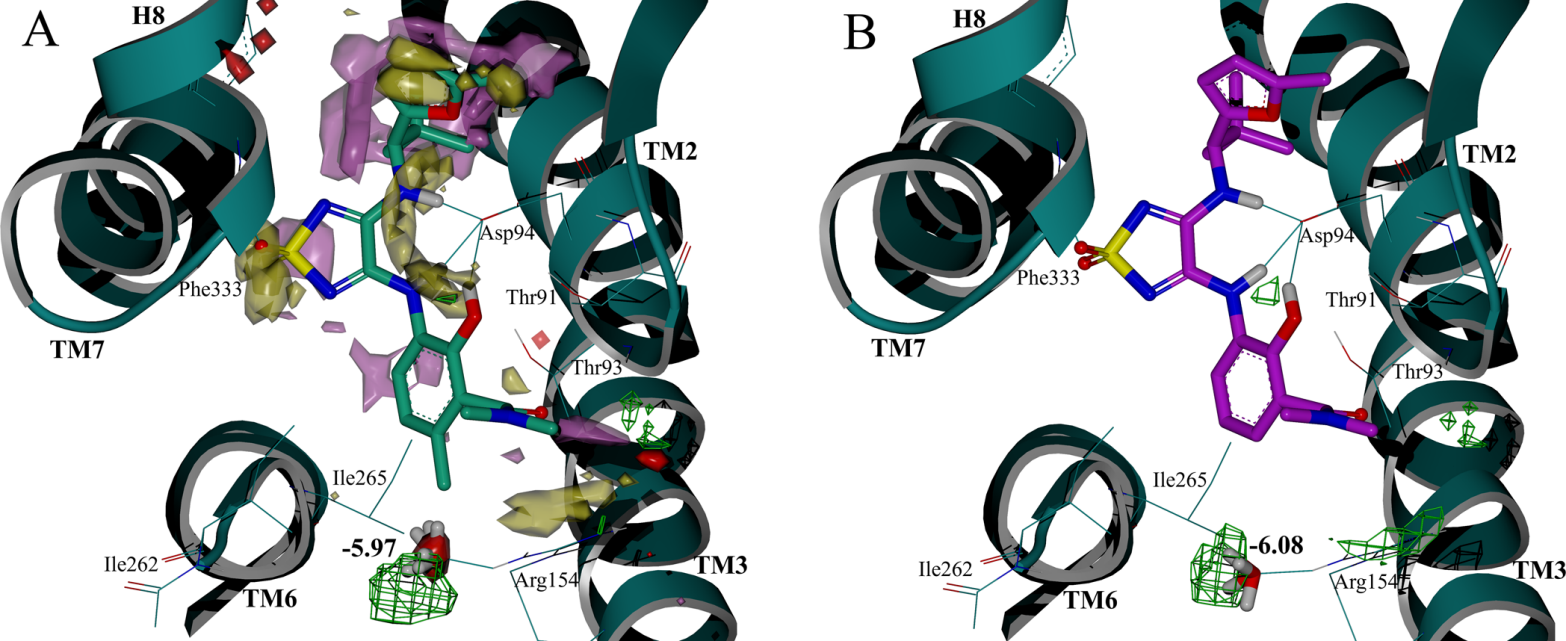
(A) SZMAP grid calculations in the CCR7 IABS (PDB ID: 6QZH), shown as green ribbon: ligand displacement water map of Cmp2105 (**1**, green carbons). Residues within 4.5 Å from the methyl group of interest are shown as wires. Solid surfaces correspond to the water regions having negative (yellow) or positive (purple) neutral difference free energies. Water molecules are expected to enhance binding affinity when located in negative regions and decrease it when located in positive regions. Calculated difference between the Cmp2105 (**1**) and **10** grid values is highlighted as red solid surface. Region where water stabilizes the protein–ligand complex is shown in green mesh. Sampled water molecule orientations are shown as sticks. This water molecule is hypothesized to be displaced by the methyl group of Cmp2105 (**1**), but not by **10**, and has a predicted energy value of −5.97 kcal/mol. (B) SZMAP grid calculations in the CCR7 IABS (PDB ID: 6QZH), shown as green ribbon: protein–ligand complex water map of **10** (magenta carbons). The water molecule corresponding to the one displaced by Cmp2105 (**1**) is shown as sticks. Free energy value of −6.08 kcal/mol is similar to the energy value of the displaced water molecule in the Cmp2105 (**1**) complex (−5.97 kcal/mol). Stabilization grid is shown in green mesh.

Cell-based and cell-free competition binding assays showed an unexpected 10-fold affinity improvement of the desmethylated derivative **10** compared to the parent compound Cmp2105 (**1**). Intrigued by such a shift in the binding affinity, we performed molecular docking calculations to rationalize the effect of this single methyl group removal. Not surprisingly, we obtained almost identical binding modes for **10** and Cmp2105 (**1**). Taking a closer look at the crystal structure of Cmp2105 in CCR7’s IABS (PDB ID: 6QZH), we noted that the methyl group of interest is solvent-exposed. We therefore hypothesized that the binding affinity was affected by the different interactions between the ligands and the solvent in that region. To test this hypothesis, we ran SZMAP calculations to predict whether any water molecules were potentially influenced by the introduction of the methyl group. The SZMAP calculations with Cmp2105 (**1**) and the desmethyl analogue **10** showed that the main difference between the two predicted water maps lies in the region highlighted as a red solid surface in Figure 4A. Exactly in that location, we observed a region in the displacement grid where the methyl group of Cmp2105 displaces an energetically favorable water molecule (predicted SZMAP free energy value −5.97 kcal/mol) from the *apo* grid (Figure 4A; sampled orientations of the water molecule shown as sticks). This is corroborated by stabilization calculations (depicted in green mesh in Figure 4A), which indicated that this water molecule has a stabilizing effect on the protein–ligand complex. Displacement of such a stabilizing water molecule could in theory be counterbalanced by newly emerging energetically favorable interactions made by functional groups of a ligand—yet not via a methyl group, as in Cmp2105 (**1**). Consistent with this assumption, in the protein–ligand complex of CCR7 and **10**, the corresponding energetically favorable water molecule at the equivalent position is not displaced by the ligand and has essentially the same calculated free energy value of −6.08 kcal/mol (Figure 4B). Therefore, we hypothesize that the methyl group of Cmp2105 (**1**) displaces an energetically favorable water molecule, which – when present – stabilizes the protein–ligand complex. Since the methyl group of Cmp2105 (**1**) cannot compensate for the lost interactions, this would lead to a net loss in binding free energy. When the desmethylated ligand **10** is bound, a water molecule at this position would be left unperturbed, an explanation consistent with the experimentally observed increased potency.

### SAR Study for Thiadiazoledioxide- and Squaramide-Based Intracellular CCR7 Antagonists

**Scheme 2:**
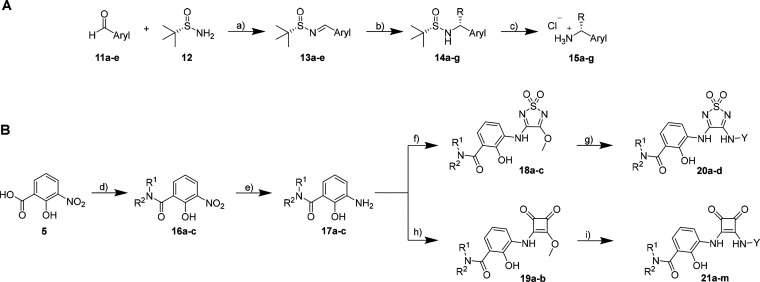
Synthesis of Novel Ligands for SAR Studies Reagents and conditions: (A) (a) Ti(OEt)_4_, DCM, rt, overnight, quantitative yield. (b) Respective alkylmagnesium chloride, THF, rt, 3 days, 5–54% yield. (c) HCl, Et_2_O, 0 °C, 1 h, quantitative yield. (B) (d) Respective amine, PyBroP, DIPEA, CH_2_Cl_2_, rt, overnight, 26–95% yield. (e) SnCl_2_·H_2_O, MeOH, reflux, 3 h, quantitative yield. (f) **17a**–**c**, 3,4-dimethoxy-1,2,5-thiadiazole-1,1-dioxide, MeOH, rt, 3 days, quantitative yield. (g) Respective amine hydrochloride, DIPEA, MeOH, rt, 7 days, 5–66% yield. (h) **17a**–**c**, dimethyl squarate, MeOH, rt, 3 days, quantitative yield. (i) Respective amine hydrochloride, DIPEA, MeOH, rt, 7 days, 5–66% yield.

To further expand the knowledge on the structure–activity relationships (SARs) of intracellular allosteric CCR7 antagonists, a series of novel Cmp2105 and navarixin analogues were synthesized as outlined in Scheme 2. The arylalkylamino part was synthesized in a similar fashion as previously published (Scheme 2A).^10,11^ Starting from the aldehydes **11a**–**e**, the imines **13a**–**e** were created by a condensation reaction with Ellman’s sulfinimide ((*S*)-2-methylpropane-2-sulfinamide, **12**)^26,27^ using titanium ethoxide as catalyst. The imines were then subjected to a Grignard type reaction with ethyl-, isopropyl- or *tert*-butyl magnesium chloride to obtain the products **14a**–**g**. This diastereoselective reaction produced exclusively the desired diastereomers (see Experimental Section for details), with the exception of the less sterically hindered ethyl derivative **14b**, which was obtained as a mixture of diastereomers. In the last step, the chiral auxiliary was removed by treatment of **14a**–**g** with hydrochloric acid to obtain the free amine hydrochlorides **15a**–**g**. The benzamide part was synthesized as summarized in Scheme 2B by the reaction of 3-nitrosalicylic acid (**5**) with the corresponding amines in a PyBroP-mediated amide coupling. The newly synthesized amides **16a**–**c** were then treated with tin(II)chloride monohydrate to obtain the free anilines **17a**–**c**. In the next step, the benzamide moiety was coupled to either 3,4-dimethoxy-1,2,5-thiadiazole-1,1-dioxide or dimethyl squarate to afford the intermediates **18a**–**c** and **19a**–**b**. Lastly, the synthesis of the desired Cmp2105 and navarixin analogues was finalized by the introduction of the second amine component by treatment of the corresponding thiadiazole-1,1-dioxide and squaramide intermediates with **15a**–**g** or commercially available amines to generate the final compounds **20a**–**d** and **21a**–**m**. The final compounds are summarized in Tables 1 and 2.

**Table 1:**
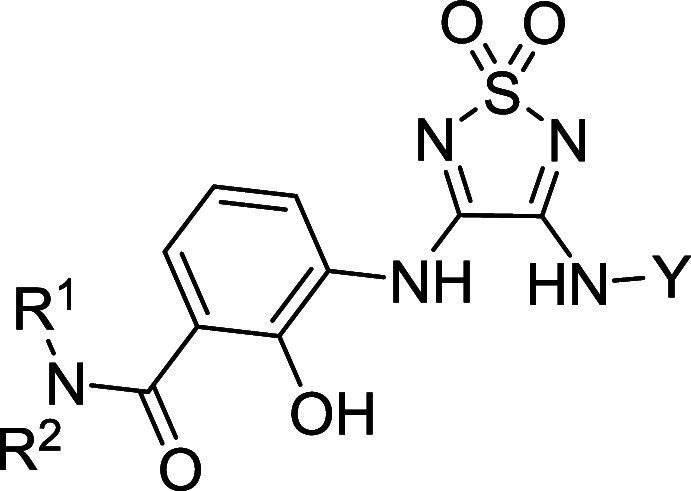

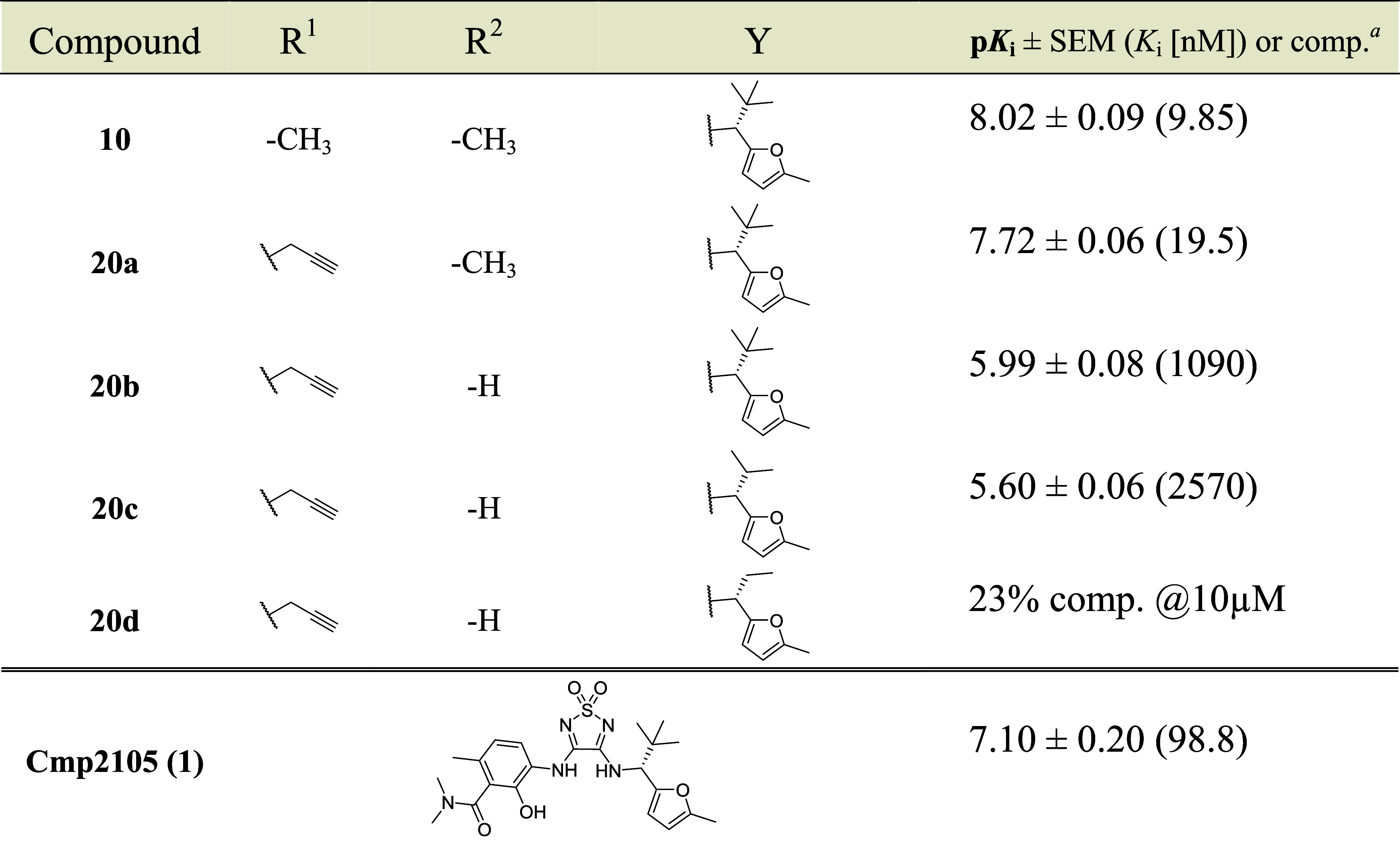
Overview of the Synthesized Thiadiazole-1,1-Dioxide-Based Cmp2105 Analogues and Their Respective p*K*_i_ Values (Mean ± SEM, Triplicate Measurement, *n* ≥ 3) Obtained from Our Cell-Free NanoBRET Competition Binding Assay^a^^b^

a Comp. = percentual competitive tracer displacement at given concentration.

b Data was obtained with **4** (500 nM) and CCR7_GSSG_Nluc membranes. *K*_i_ values are given in brackets.

**Table 2:**
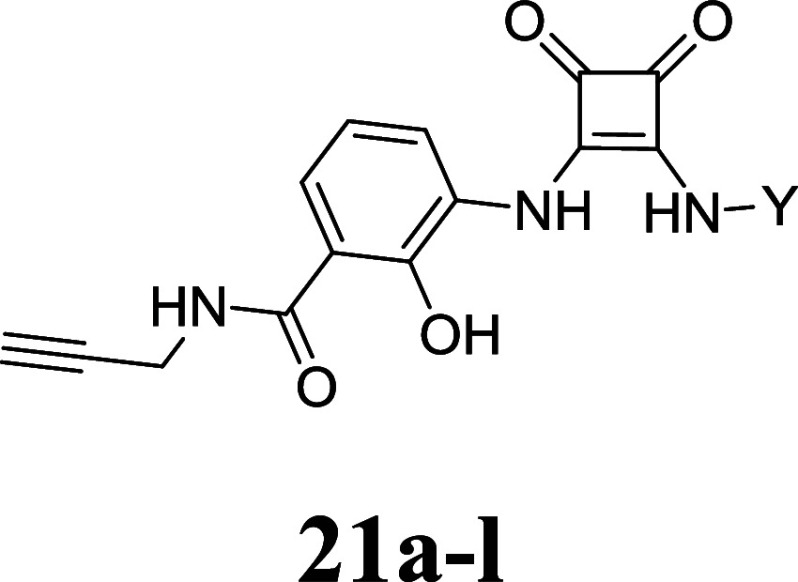

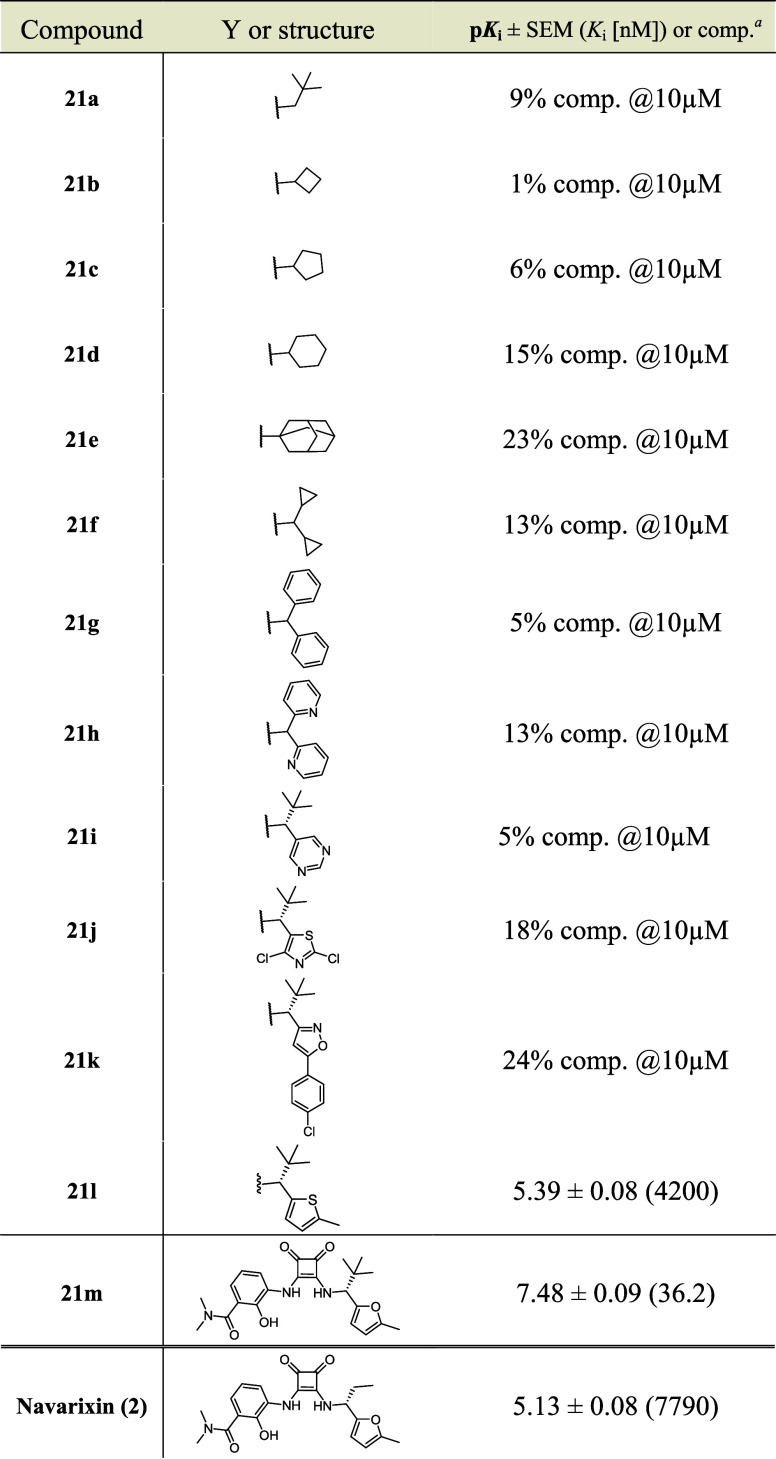
Overview of the Synthesized Squaramide-Based Navarixin Analogues and Their Respective p*K*_i_ Values (mean ± SEM, Triplicate Measurement, *n* ≥ 3) Obtained from Our Cell-Free NanoBRET Competition Binding Assay^a^^b^

a Comp. = percentual competitive tracer displacement at given concentration.

b Data was obtained with **4** (500 nM) and CCR7_GSSG_Nluc membranes. *K*_i_ values are given in brackets.

The screening of the thiadiazole-1,1-dioxide series in our cell-free NanoBRET-based CCR7 competition binding assay provided important SARs (Table 1). The replacement of one methyl group of the tertiary amide in **10** by a propargyl residue yielded compound **20a** with a *K*_i_ value of 19.5 nM. This modification was well tolerated, resulting only in a 2-fold reduced binding affinity compared to **10** (*K*_i_ = 9.85 nM) and, notably, a 5-fold improvement in affinity relative to Cmp2105 (**1**, *K*_i_ = 98.8 nM). As the propargyl group provides a valuable functional handle for the creation of bifunctional tools such as fluorescent ligands and targeted protein degraders, we chose to retain the propargyl residue at the R^1^ position throughout this SAR study. The subsequent conversion of the tertiary amide in **20a** to a secondary amide in **20b** (*K*_i_ = 1090 nM) led to a 56-fold decreased binding affinity. Interestingly, when the hydrophobicity and bulkiness of the *tert*-butyl moiety realized in **20b** was reduced to isopropyl (**20c**) and ethyl (**20d**), the affinities decreased in the order **20b** > **20c** > **20d**.

To further investigate the SARs of our intracellular allosteric CCR7 antagonists, we performed a scaffold hopping approach to the navarixin-derived squaramide-based compounds **21a**–**m**. This enabled a straightforward access to a systematic variation of the furanylalkylamino part. However, the replacement of the chiral furanylalkylamino residue by various aliphatic, aromatic, and heterocyclic aromatic amines (see **21a**–**k**, Table 2) resulted in a complete loss of affinity toward CCR7. The only exception was the thiophene derivative **21l** featuring a *tert*-butyl residue at the chiral carbon. This modification resulted in low, yet quantifiable affinity toward CCR7 (*K*_i_ = 4200 nM, Table 2). When we screened the direct squaramide analog of **10**, compound **21m**, we observed the highest CCR7 affinity (*K*_i_ = 36.2 nM, Table 2) in the squaramide series. Similar to **10**, we detected strongly reduced CCR2 and CCR9 affinities for **21m** (Table S2) and significantly improved cellular target engagement (*K*_i_ = 14.2 nM, Figure S2) as compared to Cmp2105 (**1**, *K*_i_ = 77.3 nM, Figure 3G). As a result, our SAR data for CCR7 underline the importance of a tertiary amide at the benzamide moiety and the *tert*-butyl moiety at the chiral carbon.

**Figure 5:**
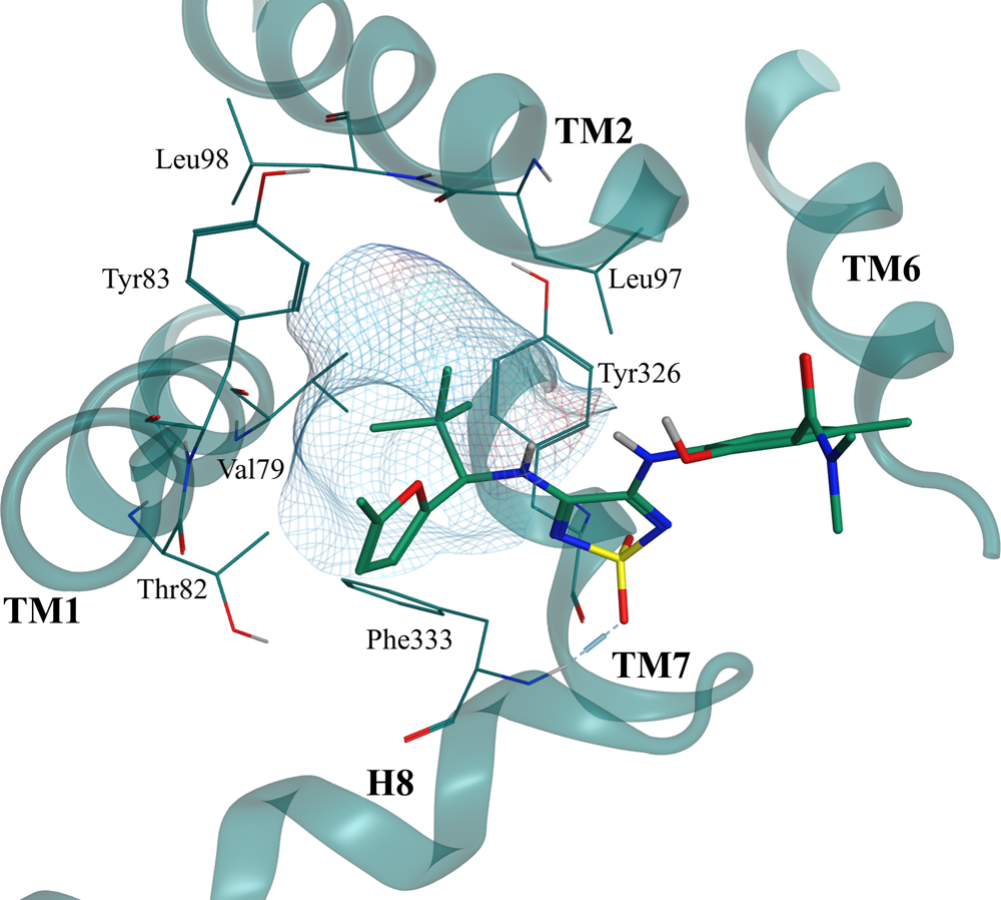
Visualization of the IABS of CCR7 (PDB ID: 6QZH) with a focus on the aromatic cage. Residues surrounding the *tert*-butyl group of Cmp2105 (**1**) are shown in lines. Molecular surface (green mesh) highlights the shape complementarity of the receptor’s subpocket and the *tert*-butyl substituent.

The critical role of the *tert*-butyl group is further confirmed by comparing the affinity data obtained for **21m** and navarixin (**2**). These compounds differ only in the residue at the chiral carbon (navarixin (**2**): (*R*)-ethyl; **21m**: (*R*)-*tert*-butyl). This modification led to a 215-fold decrease in binding affinity, which is in excellent agreement with the results from the thiadiazole-1,1-dioxide series (**20d**: (*R*)-ethyl; **10**: (*R*)-*tert*-butyl). In our previous study on squaramide-based intracellular antagonists targeting the IABS of CCR6 and CXCR1, we identified the *tert*-butyl moiety at the chiral carbon as an absolute sweet spot.^10^ Docking studies indicated that the *tert*-butyl group fits perfectly into an aromatic cage in the IABS of CCR6, thereby facilitating crucial hydrophobic interactions. We visualized aromatic residues within a distance of 4.5 Å around the *tert*-butyl group of Cmp2105 (**1**) in CCR7’s IABS (PDB ID: 6QZH) and identified a lipophilic subpocket analogous to CCR6’s IABS (Figure 5). This lipophilic subpocket is formed by Val79^1x53^, the methyl group of Thr82^1x56^, Tyr83^1x57^, Leu97^2x43^, Tyr326^7x53^ and Phe333^8x50^. All of these residues are identical in the IABS of CCR6, except for Tyr83^1x57^, which corresponds to Phe71^1x57^ in CCR6. Similarly, to the CCR6 IABS, we speculate that this aromatic cage in CCR7’s IABS allows for an optimal shape complementarity with the *tert*-butyl group, resulting in a favorable binding affinity. As a result, this could be an important contribution to the increased binding affinity of **21m** relative to navarixin (**2**). The optimal shape complementary of the IABS of CCR7 with the *tert*-butyl moiety was further highlighted by the very steep CCR7 SAR data for compounds **22a**–**i** (Table 3) that were previously reported as intracellular CCR6 and CXCR1 antagonists^10^ but have not yet been tested for their CCR7 affinity.

**Table 3:**
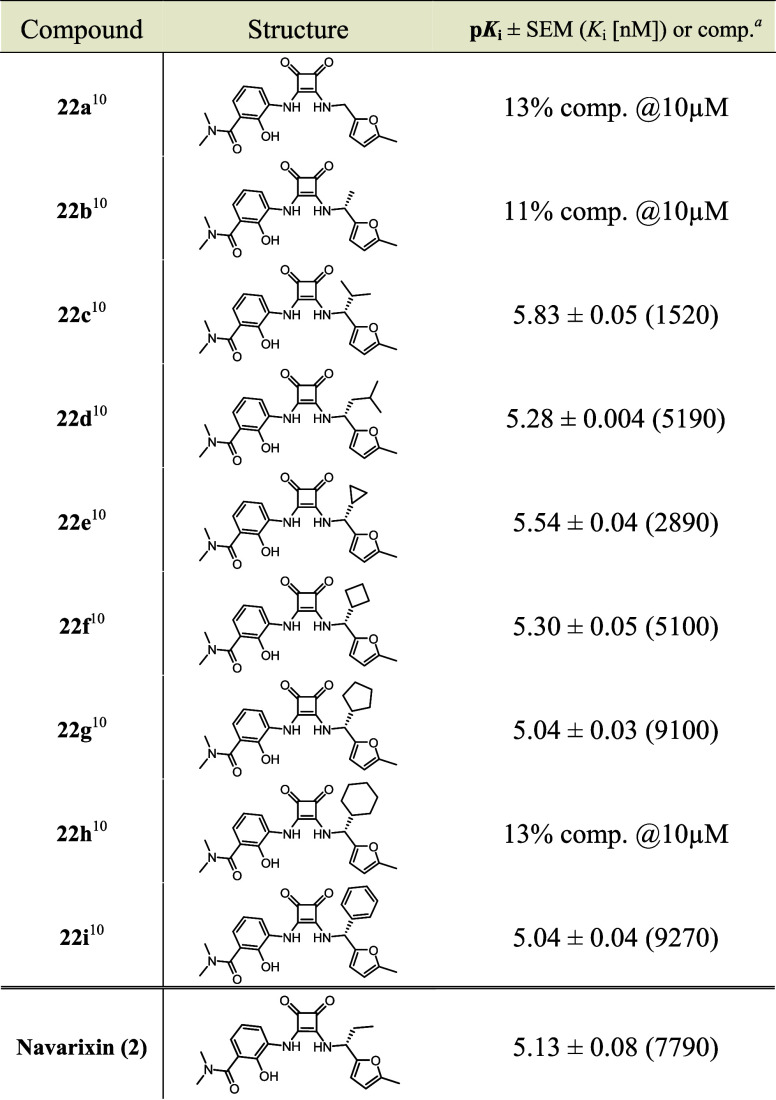
Structure-Activity Relationships of Additional Squaramide-Based Navarixin Analogues and Their Respective p*K*_i_ Values (Mean ± SEM, Triplicate measurement, *n* ≥ 3) Obtained From Our Cell-Free NanoBRET Competition Binding Assay^a^^b^

a Comp. = percentual competitive tracer displacement at given concentration.

b Data was obtained with **4** (500 nM) and CCR7_GSSG_Nluc membranes. *K*_i_ values are given in brackets.

In order to investigate if other pharmacophores designed to target the IABS of different GPCRs can also mediate CCR7 affinity, we tested a selection of known intracellular GPCR modulators for CCR7 binding using our cell-free competition binding assay (Table 4). With the exception of the CCR6-targeted squaramide PF-07054894,^28^ all of these subnanomolar to low-nanomolar intracellular GPCR modulators, including the CCR1/CCR2-targeted pyrrolone CCR2-RA,^9^ the CCR2-targeted biaryl sulfonamide SD-24,^29^ the CCR9-targeted biaryl sulfonamides vercirnon^30^ and AAA30,^22^ the CXCR2-targeted diaryl ureas danirixin^31^ and SB225002,^32^ and the NTSR1-targeted quinazoline SBI-553^33^ showed strongly reduced (>100-fold) binding to CCR7. On the one hand, these results further highlight the ability of CCR7’s IABS to bind squaramides, especially when these squaramides are decorated with substituents bearing bulky hydrophobic groups like 1-methylcyclopentyl as in the case of PF-07054894 or *tert*-butyl as in the case of **21m**. On the other hand, the low CCR7 affinities of all other investigated high affinity GPCR modulators (Table 4) together with the very steep SAR for both thiadiazole-1,1-dioxides (Table 1) and squaramides (Tables 2 and 3) indicate a very low promiscuity of the IABS of CCR7. Overall, from the SAR data for CCR7 presented in this manuscript and previously reported studies around the intracellular binding sites of CCR1,^1,23^ CCR2,^1,21,29^ CCR6,^10^ CCR9,^22,30^ CXCR1,^10,34^ and CXCR2,^10,11,34^ we can assign certain pharmacophores to distinct receptor groups. While pyrrolones were shown to bind intracellularly to CCR1 and CCR2,^1,23^ biaryl sulfonamides have a preference for the IABS of CCR2 and CCR9.^21,22,29,30,35^ The squaramide scaffold enables binding to CCR6,^10^ CCR7, CXCR1,^10,34^ and CXCR2.^10,11,34^ In the case of CCR6 and CCR7, an interaction of a bulky alkyl substituent (e.g., *tert*-butyl) with an aromatic cage seems to be essential to anchor the squaramide based ligand to its binding site, whereas CXCR1 and CXCR2 also tolerate smaller substituents (e.g., ethyl), as highlighted by the example of navarixin (**2**, *K*_i_(CXCR1) = 23.6 nM, *K*_i_(CXCR2) = 0.06 nM, *K*_i_(CCR6) = 1060 nM,^10^*K*_i_(CCR7) = 7790 nM). For the thiadiazole-1,1-dioxides, our data suggests a similar relevance of the interaction with the aromatic cage of CCR6 and CCR7, respectively, as observed for the squaramide series.

**Table 4:**
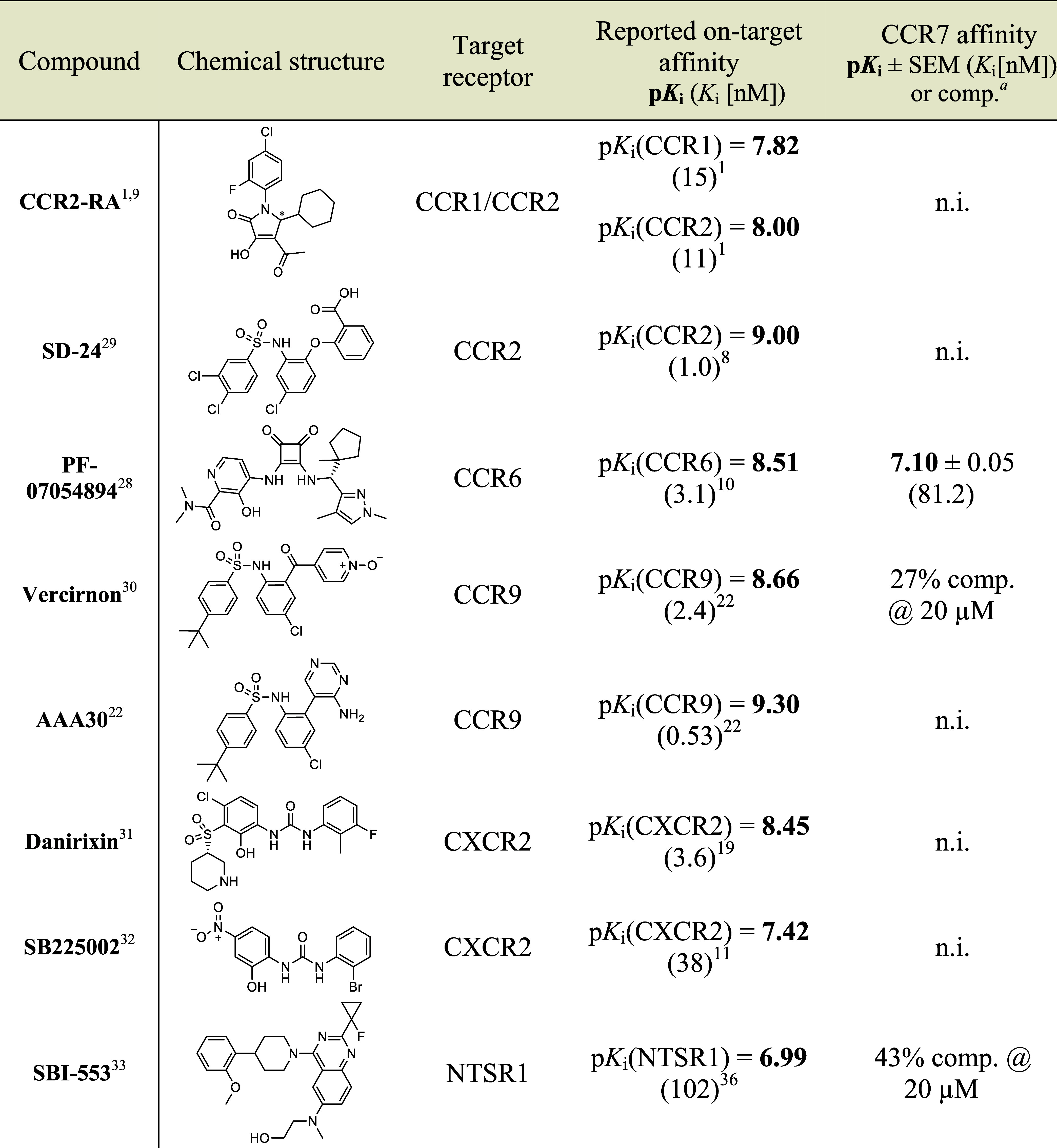
Binding Affinities of Known Intracellular GPCR Modulators for the IABS of CCR7^a^^b^

a Comp. = percentual competitive tracer displacement at given concentration.

b Data was obtained with **4** (500 nM) and CCR7_GSSG_Nluc membranes. Tests were performed at least in triplicate (*n* ≥ 3). Determined p*K*_i_ values for CCR7 are displayed as mean ± SEM and *K*_i_ values are given in brackets. n.i.: percentual competition at 20 μM ≤ 25%.

### Fluorescence Microscopy Visualizes IABS Targeting of Intracellular CCR7 Ligands

**Figure 6:**
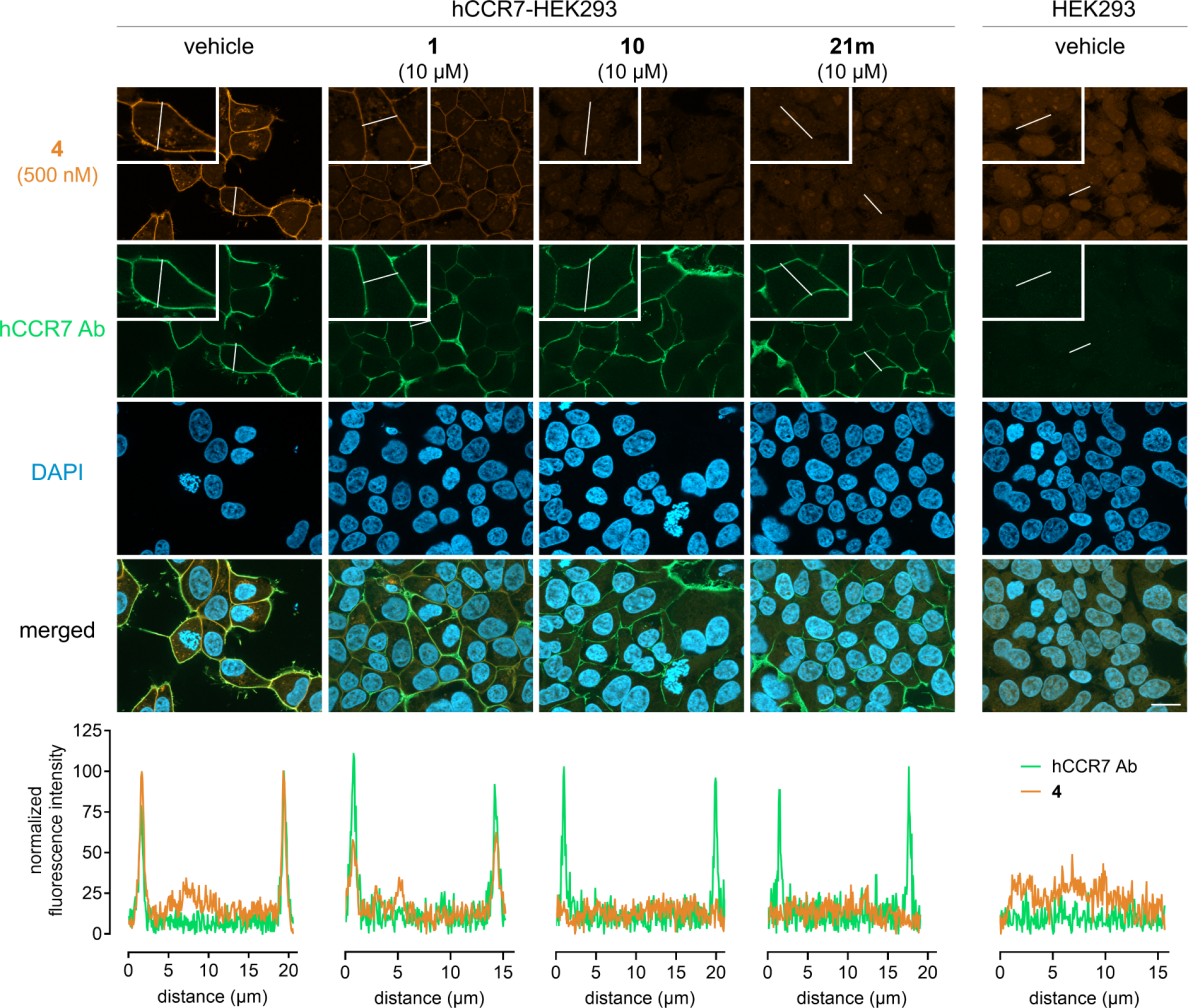
Intracellular CCR7 ligands competitively displace the fluorescent CCR7 probe **4**. Naïve and stable HEK293-CCR7 cells were fixed and immunostained with an anti-CCR7 antibody and with the fluorescent CCR7 probe **4** as indicated. Fluorescence intensity profiles along the white lines were quantified by line scan analysis in the representative images, showing clear colocalization of **4** and receptor immunostaining on the plasma membrane as well as the displacement of the probe by the intracellular CCR7 ligands. Scale bar is 10 μm.

Development of IABS targeting CCR7 ligands demands complementary methods to determine intracellular target engagement. Based on the results from the SAR study, we selected the thiadiazole-1,1-dioxide **10** and the squaramide **21m** as well as Cmp2105 (**1**) as intracellular competitors for our newly developed fluorescent probe **4** using fluorescence microscopy. Distinct from and complementary to NanoBRET-based binding studies, which require the use of labeled receptors, fluorescence microscopy enables visualization of ligand binding to unlabeled receptors. We used fixed naïve and stable CCR7-HEK293 cells to obtain fluorescence profiles in costaining experiments with a CCR7-directed fluorescent antibody and the fluorescent probe Mz437. We found clear fluorescence overlap at the plasma membrane which is evident from both the merged images and the two coincident sharp fluorescence peaks in line scan analysis (Figure 6). No detectable plasma membrane-associated fluorescence was observed when the primary antibody was omitted in the staining protocol (Figure S3) and in naïve HEK293 cells for either the probe or the CCR7 antibody, entirely consistent with CCR7-specific labeling by **4** and the absence of endogenous CCR7 (Figure S4). To test the competitive nature of this binding interaction, we added **4** to cells previously incubated with **10**, **21m**, Cmp2105 (**1**) or vehicle control for 60 min. We observed complete reduction of plasma membrane fluorescence by **10** and **21m**, consistent with specific and quantitative probe displacement from plasma membrane-resident CCR7. Cmp2105 (**1**), on the contrary, only partially promoted probe displacement in agreement with its lower CCR7 affinity as determined in the earlier NanoBRET-based binding assays. Neither of the intracellular antagonists diminished CCR7 recognition by the CCR7-directed fluorescent antibody. These data prove that **4** is a suitable tool for visualizing cellular target engagement of nonfluorescent intracellular CCR7 ligands and reveal that **10** and **21m** clearly surpass the only available reference Cmp2105 (**1**) in binding to CCR7.

### 10 and 21m are Efficacious CCR7 Inhibitors in Recombinant and Primary Cells

**Figure 7:**
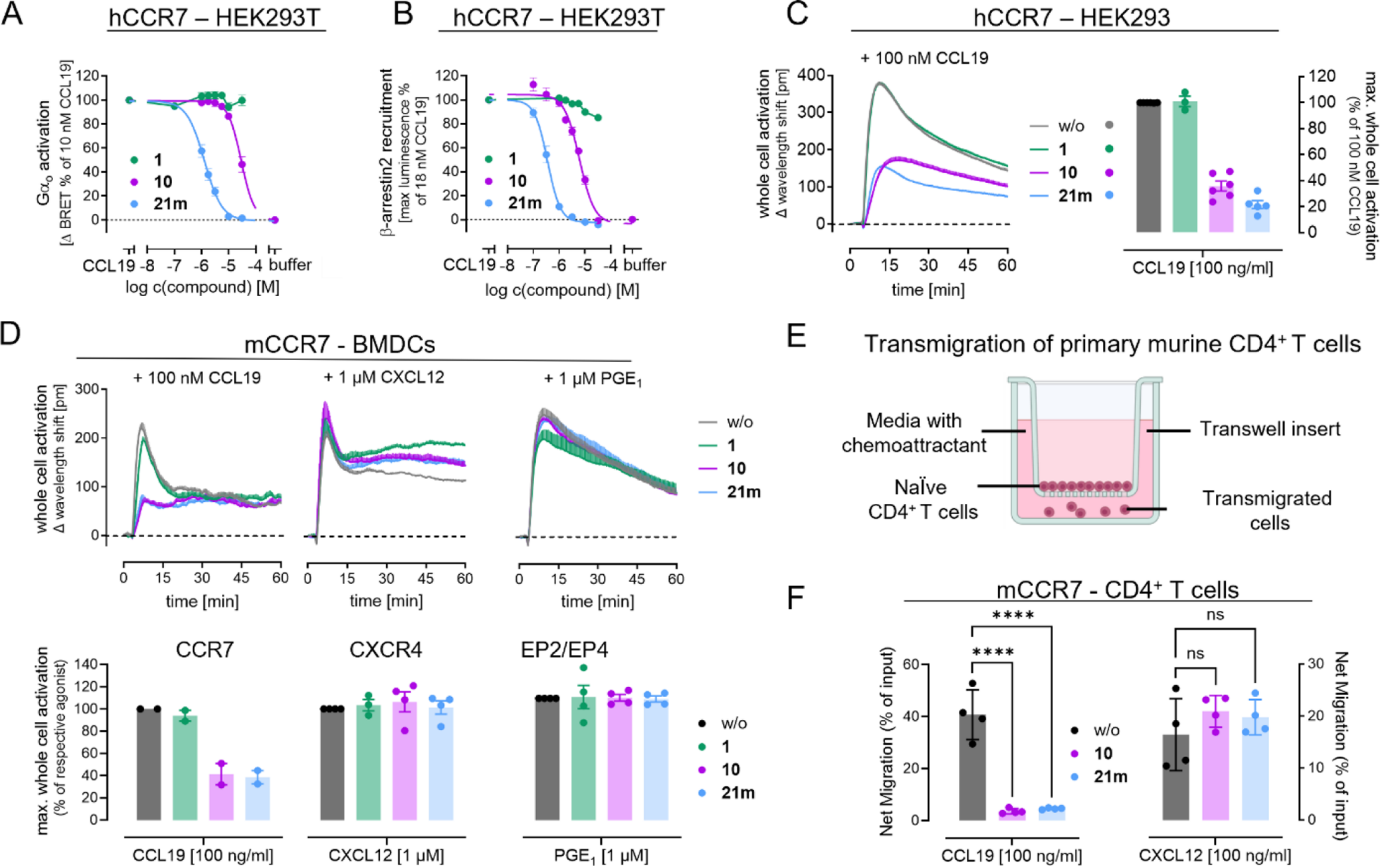
**10** and **21m** dampen CCR7 functionality in recombinant and primary cells. (A) Inhibition of CCL19 (10 nM)-mediated Go activation, determined as BRET between Gαo-derived Gβγ (Venus-labeled) and the Gβγ effector mimic masGRK3ct-Nluc by CCR7 antagonists. MasGRK3ct-Nluc denotes the C-terminal fragment of the G protein-coupled receptor kinase isoform 3 fused to a myristic acid attachment peptide (mas) and NanoLuc luciferase (Nluc); **10** (pIC_50_ = 4.53 ± 0.03 [29.4 μM]) and **21m** (pIC_50_ = 5.89 ± 0.03 [1.28 μM]). (B) Inhibition of CCL19-induced β-arrestin2 recruitment using a NanoBiT-based β-arrestin2 recruitment assay after stimulation of hCCR7 with 18 nM CCL19 (EC_25_); **10** (pIC_50_ = 5.22 ± 0.03 [6.0 μM]) and **21m** (pIC_50_ = 6.45 ± 0.04 [0.35 μM]). (C) Representative whole cell activation profiles and their bar chart summaries of 100 nM CCL19 in the absence and presence of the indicated CCR7 ligands, obtained with label-free DMR whole cell biosensing in HEK293 cells stably expressing human CCR7. Label-free signatures are means + SEM of *n* = 3 experiments, performed in technical quadruplicates. Bar charts are the means ± SEM of *n* = 3 experiments. (D) Representative DMR traces and their bar chart summaries evoked by 100 nM CCL19 in murine BMDCs after preincubation with the indicated CCR7 ligands. Label-free signatures are mean + SEM of technical triplicates. Summarized data are means ± SEM of *n* = 2–4 independent experiments. (E) Experimental setup of transmigration assay. (F) Inhibition of CCR7-mediated transmigration of murine CD4^+^ T cells toward a 100 ng/mL CCL19 or 100 ng/mL CXCL12 gradient by **10** and **21m**. Final concentration of **1**, **10**, and **21m** in C, D, and F is 10 μM. Statistical significance was calculated with a one-way ANOVA with Dunnett̀s posthoc analysis; ****, *p* < 0.0001; ns, not significant. Cartoon created with BioRender.com.

To investigate whether intracellular CCR7 binding also translates to inhibition of CCR7 function, we tested whether **10**, **21m**, and Cmp2105 (**1**) impede CCR7 interaction with signal transducing G proteins and signal-regulating arrestins. In HEK293T cells transiently expressing human CCR7 (hCCR7) increasing concentrations of CCL19 resulted in a concentration-dependent Go activation (Figure S5). Pretreatment of cells with increasing concentrations of CCR7 binders prior to stimulation with CCL19 at its EC_80_ yielded single and double digit micromolar inhibition of Go by **10** and **21m**, respectively, but, to our surprise, no detectable inhibition by Cmp2105 (**1**) whatsoever (Figure 7A). We obtained comparable results assessing inhibition of CCL19-induced β-arrestin2 recruitment, in that **21m** surpassed the inhibitory potency of **10** by a factor of 17, while Cmp2105 (**1**) was again not able to mimic the inhibitory effects of the desmethyl compounds (Figures 7B and S6). To avoid any potential bias in our results due to functional selectivity of the synthesized ligands, we took advantage of holistic label-free whole cell biosensing based on detection of dynamic mass redistribution (DMR). Previously, we have shown that DMR is a powerful assay platform reporting whole cell activation upon GPCR stimulation regardless of the primary signaling pathway.^18,37,38^ Stimulation of HEK293 cells stably transfected to express hCCR7 with CCL19 provoked a feature-reach DMR response, that was strictly CCR7-dependent and characterized by a transient sharp rise that is followed by an initial rapid and a second slower descending phase, which is typical for many Gi-coupled receptors (Figures 7C and S7). In this experimental setup, CCL19 activation of CCR7 was diminished by **10** and more so by **21m** but not by Cmp2105 (**1**) (Figure 7C). These data suggest that (i) lack of antagonistic efficacy of Cmp2105 (**1**) in Go activation and β-arrestin2 recruitment assays unlikely results from biased inhibition of only some of the G protein effector pathways downstream of CCR7, and (ii) that **10** and **21m** should qualify to probe contribution of CCR7 to biological responses also in the endogenous signaling environment. Because CCR7 is expressed in mature bone marrow-derived dendritic cells (BMDCs),^39^ we reasoned that **10** and **21m** should impede cell shape change induced by CCL19 but not by CXC chemokine or unrelated lipid stimuli. Indeed, pretreatment of BMDCs with both inhibitors significantly diminished CCL19-mediated cell shape changes but not those induced by CXCL12 and prostaglandin E_1_ (PGE_1_) acting via endogenous CXCR4 and EP2/EP4 receptors, respectively (Figure 7D). These data confirm CCR7 specificity of antagonist action in the label-free assay platform and imply suitability of both ligands to probe CCR7 biology also in primary cell environments. Consistent with specific inhibition of CCR7 in the endogenous setting, **10** and **21m** blunted CCR7- but not CXCR4-dependent transmigration of primary murine CD4^+^ T cells (Figure 7E,F), which are well-documented to express functional CCR7^40^ (Figure S8A). Cmp2105 (**1**), on the contrary, was again without significant inhibitory effect (Figure S8B).

**Figure 8:**
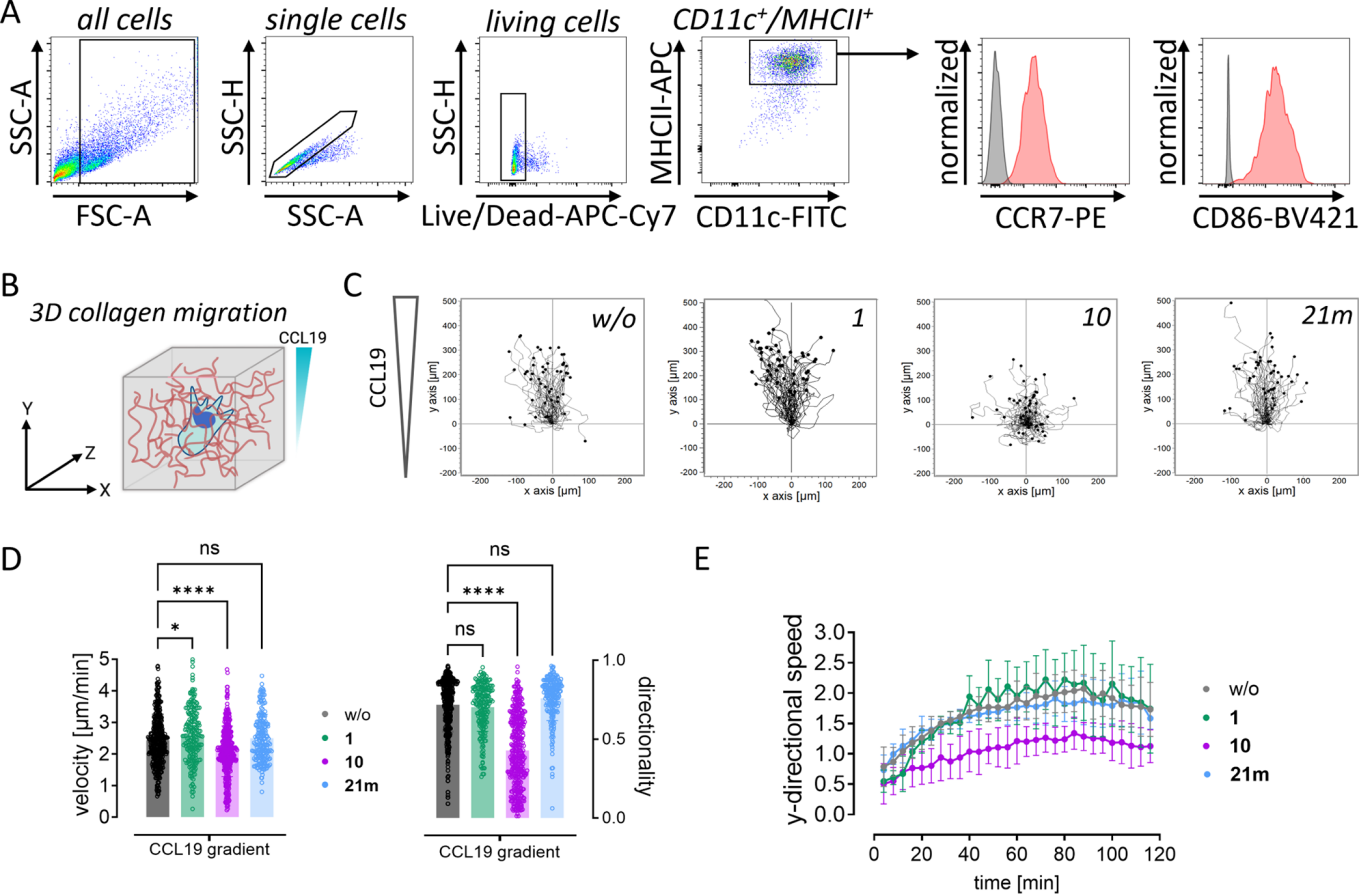
Reconstitution of DC migration in 3D collagen matrices. (A) Characterization of LPS-matured BMDCs by flow cytometry. One representative panel of DC-specific cell surface markers is shown. Unstained samples served as control and were included as light gray filled line. Mature DCs (CD11c^+^/MHCII^+^) show high cell surface levels of CCR7 and CD86 indicating functionality. (B) Schematic representation of 3D collagen migration setup. DCs are embedded within collagen fibers and exposed to a soluble gradient of CCL19. (C) Individual tracks of single cells migrating in the absence (left) or presence of **1** (middle, left), **10** (middle, right), and **21m** (right) each at 10 μM final concentration. Each track represents one cell pooled from two independent experiments. (D) Quantification of migration velocity and directionality (defined as ratio of displacement to trajectory length). Each data point represents one cell. Graph shows mean values ± sd of four independent experiments with cells generated from two different mice. *, *p* < 0.05; ****, *p* < 0.0001 (one-way ANOVA with Dunnett’s posthoc analysis). ns, nonsignificant. (E) Automated analysis of *y*-directional speed of all cells over time. Graph shows mean values ± sd of four independent experiments with cells generated from at least two different mice.

LPS-matured DCs (CD11c^+^/MHCII^+^) show high cell surface levels of CCR7 and CD86 indicating their functionality (Figure 8A). As CCR7-directed migration is an inherent feature of DCs, we used 3D collagen matrices to probe the capacity of BMDCs to migrate along soluble gradients of CCL19 (Figure 8B). Live cell video-microscopy allowed us to follow individual cells over time and extract migration parameters such as velocity and directionality (Figure 8C–E). Manual and automized image analysis confirmed efficient reduction of directional migration toward higher CCL19 concentrations in the presence of **10** (Figure 8D,E), consistent with the role of CCR7 as master regulator of DC migration. By contrast, pretreatment with **21m** or **1** did not impact CCL19-guided BMDC migration (Figure 8D,E). The different behavior of **10** and **21m** in the 3D collagen migration assay, as compared to the results from other functional assays with recombinant and primary cells (see Figure 7) could be due to the complexity of this migration assay in a 3D collagen matrix, where effects such as gel permeation of the compounds might be decisively important for target engagement. Regardless, our data clearly show the antagonistic efficacy of **10** and further emphasize context- and cell type-specific regulation of downstream CCR7 signaling as demonstrated before between T cells and DCs.^39^

## Conclusion

Modulation of CCR7 is therapeutically attractive for two reasons: to dampen immune activation in autoimmune disorders and to improve immunity for antitumor targeting.^16,17^ In this work, we developed the TAMRA-derived fluorescent probe Mz437 (**4**) targeting the IABS of CCR7. We demonstrated its utility in enabling both cell-free and cellular NanoBRET-based binding studies. Additionally, we showed that Mz437 is an effective tool for visualizing intracellular target engagement of CCR7 through fluorescence microscopy. By employing Mz437, we identified the two intracellular CCR7 ligands **10** (hereafter dubbed SLW131) and **21m** (hereafter named SLW132). SLW131, a thiadiazoledioxide, was developed from Cmp2105 (**1**) by removing a methyl group from the benzamide core. In contrast, SLW132, a squaramide, was obtained from the CXCR1/CXCR2 antagonist navarixin by replacing its ethyl group with a *tert*-butyl group to target a lipophilic subpocket. Both compounds (SLW131 and SLW132) exhibit improved binding affinities and antagonistic properties compared to the prior gold standard intracellular CCR7 ligand Cmp2105 (**1**) that was largely inactive in our cell-based activity assays. In detail, CCR7 G protein activation and β-arrestin recruitment assays revealed that the increased affinity of SLW131 and SLW132 translates into an improved antagonistic behavior compared to Cmp2105 (**1**), which was further corroborated by label-free whole cell biosensing based on detection of dynamic mass redistribution in both stable CCR7-HEK293 cells and primary BMDCs.

In conclusion, we successfully identified SLW131 and SLW132 as promising leads for future studies of CCR7 pharmacology and function in both recombinant and endogenous signaling environments. It is anticipated that these molecular tools will considerably advance our understanding of CCR7 functionality.

## Experimental Section

### Chemistry

#### General Remarks

Chemicals were obtained from abcr GmbH (Karlsruhe, Germany), Acros Organics (Geel, Belgien), Carbolution Chemicals (Sankt Ingberg, Germany), Sigma-Aldrich (Steinheim, Germany,) TCI Chemicals (Eschborn, Germany) or VWR (Langenfeld, Germany) and used without further purification. Cmp2105 (**1**) was obtained from Hölzel Diagnostika (Köln, Germany). Technical-grade solvents were distilled prior to use. For all HPLC purposes, acetonitrile in HPLC-grade quality (HiPerSolv CHROMANORM, VWR, Langenfeld, Germany) was used. Water was purified with a PURELAB flex (ELGA VEOLIA, Celle, Germany). Thin-layer chromatography (TLC) was carried out on prefabricated plates (silica gel 60, F254, Merck). Components were visualized either by irradiation with ultraviolet light (254 or 366 nm) or by appropriate staining. Column chromatography was carried out on silica gel (60 Å, 40–60 μm, Acros Organics, Geel, Belgium). If no solvent is stated, an aqueous solution was prepared with demineralized water. Mixtures of two or more solvents are specified as “solvent A”/“solvent B”, 3/1, *v*/*v*; meaning that 100 mL of the respective mixture consists of 75 mL of “solvent A” and 25 mL of “solvent B”. The uncorrected melting points were determined using a Büchi (Essen, Germany) Melting Point *M*-560 apparatus. Diastereomeric ratios were determined by ^1^H NMR spectroscopy. Proton (^1^H) and carbon (^13^C) NMR spectra were recorded either on a Bruker AVANCE 500 MHz at a frequency of 500 MHz (^1^H) and 126 MHz (^13^C) or a Bruker AVANCE III HD 600 MHz at a frequency of 600 MHz (^1^H) and 151 MHz (^13^C). The chemical shifts are given in parts per million (ppm). As solvents, deuterated chloroform (CDCl_3_), deuterated methanol (methanol-*d*_4_) and deuterated dimethyl sulfoxide (DMSO-*d*_6_) were used. The residual solvent signal (CDCl_3_: ^1^H NMR: 7.26 ppm, ^13^C NMR: 77.1 ppm; DMSO-*d*_6_: ^1^H NMR: 2.50 ppm, ^13^C NMR: 39.52 ppm; methanol-*d*_4_: ^1^H NMR: 3.31 ppm, 4.87 ppm, ^13^C NMR: 49.00 ppm) was used for calibration. The multiplicity of each signal is reported as singlet (s), doublet (d), triplet (t), quartet (q), multiplet (m) or combinations thereof. Multiplicities and coupling constants are reported as measured and might disagree with the expected values. High-resolution electrospray-ionization mass spectra (HRMS-ESI) were acquired with a Bruker Daltonik GmbH micrOTOF coupled to a an LC Packings Ultimate HPLC system and controlled by micrOTOFControl3.4 and HyStar 3.2-LC/MS, with a Bruker Daltonik GmbH ESI-qTOF Impact II coupled to a Dionex UltiMateTM 3000 UHPLC system and controlled by micrOTOFControl 4.0 and HyStar 3.2-LC/MS or with a micrOTOF-Q mass spectrometer (Bruker, Bremen, Germany) with ESI-source coupled with an HPLC Dionex UltiMate 3000 (Thermo Scientific, Heysham, United Kingdom). Low-resolution electrospray-ionization mass spectra (LRMS-ESI) were acquired with an Advion expression compact mass spectrometer (CMS) coupled with an automated TLC plate reader Plate Express (Advion, Ithaca, NY, USA). A Thermo Fisher Scientific (Heysham, United Kingdom) UltiMateTM 3000 UHPLC system with a Nucleodur 100–5 C18 (250 × 4.6 mm, Macherey Nagel, Düren, Germany) with a flow rate of 1 mL/min and a temperature of 25 °C, or a 100–5 C18 (100 × 3 mm, Macherey Nagel, Düren, Germany) with a flow rate of 0.5 mL/min and a temperature of 25 °C was used with an appropriate gradient. For preparative purposes, an AZURA Prep. 500/1000 gradient system with a Nucleodur 110–5 C18 HTec (150 × 32 mm, Macherey Nagel, Düren, Germany) column with 20 mL/min was used. Detection was implemented with UV absorption measurement at wavelengths of λ = 220 nm and λ = 250 nm. Bidest. H_2_O (A) and MeCN (B) were used as eluents with an addition of 0.1% TFA in case of eluent A. Purity: The purity of all final compounds was 95% or higher. Purity analysis for compound **4** was performed using an Agilent 1200 series HPLC system employing a diode array detector (DAD, detection at 200, 220, 254, or 560 nm) and a ZORBAX ECLIPSE, XDB-C8 column (4.6 mm × 150 mm, 5 μm) with a flow rate of 0.5 mL·min^–1^. The indicated purity was determined at a wavelength of 254 nm. As solvent systems the following binary solvent systems were used. Elution was performed at room temperature under gradient conditions. Eluent A was water containing 0.1% (v/v) TFA; eluent B was acetonitrile. Linear gradient conditions were as follows: 0–3.0 min: A = 90%, B = 10%; 3.0–18.0 min: linear increase to A = 5%, B = 95%; 18.0–24.0 min: A = 5%, B = 95%; 24.0–27.0 min: linear decrease to A = 90%, B = 10%; 27.0–30.0 min: A = 90%, B = 10%. Purity analyses for compounds **3**, **10**, **20a**–**d**, **21a**–**m**, were performed on a Thermo Fisher Scientific UltiMateTM 3000 UHPLC system with a Nucleodur 100-5 C18 (250 × 4.6 mm, Macherey Nagel, Düren, Germany) at 250 nm. Elution was performed at a flow rate of 1 mL/min and a temperature of 25 °C. After column equilibration for 5 min, a linear gradient from 5% A to 95% B in 7 min followed by an isocratic regime of 95% B for 10 min was used.

### Synthesis and Compound Characterization

Detailed information on experimental procedures for compound synthesis as well as compound characterization data are listed below. NMR spectra and HPLC chromatograms are given in the Supporting Information. Compounds **22a**–**i** were synthesized as previously published by us.^10^ Compounds **11c**, **13a**, **14a**–**c**, **15a**–**c**, **16a,b**, **17a,b**, **19a,b**, and 3,4-dimethoxy-1,2,5-thiadiazole-1,1-dioxide were synthesized according to previously published procedures.^10,11,20,41,42^

#### (*R*)-3-[(4-{[2,2-Dimethyl-1-(5-methylfuran-2-yl)propyl]amino}-1,1-dioxido-1,2,5-thiadiazol-3-yl)amino]-2-hydroxy-*N*-{[1-(2-methoxyethyl)-1*H*–1,2,3-triazol-4-yl]methyl}benzamide (**3**)

(*R*)-3-[(4-{[2,2-Dimethyl-1-(5-methylfuran-2-yl)propyl]amino}-1,1-dioxido-1,2,5-thiadiazol-3-yl)amino]-2-hydroxy-*N*-(prop-2-yn-1-yl)benzamide (**20b,** 10 mg, 0.097 mmol, 1.0 *eq*) and 1-azido-2-methoxyethane (48 mg, 0.097 mmol, 1.0 *eq*) were dissolved in a water/*tert*-BuOH mixture (2 mL, 1:1). Tris(benzyltriazolylmethyl)amine (TBTA, 5.1 mg, 0.010 mmol, 0.10 *eq*) was dissolved in DMF (1 mL) and added to the mixture. An aqueous CuSO_4_ solution (0.1 M, 0.48 mL, 0.048 mmol, 0.50 *eq*) and an aqueous solution of sodium ascorbate (0.1 M, 0.24 mL, 0.024 mmol, 0.25 *eq*) were added in that order. The resulting reaction mixture was stirred for 2 h at room temperature. After completion of the reaction, volatiles were removed under reduced pressure. The residue was purified by preparative HPLC (acetonitrile/water (0.1%TFA): gradient 5–95%) to obtain the title compound as a colorless solid (20 mg, 36%). ^1^H NMR (500 MHz, DMSO-*d*_6_, δ [ppm]): 13.72 (s, 1H), 10.59 (s, 1H), 9.62 (t, *J* = 5.8 Hz, 1H), 9.25 (d, *J* = 9.1 Hz, 1H), 8.00 (s, 1H), 7.87 (d, *J* = 7.9 Hz, 1H), 7.01 (t, *J* = 8.0 Hz, 1H), 6.32 (d, *J* = 3.1 Hz, 1H), 6.07 (dd, *J* = 3.1, 1.2 Hz, 1H), 4.87 (d, *J* = 9.0 Hz, 1H), 4.59 (d, *J* = 5.8 Hz, 2H), 4.50 (t, *J* = 5.2 Hz, 2H), 3.72 (t, *J* = 5.2 Hz, 2H), 3.23 (s, 3H), 2.30 (s, 2H), 1.01 (s, 9H); ^13^C NMR (126 MHz, DMSO-*d*_6_, δ [ppm]): 169.3, 155.8, 154.0, 153.4, 151.3, 149.3, 143.8, 129.6, 125.5, 125.2, 123.4, 118.1, 115.0, 109.6, 106.4, 70.1, 61.8, 57.9, 49.1, 35.7, 34.7, 26.5, 13.4; HRMS *m*/*z* (ESI^+^) [found: 573.2238, C_25_H_33_N_8_O_6_S^+^ requires [M + H]^+^ 573.2166]; HPLC retention time: 12.45 min, purity: 95.1%.

#### (*R*)-4-((2-(2-(2-(2-(4-((3-((4-((2,2-Dimethyl-1-(5-methylfuran-2-yl)propyl)amino)-1,1-dioxido-1,2,5-thiadiazol-3-yl)amino)-2-hydroxybenzamido)methyl)-1*H*-1,2,3-triazol-1-yl)ethoxy)ethoxy)ethoxy)ethyl)carbamoyl)-2-(6-(dimethylamino)-3-(dimethyliminio)-3*H*-xanthen-9-yl)benzoate (Mz437, **4**)

(*R*)-3-((4-((2,2-Dimethyl-1-(5-methylfuran-2-yl)propyl)amino)-1,1-dioxido-1,2,5-thiadiazol-3-yl)amino)-2-hydroxy-*N*-(prop-2-yn-1-yl)benzamide (**20b**, 4.0 mg, 8.5 μmol, 1.0 *eq*.), the 6-TAMRA-PEG3-azide (5.4 mg, 8.5 μmol, 1.0 *eq*.), and tris(benzyltriazolylmethyl)amine (TBTA, 0.50 mg, 0.85 μmol, 0.10 *eq*) were dissolved in a water/*tert*-BuOH/DMF mixture (1.5 mL, 1:1:1 (v/v/v)). An aqueous CuSO_4_ solution (8.5 μL, 0.10 M, 0.10 *eq*.) and an aqueous solution of sodium ascorbate (17 μL, 0.10 M, 0.20 *eq*.) were added in that order. The resulting reaction mixture was stirred for 6 h at room temperature under nitrogen atmosphere. After completion, volatiles were removed under reduced pressure. The residue was purified by preparative HPLC (acetonitrile/water (0.1% TFA): gradient 30% – 55%) to obtain the TFA salt of title compound as a pink solid (5.4 mg, 52%). ^1^H NMR (600 MHz, DMSO-*d*_6_, δ [ppm]): 13.71 (s, 1H), 13.36 (s, 1H), 10.60 (s, 1H), 9.61 (t, *J* = 5.7 Hz, 1H), 9.26 (d, *J* = 9.0 Hz, 1H), 8.79 (t, *J* = 5.5 Hz, 1H), 8.32–8.25 (m, 1H), 8.22 (d, *J* = 8.2 Hz, 1H), 7.99 (s, 1H), 7.89–7.81 (m, 3H), 7.18–6.79 (m, 6H), 6.98 (t, *J* = 8.0 Hz, 1H), 6.31 (d, *J* = 3.1 Hz, 1H), 6.07 (dq, *J* = 3.1 Hz, 1.1 Hz, 1H), 4.86 (d, *J* = 9.0 Hz, 1H), 4.54 (d, *J* = 5.7 Hz, 2H), 4.46 (t, *J* = 5.3 Hz, 2H), 3.76 (t, *J* = 5.3 Hz, 2H), 3.50^#^ (t, *J* = 5.9 Hz, 2H), 3.47–3.40^#^ (m, 10H), 3.24 (s, 12H), 2.29 (d, *J* = 1.1 Hz, 3H), 1.00 (s, 9H); ^13^C NMR*^a^* (DEPTQ, 151 MHz, DMSO*-d*_6_, δ [ppm]): 169.3, 164.5, 157.6 (q, *J* = 32.6 Hz), 155.8, 154.0, 153.4, 151.3, 149.3, 143.8, 133.0*, 130.7*, 130.6, 129.6, 129.0, 128.6*, 125.5, 125.2, 123.5, 118.1, 114.4*, 114.9, 109.7, 106.4, 96.3, 69.6, 69.6, 69.5, 69.5, 68.7, 68.6, 61.8, 49.3, 40.4, 39.4*^#^, 35.7, 34.6, 26.6, 13.4; *^a^*the ^13^C NMR (DEPTQ) signals for 3*H*-xanthene C-3,4a,6,8a,9,9a,10a, carbamoyl benzoate C-2,4, −COOH, and F_3_C-COO^–^ could not be detected. *Could only be detected in HSQC/HMBC spectra; ^#^partially overlaid by solvent signal. HRMS *m*/*z* (ESI^+^) [found: 1102.4455, C_55_H_64_N_11_O_12_S^+^ requires [M + H]^+^ 1102.4451]; HPLC retention time: 17.63 min, 95.3%).

#### (*R*)-3-[(4-{[2,2-Dimethyl-1-(5-methylfuran-2-yl)propyl]amino}-1,1-dioxido-1,2,5-thiadiazol-3-yl)amino]-2-hydroxy-*N,N*-dimethylbenzamide (SLW131, **10**)

2-Hydroxy-3-[(4-methoxy-1,1-dioxido-1,2,5-thiadiazol-3-yl)amino]-*N,N*-dimethylbenzamide (**18a**, 50 mg, 0.20 mmol, 1.0 *eq*) and (*R*)-2,2-dimethyl-1-(5-methylfuran-2-yl)propan-1-amine hydrochloride (**15c**, 31 mg, 0.20 mmol, 1.0 *eq*) were dissolved in methanol (10 mL). After the addition of *N,N*-diisopropylethylamine (54 μL, 0.40 mmol, 2.0 *eq*), the reaction was stirred for 3 days. Extraction between water and ethyl acetate (3 × 50 mL), drying over sodium sulfate, filtration, and evaporation of the solvent gave the crude product which was purified by preparative HPLC (acetonitrile/water (0.1% TFA): gradient 5–95%) to obtain the title compound as a white-brown amorphous solid (17 mg, 24%). ^1^H NMR (500 MHz, DMSO-*d*_6_, δ [ppm]): 10.48 (s, 1H), 9.88 (s, 1H), 8.89 (d, *J* = 9.1 Hz, 1H), 7.46 (dd, *J* = 7.9, 1.7 Hz, 1H), 7.12 (dd, *J* = 7.6, 1.7 Hz, 1H), 6.96 (t, *J* = 7.8 Hz, 1H), 6.32 (d, *J* = 3.1 Hz, 1H), 6.07 (dd, *J* = 3.2, 1.3 Hz, 1H), 4.85 (d, *J* = 9.1 Hz, 1H), 2.92 (s, 6H), 2.29 (s, 3H), 1.02 (s, 9H); ^13^C NMR (126 MHz, DMSO-*d*_6_, δ [ppm]): 167.7, 156.0, 154.6, 151.2, 149.4, 147.3, 127.3, 126.8, 126.5, 124.5, 119.7, 109.5, 106.3, 61.6, 35.7, 26.5, 23.9, 13.4; HRMS *m*/*z* (ESI^+^) [found: 462.1806, C_21_H_28_N_5_O_5_S ^+^ requires [M + H]^+^ 462.1766]; HPLC retention time: 12.33 min, purity: 95.6%.

#### (*S,E*)-2-Methyl-*N*-[(5-methylthiophen-2-yl)methylene]propane-2-sulfinamide (**13b**)

(*S*)-2-Methylpropane-2-sulfinamide (1.00 g, 8.09 mmol, 1.00 *eq*.) was dissolved in dichloromethane (10 mL). 5-Methylthiophene-2-carboxaldehyde (918 μL, 8.09 mmol, 1.00 *eq*.), titanium ethoxide (3.73 mL, 17.8 mmol, 2.20 *eq*), and sodium sulfate (2 g) were added under stirring. The reaction mixture was stirred at room temperature overnight, filtered through Celite, and rinsed with dichloromethane. Evaporation of the solvent gave the crude product in quantitative yield, which was used without further purification.

#### (*S,E*)-2-Methyl-*N*-(pyrimidin-5-ylmethylene)propane-2-sulfinamide (**13c**)

(*S*)-2-Methylpropane-2-sulfinamide (1.00 g, 8.09 mmol, 1.00 *eq*.) was dissolved in dichloromethane (10 mL). Pyrimidine-5-carboxaldehyde (920 mg, 8.09 mmol, 1.00 *eq*.), titanium ethoxide (3.73 mL, 17.8 mmol, 2.20 *eq*), and sodium sulfate (2 g) were added under stirring. The reaction mixture was stirred at room temperature overnight, filtered through Celite, and rinsed with dichloromethane. Evaporation of the solvent gave the crude product in quantitative yield, which was used without further purification.

#### (*S,E*)-*N*-[(2,4-Dichlorothiazol-5-yl)methylene]-2-methylpropane-2-sulfinamide (**13d**)

(*S*)-2-Methylpropane-2-sulfinamide (650 mg, 5.26 mmol, 1.00 *eq*.) was dissolved in dichloromethane (10 mL). 2,4-Dichlorothiazole-5-carboxaldehyde (1.01 g, 5.26 mmol, 1.00 *eq*.), titanium ethoxide (2.42 mL, 11.6 mmol, 2.20 *eq*), and sodium sulfate (2 g) were added under stirring. The reaction mixture was stirred at room temperature overnight, filtered through Celite, and rinsed with dichloromethane. Evaporation of the solvent gave the crude product in quantitative yield, which was used without further purification.

#### (*S,E*)-*N*-{[5-(4-Chlorophenyl)isoxazol-3-yl]methylene}-2-methylpropane-2-sulfinamide (**13e**)

(*S*)-2-Methylpropane-2-sulfinamide (600 mg, 4.85 mmol, 1.00 *eq*.) was dissolved in dichloromethane (10 mL). 5-(4-Chlorophenyl)isoxazole-3-carboxaldehyde (1.06 g, 4.85 mmol, 1.00 *eq*.), titanium ethoxide (2.24 mL, 10.7 mmol, 2.20 *eq*), and sodium sulfate (2 g) were added under stirring. The reaction mixture was stirred at room temperature overnight, filtered through Celite, and rinsed with dichloromethane. Evaporation of the solvent gave the crude product in quantitative yield, which was used without further purification.

#### (*S*)-*N*-[(*R*)-2,2-Dimethyl-1-(5-methylthiophen-2-yl)propyl]-2-methylpropane-2-sulfinamide (**14d**)

A 250 mL flask was sealed with a septum flushed with N_2_ and filled with THF (20 mL). A 1 M *tert-*butylmagnesium chloride solution in THF (18.0 mL, 17.8 mmol, 2.20 *eq*) was added and the solution was cooled to 0 °C. (*S,E*)-2-Methyl-*N*-[(5-methylthiophen-2-yl)methylene]propane-2-sulfinamide (**13b**, 1.86 g, 8.10 mmol, 1.00 *eq*) was dissolved in THF (20 mL) and added dropwise to the vigorously stirred solution. After addition, the mixture was stirred for 72 h. The reaction mixture was then quenched by the addition of saturated ammonium chloride solution (50 mL) and extracted using ethyl acetate (3 × 100 mL). Drying over sodium sulfate, filtration, and evaporation of the solvent resulted in the crude product which was purified by column chromatography using a gradient of dichloromethane and ethyl acetate (dichloromethane to dichloromethane/ethyl acetate (8/2) to dichloromethane/ethyl acetate (5/5) (*v/v*)). The title compound was obtained as a dark orange to red oil (264 mg, 11%). ^1^H NMR (500 MHz, DMSO-*d*_6_ δ [ppm]) 6.77 (d, *J* = 3.4 Hz, 1H), 6.63 (dq, *J* = 3.4, 1.2 Hz, 1H), 4.40 (d, *J* = 4.4 Hz, 1H), 4.18 (d, *J* = 4.4 Hz, 1H), 2.39 (d, *J* = 1.1 Hz, 3H), 1.08 (s, 9H), 0.94 (s, 9H); ^13^C NMR (126 MHz, DMSO-*d*_*6*_ δ [ppm]) 140.9, 138.1, 126.7, 124.2, 64.3, 55.2, 35.1, 26.5, 22.3, 14.9; LRMS *m*/*z* (ESI^+^) [found: 288.5, C_14_H_26_NOS_2_^+^ requires [M + H]^+^ 288.5]; *R*_f_: 0.34 (cyclohexane/ethyl acetate (1/1) (*v/v*)); diastereomeric ratio: *dr* = 1:0.

#### (*S*)-*N*-[(*R*)-2,2-Dimethyl-1-(pyrimidin-5-yl)propyl]-2-methylpropane-2-sulfinamide (**14e**)

A 250 mL flask was sealed with a septum flushed with N_2_ and filled with THF (20 mL). A 1 M *tert-*butylmagnesium chloride solution in THF (18.0 mL, 17.8 mmol, 2.20 *eq*) was added and the solution was cooled to 0 °C. (*S,E*)-2-methyl-*N*-(pyrimidin-5-ylmethylene)propane-2-sulfinamide (**13c**, 1.71 g, 8.10 mmol, 1.00 *eq*) was dissolved in THF (20 mL) and added dropwise to the vigorously stirred solution. After addition, the mixture was stirred for 72 h. The reaction mixture was then quenched by the addition of saturated ammonium chloride solution (50 mL) and extracted using ethyl acetate (3 × 100 mL). Drying over sodium sulfate, filtration, and evaporation of the solvent resulted in the crude product which was purified by column chromatography using a gradient of dichloromethane and ethyl acetate (dichloromethane to dichloromethane/ethyl acetate (8/2) to dichloromethane/ethyl acetate (5/5) (*v/v*)). The title compound was obtained as a dark orange to red oil (115 mg, 5%). ^1^H NMR (500 MHz, DMSO-*d*_6_ δ [ppm]) 9.06 (s, 1H), 8.77 (s, 2H), 5.17 (d, *J* = 7.5 Hz, 1H), 4.16 (d, *J* = 7.6 Hz, 1H), 1.08 (s, 9H), 0.93 (s, 9H); ^13^C NMR (126 MHz, DMSO-*d*_6_ δ [ppm]) 157.0, 156.6, 134.4, 64.9, 55.5, 53.9, 35.2, 26.3, 22.2; LRMS *m*/*z* (ESI^+^) [found: 288.5, C_13_H_24_N_3_OS^+^ requires [M + H]^+^ 270.4]; *R*_f_: 0.20 (cyclohexane/ethyl acetate (1/1) (*v/v*)); diastereomeric ratio: *dr* = 1:0.

#### (*S*)-*N*-[(*R*)-1-(2,4-Dichlorothiazol-5-yl)-2,2-dimethylpropyl]-2-methylpropane-2-sulfinamide (**14f**)

A 250 mL flask was sealed with a septum flushed with N_2_ and filled with THF (20 mL). A 1 M *tert-*butylmagnesium chloride solution in THF (12.0 mL, 11.6 mmol, 2.20 *eq*) was added and the solution was cooled to 0 °C. (*S,E*)-*N*-[(2,4-dichlorothiazol-5-yl)methylene]-2-methylpropane-2-sulfinamide (**13d**, 1.5 g, 5.3 mmol, 1 *eq*) was dissolved in THF (20 mL) and added dropwise to the vigorously stirred solution. After addition, the mixture was stirred for 72 h. The reaction mixture was then quenched by the addition of saturated ammonium chloride solution (50 mL) and extracted using ethyl acetate (3 × 100 mL). Drying over sodium sulfate, filtration, and evaporation of the solvent resulted in the crude product which was purified by column chromatography using a gradient of dichloromethane and ethyl acetate (dichloromethane to dichloromethane/ethyl acetate (8/2) to dichloromethane/ethyl acetate (5/5) (*v/v*)). The title compound was obtained as a dark orange to red oil (447 mg, 25%). ^1^H NMR (500 MHz, DMSO-*d*_6_ δ [ppm]) 4.97 (d, *J* = 5.2 Hz, 1H), 4.40 (d, *J* = 5.2 Hz, 1H), 1.08 (s, 9H), 1.00 (s, 9H); ^13^C NMR (126 MHz, DMSO-*d*_6_ δ [ppm]) 150.2, 134.9, 134.1, 61.9, 55.8, 36.2, 26.1, 22.2; LRMS *m*/*z* (ESI^+^) [found: 345.5, C_12_H_21_Cl_2_NOS_2_^+^ requires [M + H]^+^ 345.3.]; *R*_f_: 0.51 (cyclohexane/ethyl acetate (1/1) (*v/v*)); diastereomeric ratio: *dr* = 1:0.

#### (*S*)-*N*-(*R*)-1-[5-(4-Chlorophenyl)isoxazol-3-yl)-2,2-dimethylpropyl]-2-methylpropane-2-sulfinamide (**14g**)

A 250 mL flask was sealed with a septum flushed with N_2_ and filled with THF (20 mL). A 1 M *tert-*butylmagnesium chloride solution in THF (11.2 mL, 11.2 mmol, 2.20 *eq*) was added and the solution was cooled to 0 °C. (*S,E*)-*N-{*[5-(4-Chlorophenyl)isoxazol-3-yl]methylene}-2-methylpropane-2-sulfinamide (**13e**, 1.6 g, 5.1 mmol, 1.0 *eq*) was dissolved in THF (20 mL) and added dropwise to the vigorously stirred solution. After addition, the mixture was stirred for 72 h. The reaction mixture was then quenched by the addition of saturated ammonium chloride solution (50 mL) and extracted using ethyl acetate (3 × 100 mL). Drying over sodium sulfate, filtration, and evaporation of the solvent resulted in the crude product which was purified by column chromatography using a gradient of dichloromethane and ethyl acetate (dichloromethane to dichloromethane/ethyl acetate (8/2) to dichloromethane/ethyl acetate (5/5) (*v/v*)). The title compound was obtained as a dark orange to red oil (447 mg, 25%). ^1^H NMR (500 MHz, DMSO-*d*_6_ δ [ppm]) 7.89–7.80 (m, 2H), 7.66–7.57 (m, 2H), 7.14 (s, 1H), 5.15 (d, *J* = 10.1 Hz, 1H), 4.10 (d, *J* = 10.1 Hz, 1H), 1.16 (s, 9H), 0.95 (s, 9H); ^13^C NMR (126 MHz, DMSO-*d*_6_ δ [ppm]) 166.9, 165.0, 134.9, 129.4, 127.2, 125.7, 100.9, 61.8, 56.2, 35.3, 26.5, 22.7; LRMS *m*/*z* (ESI^+^) [found: 369.5, C_18_H_26_ClNO_2_S^+^ requires [M + H]^+^ 369.9]; *R*_f_: 0.51 (cyclohexane/ethyl acetate (1/1) (*v/v*)); diastereomeric ratio: *dr* = 1:0.

#### (*R*)-2,2-Dimethyl-1-(5-methylthiophen-2-yl)propan-1-amine hydrochloride (**15d**)

(*S*)-*N*-[(*R*)-2,2-Dimethyl-1-(5-methylthiophen-2-yl)propyl]-2-methylpropane-2-sulfinamide (200 mg, 0.700 mmol, 1.00 *eq*) was dissolved in diethyl ether (10 mL) and cooled to 0 °C. After addition of 2 M HCl in diethyl ether (873 μL, 1.75 mmol, 2.50 *eq*) the mixture was stirred for 1 h. After evaporation of the solvents, **15d** was obtained as a reddish oil that was used in the next step without further purification. LRMS *m*/*z* (ESI^+^) [found: 185.3, C_10_H_19_NS^+^ requires [M + H]^+^ 185.3].

#### (*R*)-2,2-Dimethyl-1-(pyrimidin-5-yl)propan-1-amine hydrochloride (**15e**)

(*S*)-*N*-[(*R*)-2,2-Dimethyl-1-(pyrimidin-5-yl)propyl]-2-methylpropane-2-sulfinamide (80 mg, 0.28 mmol, 1.0 *eq*) was dissolved in diethyl ether (10 mL) and cooled to 0 °C. After addition of 2 M HCl in diethyl ether (1.6 mL, 0.70 mmol, 2.5 *eq*) the mixture was stirred for 1 h. After evaporation of the solvents, **15e** was obtained as a reddish oil that was used in the next step without further purification. LRMS *m*/*z* (ESI^+^) [Found: 166.4, C_9_H_17_N_3_^+^ requires [M + H]^+^ 166.2].

#### (*R*)-1-(2,4-Dichlorothiazol-5-yl)-2,2-dimethylpropan-1-amine hydrochloride (**15f**)

(*S*)-*N*-[(*R*)-1-(2,4-Dichlorothiazol-5-yl)-2,2-dimethylpropyl]-2-methylpropane-2-sulfinamide (300 mg, 0.872 mmol, 1.00 *eq*) was dissolved in diethyl ether (10 mL) and cooled to 0 °C. After addition of 2 M HCl in diethyl ether (1.10 mL, 2.18 mmol, 2.50 *eq*) the mixture was stirred for 1 h. After evaporation of the solvents, **15f** was obtained as a reddish oil that was used in the next step without further purification. LRMS *m*/*z* (ESI^+^) [Found: 240.4, C_8_H_14_Cl_2_N_2_S^+^ requires [M + H]^+^ 240.2].

#### (*R*)-1-[5-(4-Chlorophenyl)isoxazol-3-yl]-2,2-dimethylpropan-1-amine hydrochloride (**15g**)

(*S*)-*N*-(*R*)-1-[5-(4-Chlorophenyl)isoxazol-3-yl)-2,2-dimethylpropyl]-2-methylpropane-2-sulfinamide (300 mg, 0.872 mmol, 1.00 *eq*) was dissolved in diethyl ether (10 mL) and cooled to 0 °C. After addition of 2 M HCl in diethyl ether (1.10 mL, 2.18 mmol, 2.50 *eq*) the mixture was stirred for 1 h. After evaporation of the solvents, **15g** was obtained as a reddish oil that was used in the next step without further purification. LRMS *m*/*z* (ESI^+^) [found: 266.8, C_14_H_19_ClN_2_O^+^ requires [M + H]^+^ 266.8].

#### 2-Hydroxy-*N*-methyl-3-nitro-*N*-(prop-2-yn-1-yl)benzamide (**16c**)

2-Hydroxy-3-nitrobenzoic acid (1.00 g, 5.35 mmol, 1.00 *eq*), bromotrispyrrolidinophosphonium hexafluorophosphate (3.02 g, 6.42 mmol, 1.20 *eq*) and *N,N-*diisopropylethylamine (3.78 mL, 21.4 mmol, 4.00 *eq*) were dissolved in dichloromethane (30 mL) and stirred at room temperature for 30 min. *N*-Methylpropargylamine (666 μL, 7.49 mmol, 1.40 *eq*) was added and the resulting mixture was stirred overnight. The reaction was then extracted with 1 N sodium hydroxide solution (3 × 100 mL) and the organic phase was discarded. The aqueous phase was then acidified using 1 M hydrochloric acid (300 mL) and the organic products were extracted using ethyl acetate (3 × 150 mL). The organic phase was dried over sodium sulfate, filtered, and concentrated *in vacuo* to afford the crude product, which was then purified by column chromatography using a mixture of cyclohexane and ethyl acetate (6/4 (v/v)). The title compound was obtained as a pale yellow oil (1.2 g, 95%). ^1^H NMR (500 MHz, DMSO-*d*_6_ δ [ppm]): 10.71 (s, 1H), 8.05 (dd, *J* = 8.3, 1.7 Hz, 1H), 7.56 (dd, *J* = 7.5, 1.7 Hz, 1H), 7.11 (dd, *J* = 8.4, 7.4 Hz, 1H), 4.32 (s, 1H), 3.39–3.21 (m, 2H), 3.10–2.81 (m, 3H); ^13^C NMR (151 MHz, DMSO-*d*_6_ δ [ppm]): 166.0, 148.6, 136.2, 134.3, 134.2, 128.4, 125.9, 120.1, 78.9, 74.6, 35.3; LRMS *m*/*z* (ESI^+^) [found: 235.1, C_11_H_11_N_2_O_4_^+^ requires [M + H]^+^ 235.1]; R_f_: 0.55 (cyclohexane/ethyl acetate (6/4) (v/v)).

#### 3-Amino-2-hydroxy-*N*-methyl-*N*-(prop-2-yn-1-yl)benzamide (**17c**)

2-Hydroxy-*N*-methyl-3-nitro-*N*-(prop-2-yn-1-yl)benzamide (**16c,** 1.15 g, 4.86 mmol, 1.00 *eq*) and tin(II) chloride dihydrate (5.48 g, 24.3 mmol, 5.00 *eq*) were dissolved in methanol (25 mL) and heated under reflux for an hour. After cooling, the mixture was extracted with ethyl acetate (4 × 100 mL). After drying over sodium sulfate, filtration, and evaporation of the solvent, the title compound was obtained as a brown oil in quantitative yield that was used without further purification. LRMS *m*/*z* (ESI^+^) [found: 205.1, C_11_H_13_N_2_O_2_^+^ requires [M + H]^+^ 205.1].

#### 2-Hydroxy-3-[(4-methoxy-1,1-dioxido-1,2,5-thiadiazol-3-yl)amino]-*N,N*-dimethylbenzamide (**18a**)

3-Amino-2-hydroxy-*N,N*-dimethylbenzamide (**17a**, 0.1 g, 0.55 mmol, 1.0 *eq*) and 3,4-dimethoxy-1,2,5-thiadiazole-1,1-dioxide (99 mg, 0.55 mmol, 1.0 *eq*) were dissolved in methanol (10 mL) and stirred overnight. Filtration of the solid and drying under reduced pressure afforded the title compound as a green gum (130 mg, 72% yield) that was used without further purification. LRMS *m*/*z* (ESI^+^) [Found: 327.5, C_12_H_15_N_4_O_5_S^+^ requires [M + H]^+^ 327.3].

#### 2-Hydroxy-3-[(4-methoxy-1,1-dioxido-1,2,5-thiadiazol-3-yl)amino]-*N*-(prop-2-yn-1-yl)benzamide (**18b**)

3-Amino-2-hydroxy-*N*-(prop-2-yn-1-yl)benzamide (**17b**, 250 mg, 1.31 mmol, 1.00 *eq*) and 3,4-dimethoxy-1,2,5-thiadiazole-1,1-dioxide (234 mg, 1.31 mmol, 1.00 *eq*) were dissolved in methanol (10 mL) and stirred overnight. Filtration of the solid and drying under reduced pressure afforded the title compound as a green gum (200 mg, 45% yield) that was used without further purification. LRMS *m*/*z* (ESI^+^) [Found: 337.3, C_13_H_13_N_4_O_5_S^+^ requires [M + H]^+^ 337.3].

#### 2-Hydroxy-3-[(4-methoxy-1,1-dioxido-1,2,5-thiadiazol-3-yl)amino]-*N*-methyl-*N*-(prop-2-yn-1-yl)benzamide (**18c**)

3-Amino-2-hydroxy-*N*-methyl-*N*-(prop-2-yn-1-yl)benzamide (**17c**, 0.10 g, 0.49 mmol, 1.0 *eq*) and 3,4-dimethoxy-1,2,5-thiadiazole-1,1-dioxide (87 mg, 0.49 mmol, 1.0 *eq*) were dissolved in methanol (10 mL) and stirred overnight. Filtration of the solid and drying under reduced pressure afforded the title compound as a green gum (125 mg, 73% yield) that was used without further purification. LRMS *m*/*z* (ESI^+^) [Found: 351.5, C_14_H_15_N_4_O_5_S^+^ requires [M + H]^+^ 351.3].

#### (*R*)-3-[(4-{[2,2-Dimethyl-1-(5-methylfuran-2-yl)propyl]amino}-1,1-dioxido-1,2,5-thiadiazol-3-yl)amino]-2-hydroxy-*N*-methyl-N-(prop-2-yn-1-yl)benzamide (**20a**)

2-Hydroxy-3-[(4-methoxy-1,1-dioxido-1,2,5-thiadiazol-3-yl)amino]-*N*-methyl-*N*-(prop-2-yn-1-yl)benzamide (**18c**, 45 mg, 0.10 mmol, 1.0 *eq*) and (*R*)-2,2-dimethyl-1-(5-methylfuran-2-yl)propan-1-amine hydrochloride (**15c**, 31 mg, 0.10 mmol, 1.0 *eq*) were dissolved in methanol (10 mL). After the addition of *N,N*-diisopropylethylamine (46 μL, 0.20 mmol, 2.0 *eq*), the reaction was stirred for 3 days. Extraction between water and ethyl acetate (3 × 50 mL), drying over sodium sulfate, filtration, and evaporation of the solvent gave the crude product which was purified by preparative HPLC (acetonitrile/water (0.1% TFA): gradient 5–95%) to obtain the title compound as a white-brown amorphous solid (28 mg, 45%). ^1^H NMR (500 MHz, DMSO-*d*_6_, δ [ppm]): 10.49 (s, 1H), 9.94 (s, 1H), 8.85 (d, *J* = 9.3 Hz, 1H), 7.46 (dd, *J* = 8.0, 1.7 Hz, 1H), 7.13 (dd, *J* = 7.5, 1.6 Hz, 1H), 6.98 (t, *J* = 7.8 Hz, 1H), 6.32 (s, 1H), 6.07 (dd, *J* = 3.1, 1.2 Hz, 1H), 4.84 (d, *J* = 9.1 Hz, 1H), 4.16 (s, 2H), 3.26 (s, 1H), 2.92 (s, 3H), 2.29 (s, 3H), 1.02 (s, 9H); ^13^C NMR (126 MHz, DMSO-*d*_6_, δ [ppm]): 167.5, 156.0, 154.8, 151.2, 149.5, 147.5, 127.8, 126.8, 125.8, 124.5, 119.8, 109.5, 106.3, 79.1, 61.6, 40.1, 35.7, 26.5, 13.4; HRMS *m*/*z* (ESI^+^) [found: 486.1766, C_23_H_28_N_5_O_5_S^+^ requires [M + H]^+^ 486.1806]; HPLC retention time: 12.09 min, purity: 97.9%.

#### (*R*)-3-[(4-{[2,2-Dimethyl-1-(5-methylfuran-2-yl)propyl]amino}-1,1-dioxido-1,2,5-thiadiazol-3-yl)amino]-2-hydroxy-*N*-(prop-2-yn-1-yl)benzamide (**20b**)

2-Hydroxy-3-[(4-methoxy-1,1-dioxido-1,2,5-thiadiazol-3-yl)amino]-*N*-(prop-2-yn-1-yl)benzamide (**18b**, 100 mg, 0.300 mmol, 1.00 *eq*) and (*R*)-2,2-dimethyl-1-(5-methylfuran-2-yl)propan-1-amine hydrochloride (**15c**, 72 mg, 0.36 mmol, 1.2 *eq*) were dissolved in methanol (10 mL). After the addition of *N,N*-diisopropylethylamine (106 μL, 0.600 mmol, 2.00 *eq*), the reaction was stirred for 3 days. Extraction between water and ethyl acetate (3 × 50 mL), drying over sodium sulfate, filtration, and evaporation of the solvent gave the crude product which was purified by preparative HPLC (acetonitrile/water (0.1% TFA): gradient 5–95%) to obtain the title compound as a white-brown amorphous solid (93 mg, 66%). ^1^H NMR (600 MHz, methanol-*d*_4_, δ [ppm]): NH and OH signals were not detectable due to proton exchange, 8.18 (d, *J* = 8.1 Hz, 1H), 7.63 (d, *J* = 8.1 Hz, 1H), 6.97 (t, *J* = 8.1 Hz, 1H), 6.24 (d, *J* = 3.1 Hz, 1H), 6.00–5.98 (m, 1H), 4.98 (s, 1H), 4.18 (d, *J* = 2.6 Hz, 2H), 2.63 (t, *J* = 2.5 Hz, 1H), 2.30 (s, 3H), 1.08 (s, 9H); ^13^C NMR (151 MHz, methanol-*d*_4_, δ [ppm]): 171.2, 157.9, 154.9, 154.5, 153.2, 150.7, 129.3, 127.2, 125.5, 119.3, 115.8, 110.9, 107.2, 80.3, 72.3, 63.9, 37.0, 29.5, 27.2, 13.5; HRMS *m*/*z* (ESI^+^) [found: 494.1469, C_22_H_26_N_5_O_5_S^+^ requires [M + Na]^+^ 494.1576]; HPLC retention time: 12.74 min, purity: 99.1%.

#### (*R*)-2-Hydroxy-3-[(4-{[2-methyl-1-(5-methylfuran-2-yl)propyl]amino}-1,1-dioxido-1,2,5-thiadiazol-3-yl)amino]-*N*-(prop-2-yn-1-yl)benzamide (**20c**)

2-Hydroxy-3-[(4-methoxy-1,1-dioxido-1,2,5-thiadiazol-3-yl)amino]-*N*-(prop-2-yn-1-yl)benzamide (**18b**, 130 mg, 0.370 mmol, 1.00 *eq*) and (*R*)-2-methyl-1-(5-methylfuran-2-yl)propan-1-amine hydrochloride (**15b**, 57 mg, 0.37 mmol, 1.0 *eq*) were dissolved in methanol (10 mL). After the addition of *N,N*-diisopropylethylamine (132 μL, 0.740 mmol, 2.00 *eq* ), the reaction was stirred for 3 days. Extraction between water and ethyl acetate (3 × 50 mL), drying over sodium sulfate, filtration, and evaporation of the solvent gave the crude product which was purified by preparative HPLC (acetonitrile/water (0.1% TFA): gradient 5–95%) to obtain the title compound as a white-brown amorphous solid (38 mg, 22%). ^1^H NMR (600 MHz, DMSO-*d*_6_, δ [ppm]): 13.61 (s, 1H), 10.26 (s, 1H), 9.68–9.61 (m, 1H), 9.54–9.52 (m, 1H), 8.00–7.96 (m, 1H), 7.83–7.80 (m, 1H), 7.02 (t, *J* = 8.0 Hz, 1H), 6.37–6.32 (m, 1H), 6.08–6.06 (m, 1H), 5.04–4.62 (m, 1H), 4.14–4.12 (m, 2H), 3.21 (t, *J* = 2.5 Hz, 1H), 2.30–2.22 (m, 4H), 1.02 (d, *J* = 6.7 Hz, 2H), 1.02 (d, *J* = 6.7 Hz, 2H), 0.88 (d, *J* = 6.7 Hz, 2H); ^13^C NMR (151 MHz, DMSO-*d*_6_, δ [ppm]): 169.3, 169.2, 155.1, 154.8, 153.6, 153.5, 153.2, 153.2, 151.6, 151.4, 150.3, 149.7, 128.9, 128.7, 125.5, 125.4, 125.0, 124.9, 118.2, 114.5, 114.4, 109.3, 108.7, 106.5, 106.4, 80.2, 73.5, 58.7, 52.2, 34.3, 31.1, 28.4, 19.2, 19.2, 18.8, 13.4, 13.4, 13.3; HRMS *m*/*z* (ESI^+^) [found: 458.1493, C_21_H_24_N_5_O_5_S^+^ requires [M + H]^+^ 458.1420]; HPLC retention time: 12.59 min, purity: 97.9%

#### (*R*)-2-Hydroxy-3-[(4-{[1-(5-methylfuran-2-yl)propyl]amino}-1,1-dioxido-1,2,5-thiadiazol-3-yl)amino]-*N*-(prop-2-yn-1-yl)benzamide (**20d**)

2-Hydroxy-3-[(4-methoxy-1,1-dioxido-1,2,5-thiadiazol-3-yl)amino]-*N*-(prop-2-yn-1-yl)benzamide (**18b**, 130 mg, 0.370 mmol, 1.00 *eq*) and (*R*)-1-(5-methylfuran-2-yl)propan-1-amine hydrochloride (**15a**, 77 mg, 0.52 mmol, 1.4 *eq*) were dissolved in methanol (10 mL). After the addition of *N,N*-diisopropylethylamine (132 μL, 0.740 mmol, 2.00 *eq*), the reaction was stirred for 3 days. Extraction between water and ethyl acetate (3 × 50 mL), drying over sodium sulfate, filtration, and evaporation of the solvent gave the crude product which was purified by preparative HPLC (acetonitrile/water (0.1% TFA): gradient 5–95%) to obtain the title compound as a white-brown amorphous solid (37 mg, 22%). ^1^H NMR (600 MHz, DMSO-*d*_6_, δ [ppm]): 13.61 (s, 1H), 10.14 (s, 1H), 9.66 (d, *J* = 7.9 Hz, 1H), 9.53 (t, *J* = 5.5 Hz, 1H), 7.99 (dd, *J* = 8.1, 1.4 Hz, 1H), 7.81 (dd, *J* = 8.2, 1.5 Hz, 1H), 7.02 (t, *J* = 8.0 Hz, 1H), 6.37 (d, *J* = 3.1 Hz, 1H), 6.08 (d, *J* = 3.1 Hz, 1H), 4.60 (q, *J* = 7.5 Hz, 1H), 4.13 (dd, *J* = 5.5, 2.5 Hz, 2H), 3.21 (t, *J* = 2.5 Hz, 1H), 2.28 (s, 3H), 2.04–1.88 (m, 2H), 1.00–0.81 (m, 3H); ^13^C NMR (151 MHz, DMSO-*d*_6_, δ [ppm]): 169.3, 163.1, 154.8, 154.0, 153.5, 153.2, 151.6, 151.2, 150.4, 150.1, 128.7, 125.5, 124.9, 118.2, 114.4, 108.9, 108.3, 106.5, 106.4, 80.2, 73.5, 70.2, 53.9, 53.7, 28.4, 25.5, 25.0, 13.6, 13.3, 13.3, 10.6, 10.5; HRMS *m*/*z* (ESI^+^) [found: 444.1336, C_20_H_22_N_5_O_5_S^+^ requires [M + H]^+^ 444.1297]; HPLC retention time: 12.44 min, purity: 95.4%.

#### 2-Hydroxy-3-{[2-(neopentylamino)-3,4-dioxocyclobut-1-en-1-yl]amino}-*N*-(prop-2-yn-1-yl)benzamide (**21a**)

2-Hydroxy-3-[(2-methoxy-3,4-dioxocyclobut-1-en-1-yl)amino]-*N*-(prop-2-yn-1-yl)benzamide (**19**, 37 mg, 0.12 mmol, 1.0 *eq*) and neopentylamine (22 μL, 0.18 mmol, 1.5 *eq*) were dissolved in methanol (10 mL). After the addition of *N,N*-diisopropylethylamine (44 μL, 0.24 mmol, 2.0 *eq*), the reaction was stirred for 3 days. Extraction between water and ethyl acetate (3 × 50 mL), drying over sodium sulfate, filtration, and evaporation of the solvent gave the crude product which was purified by preparative HPLC (acetonitrile/water (0.1% TFA): gradient 5–95%) to obtain the title compound as a white-brown amorphous solid (20 mg, 45%). ^1^H NMR (500 MHz, DMSO-*d*_6_, δ [ppm]): 13.67 (s, 1H), 9.47 (t, *J* = 5.6 Hz, 1H), 9.40 (s, 1H), 8.36 (t, *J* = 6.8 Hz, 1H), 8.04–7.99 (m, 1H), 7.54 (d, *J* = 8.2 Hz, 1H), 6.90 (t, *J* = 8.1 Hz, 1H), 4.12 (dd, *J* = 5.6, 2.5 Hz, 2H), 3.43 (d, *J* = 6.7 Hz, 2H), 3.20 (t, *J* = 2.5 Hz, 1H), 0.93 (s, 9H); ^13^C NMR (126 MHz, DMSO-*d*_6_, δ [ppm]): 184.4, 180.1, 170.0, 169.8, 162.8, 150.9, 128.1, 123.4, 120.5, 118.3, 113.4, 80.3, 73.4, 54.8, 32.2, 28.4, 26.5; HRMS *m*/*z* (ESI^+^) [found: 356.1605, C_19_H_21_N_3_O_4_^+^ requires [M + H]^+^ 356.1612]; HPLC retention time: 12.62 min, purity: 95.0%.

#### 3-{[2-(Cyclobutylamino)-3,4-dioxocyclobut-1-en-1-yl]amino}-2-hydroxy-*N*-(prop-2-yn-1-yl)benzamide (**21b**)

2-Hydroxy-3-[(2-methoxy-3,4-dioxocyclobut-1-en-1-yl)amino]-*N*-(prop-2-yn-1-yl)benzamide (**19**, 30 mg, 0.075 mmol, 1.0 *eq*) and cyclobutylamine (11 μL, 0.075 mmol, 1.0 *eq*) were dissolved in methanol (10 mL). After the addition of *N,N*-diisopropylethylamine (36 μL, 0.15 mmol, 2.0 *eq*), the reaction was stirred for 3 days. Extraction between water and ethyl acetate (3 × 50 mL), drying over sodium sulfate, filtration, and evaporation of the solvent gave the crude product which was purified by preparative HPLC (acetonitrile/water (0.1% TFA): gradient 5–95%) to obtain the title compound as a white-brown amorphous solid (28 mg, 82%). ^1^H NMR (500 MHz, DMSO-*d*_6_, δ [ppm]): 13.63 (s, 1H), 9.46 (t, *J* = 5.6 Hz, 1H), 9.25 (s, 1H), 8.66 (d, *J* = 8.4 Hz, 1H), 8.00 (d, *J* = 8.0 Hz, 1H), 7.54 (dd, *J* = 8.2, 1.4 Hz, 1H), 6.90 (t, *J* = 8.0 Hz, 1H), 4.60–4.52 (m, 1H), 4.12–4.11 (m, 2H), 3.19 (t, *J* = 2.5 Hz, 1H), 2.37–2.30 (m, 2H), 2.10–1.97 (m, 2H), 1.77–1.59 (m, 2H); ^13^C NMR (126 MHz, DMSO-*d*_6_, δ [ppm]): 184.2, 180.1, 169.8, 168.3, 162.9, 150.8, 128.1, 123.3, 120.6, 118.3, 113.4, 80.3, 73.4, 48.7, 31.6, 28.4, 14.0; HRMS *m*/*z* (ESI^+^) [found: 340.1292, C_18_H_18_N_3_O_4_^+^ requires [M + H]^+^ 340.1296]; HPLC retention time: 12.12 min, purity: 96.8%.

#### 3-{[2-(Cyclopentylamino)-3,4-dioxocyclobut-1-en-1-yl]amino}-2-hydroxy-*N*-(prop-2-yn-1-yl)benzamide (**21c**)

2-Hydroxy-3-[(2-methoxy-3,4-dioxocyclobut-1-en-1-yl)amino]-*N*-(prop-2-yn-1-yl)benzamide (**19**, 30 mg, 0.075 mmol, 1.0 *eq*) and cyclopentylamine (13 μL, 0.075 mmol, 1.0 *eq*) were dissolved in methanol (10 mL). After the addition of *N,N*-diisopropylethylamine (36 μL, 0.15 mmol, 2.0 *eq*), the reaction was stirred for 3 days. Extraction between water and ethyl acetate (3 × 50 mL), drying over sodium sulfate, filtration, and evaporation of the solvent gave the crude product which was purified by preparative HPLC (acetonitrile/water (0.1% TFA): gradient 5–95%) to obtain the title compound as a white-brown amorphous solid (32 mg, 91%). ^1^H NMR (500 MHz, DMSO-*d*_6_, δ [ppm]): 13.65 (s, 1H), 9.46 (t, *J* = 5.6 Hz, 1H), 9.24 (s, 1H), 8.38 (d, *J* = 7.9 Hz, 1H), 8.01 (d, *J* = 8.0 Hz, 1H), 7.53 (dd, *J* = 8.2, 1.4 Hz, 1H), 6.90 (t, *J* = 8.1 Hz, 1H), 4.46–4.40 (m, 1H), 4.12–4.10 (m, 2H), 3.19 (t, *J* = 2.5 Hz, 1H), 2.00–1.92 (m, 2H), 1.78–1.50 (m, 6H); ^13^C NMR (126 MHz, DMSO-*d*_6_, δ [ppm]) 184.3, 179.9, 169.8, 168.8, 163.0, 150.8, 128.1, 123.3, 120.5, 118.3, 113.4, 80.3, 73.4, 55.4, 33.7, 28.4, 23.2; HRMS *m*/*z* (ESI^+^) [found: 354.1448, C_19_H_20_N_3_O_4_^+^ requires [M + H]^+^ 354.1455]; HPLC retention time: 12.31 min, purity: 97.3%.

#### 3-{[2-(Cyclohexylamino)-3,4-dioxocyclobut-1-en-1-yl]amino}-2-hydroxy-*N*-(prop-2-yn-1-yl)benzamide (**21d**)

2-Hydroxy-3-[(2-methoxy-3,4-dioxocyclobut-1-en-1-yl)amino]-*N*-(prop-2-yn-1-yl)benzamide (**19**, 30 mg, 0.075 mmol, 1.0 *eq*) and cyclohexylamine (15 μL, 0.075 mmol, 1.0 *eq*) were dissolved in methanol (10 mL). After the addition of *N,N*-diisopropylethylamine (36 μL, 0.15 mmol, 2.0 *eq*), the reaction was stirred for 3 days. Extraction between water and ethyl acetate (3 × 50 mL), drying over sodium sulfate, filtration, and evaporation of the solvent gave the crude product which was purified by preparative HPLC (acetonitrile/water (0.1% TFA): gradient 5–95%) to obtain the title compound as a white-brown amorphous solid (27 mg, 73%). ^1^H NMR (500 MHz, DMSO-*d*_6_, δ [ppm]): 13.64 (s, 1H), 9.46 (t, *J* = 5.6 Hz, 1H), 9.30 (s, 1H), 8.38 (d, *J* = 8.0 Hz, 1H), 8.00 (d, *J* = 8.0 Hz, 1H), 7.53 (dd, *J* = 8.1, 1.3 Hz, 1H), 6.90 (t, *J* = 8.1 Hz, 1H), 4.12–4.10 (m, 2H), 3.94–3.88 (m, 1H), 3.19 (t, *J* = 2.5 Hz, 1H), 1.96–1.91 (m, 2H), 1.75–1.69 (m, 2H), 1.59–1.54 (m, 1H), 1.40–1.15 (m, 5H); ^13^C NMR (126 MHz, DMSO-*d*_6_, δ [ppm]): 184.2, 179.9, 169.8, 168.6, 163.0, 150.9, 128.1, 123.4, 120.5, 118.3, 113.4, 80.3, 73.4, 52.4, 33.6, 28.4, 24.7, 23.9; HRMS *m*/*z* (ESI^+^) [found: 368.1605, C_20_H_22_N_3_O_4_^+^ requires [M + H]^+^ 368.1610]; HPLC retention time: 12.56 min, purity: 97.6%.

#### 3-{[2-(Adamantan-1-ylamino)-3,4-dioxocyclobut-1-en-1-yl]amino}-2-hydroxy-*N*-(prop-2-yn-1-yl)benzamide (**21e**)

2-Hydroxy-3-[(2-methoxy-3,4-dioxocyclobut-1-en-1-yl)amino]-*N*-(prop-2-yn-1-yl)benzamide (**19**, 30 mg, 0.075 mmol, 1.0 *eq*) and 1-adamantylamine (20 mg, 0.075 mmol, 1.0 *eq*) were dissolved in methanol (10 mL). After the addition of *N,N*-diisopropylethylamine (36 μL, 0.15 mmol, 2.0 *eq*), the reaction was stirred for 3 days. Extraction between water and ethyl acetate (3 × 50 mL), drying over sodium sulfate, filtration, and evaporation of the solvent gave the crude product which was purified by preparative HPLC (acetonitrile/water (0.1% TFA): gradient 5–95%) to obtain the title compound as a white-brown amorphous solid (24 mg, 57%). ^1^H NMR (500 MHz, DMSO-*d*_6_, δ [ppm]): 13.65 (s, 1H), 9.50–9.41 (m, 2H), 8.52 (s, 1H), 7.94 (dd, *J* = 8.0, 1.4 Hz, 1H), 7.55 (dd, *J* = 8.3, 1.4 Hz, 1H), 6.90 (t, *J* = 8.1 Hz, 1H), 4.12–4.11 (m, 2H), 3.19 (t, *J* = 2.5 Hz, 1H), 2.13–2.07 (m, 3H), 2.03–1.98 (m, 6H), 1.69–1.64 (m, 6H); ^13^C NMR (126 MHz, DMSO-*d*_6_, δ [ppm]): 182.9, 179.9, 169.8, 169.4, 164.0, 151.1, 127.9, 123.9, 120.8, 118.2, 113.4, 80.3, 73.4, 52.8, 42.4, 35.3, 29.0, 28.4; HRMS *m*/*z* (ESI^+^) [found: 420.1918, C_24_H_26_N_3_O_4_^+^ requires [M + H]^+^ 420.1918]; HPLC retention time: 13.41 min, purity: 95.9%.

#### 3-({2-[(Dicyclopropylmethyl)amino]-3,4-dioxocyclobut-1-en-1-yl}amino)-2-hydroxy-*N*-(prop-2-yn-1-yl)benzamide (**21f**)

2-Hydroxy-3-[(2-methoxy-3,4-dioxocyclobut-1-en-1-yl)amino]-*N*-(prop-2-yn-1-yl)benzamide (**19**, 30 mg, 0.075 mmol, 1.0 *eq*) and dicyclopropylmethanamine (13 μL, 0.075 mmol, 1.0 *eq*) were dissolved in methanol (10 mL). After the addition of *N,N*-diisopropylethylamine (36 μL, 0.15 mmol, 2.0 *eq*), the reaction was stirred for 3 days. Extraction between water and ethyl acetate (3 × 50 mL), drying over sodium sulfate, filtration, and evaporation of the solvent gave the crude product which was purified by preparative HPLC (acetonitrile/water (0.1% TFA): gradient 5–95%) to obtain the title compound as a white-brown amorphous solid (20 mg, 53%) ^1^H NMR (500 MHz, DMSO-*d*_6_, δ [ppm]): 13.64 (s, 1H), 9.47 (t, *J* = 5.5 Hz, 1H), 9.36 (s, 1H), 8.50 (d, *J* = 9.3 Hz, 1H), 8.01 (d, *J* = 7.9 Hz, 1H), 7.54 (d, *J* = 8.1 Hz, 1H), 6.90 (t, *J* = 8.0 Hz, 1H), 4.13–4.09 (m, 2H), 3.22–3.19 (m, 1H), 3.13 (q, *J* = 8.3 Hz, 1H), 1.13–1.04 (m, 2H), 0.59–0.53 (m, 2H), 0.47–0.44 (m, 2H), 0.37–0.34 (m, 4H); ^13^C NMR (126 MHz, DMSO-*d*_6_, δ [ppm]): 184.1, 179.7, 169.8, 168.6, 163.0, 150.9, 128.1, 123.5, 120.6, 118.3, 113.4, 80.3, 73.4, 60.9, 28.4, 15.7, 2.9, 1.9; HRMS *m*/*z* (ESI^+^) [found: 380.1605, C_21_H_22_N_3_O_4_^+^ requires [M + H]^+^ 380.1610; HPLC retention time: 12.47 min, purity: 97.4%.

#### 3-{[2-(Benzhydrylamino)-3,4-dioxocyclobut-1-en-1-yl)amino}-2-hydroxy-*N*-(prop-2-yn-1-yl)benzamide (**21g**)

2-Hydroxy-3-[(2-methoxy-3,4-dioxocyclobut-1-en-1-yl)amino]-*N*-(prop-2-yn-1-yl)benzamide (**19**, 30 mg, 0.075 mmol, 1.0 *eq*) and benzhydrylamine (24 mg, 0.075 mmol, 1.0 *eq*) were dissolved in methanol (10 mL). After the addition of *N,N*-diisopropylethylamine (36 μL, 0.15 mmol, 2.0 *eq*), the reaction was stirred for 3 days. Extraction between water and ethyl acetate (3 × 50 mL), drying over sodium sulfate, filtration, and evaporation of the solvent gave the crude product which was purified by preparative HPLC (acetonitrile/water (0.1% TFA): gradient 5–95%) to obtain the title compound as a white-brown amorphous solid (35 mg, 78%). ^1^H NMR (500 MHz, DMSO-*d*_6_, δ [ppm]): 13.66 (s, 1H), 9.50–9.42 (m, 2H), 9.15 (d, *J* = 8.8 Hz, 1H), 8.00 (d, *J* = 7.9 Hz, 1H), 7.55 (dd, *J* = 8.2, 1.4 Hz, 1H), 7.46–7.38 (m, 4H), 7.36–7.29 (m, 6H), 6.91 (t, *J* = 8.1 Hz, 1H), 6.52 (d, *J* = 8.8 Hz, 1H), 4.14–4.09 (m, 2H), 3.20 (t, *J* = 2.5 Hz, 1H); ^13^C NMR (126 MHz, DMSO-*d*_6_, δ [ppm]): 184.0, 180.6, 169.8, 168.4, 163.5, 150.9, 141.6, 128.8, 128.0, 127.6, 127.0, 123.4, 120.7, 118.3, 113.5, 80.3, 73.4, 60.6, 28.4; HRMS *m*/*z* (ESI^+^) [found: 452.1605, C_27_H_22_N_3_O_4_^+^ requires [M + H]^+^ 452.1611]; HPLC retention time: 12.87 min, purity: 97.8%.

#### 3-[(2-{[Di(pyridin-2-yl)methyl]amino}-3,4-dioxocyclobut-1-en-1-yl)amino]-2-hydroxy-*N*-(prop-2-yn-1-yl)benzamide (**21h**)

2-Hydroxy-3-[(2-methoxy-3,4-dioxocyclobut-1-en-1-yl)amino]-*N*-(prop-2-yn-1-yl)benzamide (**19**, 30 mg, 0.075 mmol, 1.0 *eq*) and bis(pyridine-2-yl)methanamine (25 mg, 0.075 mmol, 1.0 *eq*) were dissolved in methanol (10 mL). After the addition of *N,N*-diisopropylethylamine (36 μL, 0.15 mmol, 2.0 *eq*), the reaction was stirred for 3 days. Extraction between water and ethyl acetate (3 × 50 mL), drying over sodium sulfate, filtration, and evaporation of the solvent gave the crude product which was purified by preparative HPLC (acetonitrile/water (0.1% TFA): gradient 5–95%) to obtain the title compound as a white-brown amorphous solid (23 mg, 51%). ^1^H NMR (500 MHz, DMSO-*d*_6_, δ [ppm]): 13.62 (s, 1H), 9.88 (s, 1H), 9.66 (d, *J* = 9.0 Hz, 1H), 9.47 (t, *J* = 5.6 Hz, 1H), 8.60–8.56 (m, 2H), 7.94 (d, *J* = 8.0 Hz, 1H), 7.88 (td, *J* = 7.7, 1.8 Hz, 2H), 7.63–7.53 (m, 3H), 7.38–7.34 (m, 2H), 6.90 (t, *J* = 8.0 Hz, 1H), 6.72 (d, *J* = 9.0 Hz, 1H), 4.15–4.08 (m, 2H), 3.20 (t, *J* = 2.5 Hz, 1H); ^13^C NMR (126 MHz, DMSO-*d*_6_, δ [ppm]): 184.3, 180.8, 169.8, 168.4, 163.8, 158.8, 151.2, 148.9, 137.7, 128.0, 123.8, 123.0, 122.2, 120.8, 118.2, 113.5, 80.3, 73.4, 62.5, 28.4; HRMS *m*/*z* (ESI^+^) [found: 454.1510, C_25_H_320_N_5_O_4_^+^ requires [M + H]^+^ 454.1507]; HPLC retention time: 11.19 min, purity: 97.9%.

#### (*R*)-3-[(2-{[2,2-Dimethyl-1-(pyrimidin-5-yl)propyl]amino}-3,4-dioxocyclobut-1-en-1-yl)amino]-2-hydroxy-*N*-(prop-2-yn-1-yl)benzamide (**21i**)

2-Hydroxy-3-[(2-methoxy-3,4-dioxocyclobut-1-en-1-yl)amino]-*N*-(prop-2-yn-1-yl)benzamide (**19**, 40 mg, 0.10 mmol, 1.0 *eq*) and (*R*)-2,2-dimethyl-1-(pyrimidin-5-yl)propan-1-amine hydrochloride (**15e**, 27 mg, 0.10 mmol, 1.0 *eq*) were dissolved in methanol (10 mL). After the addition of *N,N*-diisopropylethylamine (71 μL, 0.30 mmol, 3.0 *eq*), the reaction was stirred for 3 days. Extraction between water and ethyl acetate (3 × 50 mL), drying over sodium sulfate, filtration, and evaporation of the solvent gave the crude product which was purified by preparative HPLC (acetonitrile/water (0.1% TFA): gradient 5–95%) to obtain the title compound as a white-brown amorphous solid (17 mg, 29%). ^1^H NMR (500 MHz, DMSO-*d*_6_, δ [ppm]): 13.77 (s, 1H), 9.51–9.46 (m, 2H), 9.14 (s, 1H), 8.77 (s, 2H), 8.68 (d, *J* = 9.3 Hz, 1H), 7.92 (d, *J* = 8.7 Hz, 1H), 7.56 (dd, *J* = 8.2, 1.4 Hz, 1H), 6.90 (t, *J* = 8.1 Hz, 1H), 5.13 (d, *J* = 9.3 Hz, 1H), 4.12 (dd, *J* = 5.6, 2.5 Hz, 2H), 3.20 (t, *J* = 2.5 Hz, 1H), 0.99 (s, 9H); ^13^C NMR (126 MHz, DMSO-*d*_6_, δ [ppm]): 183.9, 180.6, 169.8, 168.9, 163.8, 157.4, 156.0, 150.9, 133.5, 127.8, 123.7, 120.9, 118.3, 113.5, 80.3, 73.4, 62.6, 35.0, 28.4, 25.8; HRMS *m*/*z* (ESI^+^) [found: 434.1823, C_23_H_24_N_5_O_4_^+^ requires [M + H]^+^ 434.1824]; HPLC retention time: 11.93 min, purity: 96.8%.

#### (*R*)-3-[(2-{[1-(2,4-Dichlorothiazol-5-yl)-2,2-dimethylpropyl]amino}-3,4-dioxocyclobut-1-en-1-yl)amino]-2-hydroxy-*N*-(Prop-2-yn-1-yl)benzamide (**21j**)

2-Hydroxy-3-[(2-methoxy-3,4-dioxocyclobut-1-en-1-yl)amino]-*N*-(prop-2-yn-1-yl)benzamide (**19**, 40 mg, 0.10 mmol, 1.0 *eq*) and (*R*)-1-(2,4-dichlorothiazol-5-yl)-2,2-dimethylpropan-1-amine hydrochloride (**15f**, 37 mg, 0.10 mmol, 1.0 *eq*) were dissolved in methanol (10 mL). After the addition of *N,N*-diisopropylethylamine (71 μL, 0.30 mmol, 3.0 *eq*), the reaction was stirred for 3 days. Extraction between water and ethyl acetate (3 × 50 mL), drying over sodium sulfate, filtration, and evaporation of the solvent gave the crude product which was purified by preparative HPLC (acetonitrile/water (0.1% TFA): gradient 5–95%) to obtain the title compound as a white-brown amorphous solid (19 mg, 28%). ^1^H NMR (500 MHz, DMSO-*d*_6_, δ [ppm]): 13.75 (s, 1H), 9.49 (t, *J* = 5.6 Hz, 1H), 9.41 (s, 1H), 8.56 (d, *J* = 9.5 Hz, 1H), 7.93 (dd, *J* = 8.1, 1.3 Hz, 1H), 7.57 (dd, *J* = 8.2, 1.4 Hz, 1H), 6.91 (t, *J* = 8.1 Hz, 1H), 5.56 (d, *J* = 9.4 Hz, 1H), 4.12 (dd, *J* = 5.5, 2.5 Hz, 2H), 3.20 (t, *J* = 2.5 Hz, 1H), 1.05 (s, 9H); ^13^C NMR (126 MHz, DMSO-*d*_6_, δ [ppm]): 183.6, 180.7, 169.8, 168.4, 163.6, 151.0, 149.9, 134.3, 133.1, 127.7, 123.7, 120.9, 118.3, 113.5, 80.3, 73.4, 59.8, 36.4, 28.4, 25.7; HRMS *m*/*z* (ESI^+^) [found: 507.0669, C_22_H_21_Cl_2_N_4_O_4_S^+^ requires [M + H]^+^ 507.0655]; HPLC retention time: 13.18 min, 98.2%.

#### (*R*)-3-{[2-({1-[5-(4-Chlorophenyl)isoxazol-3-yl]-2,2-dimethylpropyl}amino)-3,4-dioxocyclobut-1-en-1-yl]amino}-2-hydroxy-*N*-(prop-2-yn-1-yl)benzamide (**21k**)

2-Hydroxy-3-[(2-methoxy-3,4-dioxocyclobut-1-en-1-yl)amino]-*N*-(prop-2-yn-1-yl)benzamide (**19**, 40 mg, 0.10 mmol, 1.0 *eq*) and (*R*)-1-[5-(4-chlorophenyl)isoxazol-3-yl]-2,2-dimethylpropan-1-amine hydrochloride (**15g**, 40 mg, 0.10 mmol, 1.0 *eq*) were dissolved in methanol (10 mL). After the addition of *N,N*-diisopropylethylamine (71 μL, 0.30 mmol, 3.0 *eq* ), the reaction was stirred for 3 days. Extraction between water and ethyl acetate (3 × 50 mL), drying over sodium sulfate, filtration, and evaporation of the solvent gave the crude product which was purified by preparative HPLC (acetonitrile/water (0.1% TFA): gradient 5–95%) to obtain the title compound as a white-brown amorphous solid (40 mg, 56%). ^1^H NMR (500 MHz, DMSO-*d*_6_, δ [ppm]): 13.70 (s, 1H), 9.62 (s, 1H), 9.48 (t, *J* = 5.6 Hz, 1H), 8.91 (d, *J* = 10.1 Hz, 1H), 7.97 (dd, *J* = 8.0, 1.3 Hz, 1H), 7.95–7.89 (m, 2H), 7.65–7.59 (m, 2H), 7.56 (dd, *J* = 8.2, 1.4 Hz, 1H), 7.12 (s, 1H), 6.90 (t, *J* = 8.0 Hz, 1H), 5.35 (d, *J* = 10.1 Hz, 1H), 4.12 (dd, *J* = 5.5, 2.5 Hz, 2H), 3.20 (t, *J* = 2.5 Hz, 1H), 1.05 (s, 9H); ^13^C NMR (126 MHz, DMSO-*d*_6_, δ [ppm]): 184.0, 180.4, 169.8, 168.7, 167.7, 163.3, 163.1, 151.0, 135.2, 129.3, 127.9, 127.5, 125.4, 123.7, 120.8, 118.3, 113.5, 101.0, 80.3, 73.4, 59.5, 35.4, 28.4, 25.9; HRMS *m*/*z* (ESI^+^) [found: 533.1601, C_28_H_26_ClN_4_O_5_^+^ requires [M + H]^+^ 533.1586]; HPLC retention time: 13.65 min, purity: 95.6%.

#### (*R*)-3-[(2-{[2,2-Dimethyl-1-(5-methylthiophen-2-yl)propyl]amino}-3,4-dioxocyclobut-1-en-1-yl)amino]-2-hydroxy-*N*-(prop-2-yn-1-yl)benzamide (**21l**)

2-Hydroxy-3-[(2-methoxy-3,4-dioxocyclobut-1-en-1-yl)amino]-*N*-(prop-2-yn-1-yl)benzamide (**19**, 40 mg, 0.10 mmol, 1.0 *eq*) and (*R*)-2,2-dimethyl-1-(5-methylthiophen-2-yl)propan-1-amine hydrochloride (**15d**, 29 mg, 0.10 mmol, 1.0 *eq*) were dissolved in methanol (10 mL). After the addition of *N,N*-diisopropylethylamine (71 μL, 0.30 mmol, 3.0 *eq*), the reaction was stirred for 3 days. Extraction between water and ethyl acetate (3 × 50 mL), drying over sodium sulfate, filtration, and evaporation of the solvent gave the crude product which was purified by preparative HPLC (acetonitrile/water (0.1% TFA): gradient 5–95%) to obtain the title compound as a white-brown amorphous solid (44 mg, 73%). ^1^H NMR (500 MHz, DMSO-*d*_6_, δ [ppm]): 13.72 (s, 1H), 9.52–9.45 (m, 2H), 8.62 (d, *J* = 10.0 Hz, 1H), 7.95 (d, *J* = 8.1 Hz, 1H), 7.55 (dd, *J* = 8.1, 1.4 Hz, 1H), 6.90 (t, *J* = 8.1 Hz, 1H), 6.78 (d, *J* = 3.5 Hz, 1H), 6.70 (dd, *J* = 3.4, 1.1 Hz, 1H), 5.32 (d, *J* = 10.0 Hz, 1H), 4.12 (dd, *J* = 5.5, 2.5 Hz, 2H), 3.20 (t, *J* = 2.5 Hz, 1H), 2.42 (d, *J* = 1.1 Hz, 3H), 1.01 (s, 9H); ^13^C NMR (126 MHz, DMSO-*d*_6_, δ [ppm]): 184.1, 180.2, 169.8, 168.5, 163.2, 151.0, 139.9, 138.5, 127.9, 126.3, 124.8, 123.7, 120.8, 118.3, 113.4, 80.3, 73.4, 62.8, 35.4, 28.4, 26.2, 14.8; HRMS *m*/*z* (ESI^+^) [found: 452.1648, C_24_H_26_N_3_O_4_S^+^ requires [M + H]^+^ 452.1639]; HPLC retention time: 13.22 min, purity: 97.9%.

#### (*R*)-3-[(2-{[2,2-Dimethyl-1-(5-methylfuran-2-yl)propyl]amino}-3,4-dioxocyclobut-1-en-1-yl)amino]-2-hydroxy-*N,N*-dimethylbenzamide (SLW132, **21m**)

2-Hydroxy-3-[(2-methoxy-3,4-dioxocyclobut-1-en-1-yl)amino]-*N*,*N*-dimethylbenzamide (**19b**, 60 mg, 0.2 mmol, 1.0 *eq*) and (*R*)-2,2-dimethyl-1-(5-methylfuran-2-yl)propan-1-amine hydrochloride (**15c**, 84 mg, 0.4 mmol, 2.0 *eq*) were dissolved in methanol (10 mL). After the addition of *N,N*-diisopropylethylamine (73 μL, 0.4 mmol, 2.0 *eq*), the reaction was stirred for 3 days. Extraction between water and ethyl acetate (3 × 50 mL), drying over sodium sulfate, filtration, and evaporation of the solvent gave the crude product that was purified by preparative HPLC (acetonitrile/water (0.1% TFA): gradient 5–95%) to obtain compound 19 as a white-brown amorphous solid (43 mg, 49%) ^1^H NMR (400 MHz, DMSO-*d*_6_, δ [ppm]): 9.97 (bs, 1H), 9.48 (s, 1H), 8.75 (d, *J* = 10.12 Hz, 1H), 7.76–7.72 (m, 1H), 6.91–6.85 (m, 2H), 6.19 (d, *J* = 3.12 Hz, 1H), 6.06–6.03 (m, 1H), 5.12 (d, *J* = 10.09 Hz, 1H), 2.95 (s, 6H), 2.28 (s, 3H), 0.98 (s, 9H); ^13^C NMR (151 MHz, DMSO-*d*_6_, δ [ppm]): 184.1, 180.3, 168.6, 168.3, 163.4, 151.0, 150.8, 143.5, 128.5, 124.4, 122.3, 121.2, 119.8, 108.5, 106.3, 60.2, 40.1, 35.7, 26.2, 13.4; HRMS *m*/*z* (ESI^+^) [found: 426.2028, C_23_H_28_N_3_O_5_^+^ requires [M + H]^+^ 426.1951]; LRMS *m*/*z* (ESI^+^) [found: 426.4, C_23_H_28_N_3_O_5_^+^ requires [M + H]^+^ 426.5]; HPLC retention time: 17.92 min, 98.0%.

### Molecular Modeling Studies

#### Structure Visualization

Structure visualization was done in Molecular Operating Environment (MOE, version 2022.02).^43^ The molecular surface was calculated at 4.5 Å around the *tert*-butyl group of Cmp2105 (**1**).

#### Protein Preparation

The protein structure (PDB ID: 6QZH) was prepared in MOE using default settings. This preparation included adding missing hydrogens and protonating titratable groups with Protonate3D. N- and C-termini, as well as sequence breaks, were capped, missing side chains were rebuilt by default. No energy minimization was performed. The prepared structure was processed with MakeReceptor (version 4.2.1.1)^44^ to make it compatible with other OpenEye tools. Hydrogens were left untouched, and the tautomer option was switched off. The box delimiting grids were created based on Cmp2105 (**1**) and default settings were applied to create the site shape potential.

#### Ligand Preparation

Protonation states were generated with fixpKa and tautomers (QUACPAC, version 2.2.2.0),^45^ and the most probable protonation state was chosen considering the proximity of Asp94 in the 6QZH crystal structure. Stereoisomers were generated with flipper (OMEGA, version 4.2.2.0)^46^ and ligand conformations were generated with the conformer_generator from CCDC (version 2023.3.0 CSD Portfolio 2023 release).^47^

#### Molecular Docking Calculations

Molecular docking calculations were performed with FRED (version 4.2.1.0).^48^ The top-scored pose for compound **3** (score −12.048) was minimized with SZYBKI (version 2.6.0.0)^49^ using steepest-descent optimization. Residual steric clashes were removed with MOE’s Energy Minimization module using only ligand atoms as input and the MMFF94x force field. Restraints were applied with a maximum allowed deviation of 1.5 Å from initial coordinates to discourage significant shifts from the docking-derived pose.

#### SZMAP Calculations

Atom charges and radii were assigned to the structure of the protein (PDB ID: 6QZH) using MakeReceptor. Desmethylated ligand **10** was prepared by removing methyl from the crystal structure of Cmp2105 (**1**). AM1BCC charges and Zap9 radii were assigned to ligands **1** and **10**. Grids were calculated separately for the methylated and desmethylated ligand, respectively, using default settings of SZMAP (version 1.6.6.0)^50^ and adding stabilization calculations. Ligand displacement grids were calculated separately using the grid_comp tool. Additionally, the difference between the grids for water–neutral probe free energies were calculated using grid_comp for both ligand **1** and **10**. Grids were visualized using VIDA (version 5.0.5).^51^ Free energy values for the water molecules were visualized with the WaterColor VIDA extension. Orientations of the predicted water molecules were sampled with the Water Orientation VIDA extension.

### Biological Evaluation

#### NanoBRET Assays

##### Cell Culture

HEK293T cells were cultured as previously described.^52^ In brief, HEK293T cells (gift from Chair of Physiology, Prof. Dr. Alzheimer, FAU Erlangen-Nürnberg) were grown on 10 cm culture dishes at 37 °C and 5% CO_2_. As growth medium, Dulbecco’s Modified Eagle Medium (DMEM)/F12 (Invitrogen) supplemented with 10% fetal bovine serum (FBS, Gibco Fetal Bovine Serum, qualified, Brazil), l-glutamine (final concentration: 2 mM; from Gibco l-glutamine 200 mM, 100×), penicillin (final concentration: 100 units/mL), and streptomycin (final concentration: 100 μg/mL; from Gibco Penicillin-Streptomycin 10,000 U/mL) was used. Cells were split every three to 4 days and regularly confirmed to be free of mycoplasma contamination using the luminescence-based MycoAlert Plus Kit (Lonza).

##### Transient Transfection Using Polyethylenimine

Transfection was performed as previously described.^52^ For transfection, we used a previously published procedure.^52^ In brief, HEK293T cells were plated onto culture dishes (Ø 10 cm or Ø 15 cm) and grown to a confluence of approximately 50% at 37 °C and 5% CO_2_. The growth medium was renewed 1 h before transfection. The transfection mix was prepared, as described in the following. Solution A (2.1–2.2% total DNA in Gibco phosphate buffered saline, pH 7.4 [5.5 μg in 250 μL, 10.5 μg in 500 μL]) and solution B (3% PEI (linear, 25 kDa, from Polysciences) solution prepared from a PEI stock solution (1 μg/μL) in PBS without MgCl_2_ and CaCl_2_) were mixed one to one, the resulting mixture was vortexed for 5 s and incubated for 30 min at room temperature. The preincubated transfection mix was added dropwise to the cells and cell cultivation was continued at 37 °C and 5% CO_2_.

##### Membrane Preparation

Membranes were prepared as previously reported.^52^ In brief, membranes from HEK293T cells transiently expressing the respective GPCR were prepared as follows. The medium of the transfected cells was refreshed after 24 h before cells were harvested 48 h post-transfection. The growth medium was removed, the cells were carefully washed with cold phosphate buffered saline (10 mL per Ø 15 cm dish). The cells were detached with 15 mL of ice-cold Tris-EDTA buffer (10 mM Tris, 0.5 mM EDTA, 5.4 mM KCl, 140 mM NaCl, pH 7.4) and subsequently centrifuged with 218 *g* for 8 min. The supernatant was removed, and the cells were resuspended in 10 mL Tris-EDTA buffer. The cells were lysed with an Ultraturrax (20,000 rpm) used five times for 5 s with a 25-s break on ice in between. The lysate was centrifuged at 50,830 *g* for 18 min at 4 °C. The supernatant was discarded, and the pellet was homogenized in membrane buffer (50 mM Tris, 1 mM EDTA, 5 mM MgCl_2_, 100 μg/mL bacitracin, 5 μg/mL soybean trypsin inhibitor, pH 7.4) with a glass-Teflon homogenizer. Aliquots of 250 μL were shock frozen in liquid nitrogen and directly stored at −80 °C. Finally, the protein concentration was determined using the Lowry method.^53^

##### cDNA Constructs

The CCR7-Nluc (CCR7_Nluc and CCR7_GSSG_Nluc) fusion constructs in pcDNA3.1 were generated as previously described,^52^ using the Gibson Assembly (New England Biolabs) method.^54^ Therefore, the sequences of the Nluc enzyme (pNLF1-C, Promega), (3xHA)-tagged CCR7 (CCR7, cdna.org, #CCR070TN00) were amplified by polymerase chain reaction and were directly fused in frame with different linker sequences (no linker and GSSG). DNA sequencing was performed to verify sequence integrity (Eurofins Genomics). Plasmids were cloned into *E. coli* DH5-alpha (New England Biolabs) and purified using a Maxiprep DNA purification kit (Invitrogen). The CCR7_GSSG_Nluc construct was already published.^10^

##### ELISA

ELISA-based experiments were performed as previously reported.^52^ For confirmation of CCR7_Nluc and CCR7_GSSG_Nluc expression, HEK293T cells were transfected with the plasmid encoding 3xHA-CCR7, 3xHA-tagged CCR7_Nluc or 3xHA-tagged CCR7_GSSG_Nluc construct using polyethylenimine in suspension. Therefore, HEK293T cells were detached from their culture plates and diluted to a density of 3 × 10^5^ cells/mL in growth medium. This cell suspension was mixed with the preformed transfection mix (PEI/DNA ratio 2.5:1) consisting of 1.2 μg of receptor cDNA plasmid and 1.2 μg of single stranded salmon sperm DNA (ssDNA, Sigma-Aldrich) in phosphate buffered saline (PBS) per 2.4 mL of cell suspension. Subsequently, cells were transferred to a 48-well plate (7.5 × 10^4^ cells/well), which was pretreated with poly-d-lysine (0.1 mg/mL, dissolved in water). Cells were incubated for 48 h at 37 °C and 5% CO_2_. On the day of the assay, the medium was removed, and cells were incubated with 200 μL/well of ROTIHistofix 4% fixation solution (Carl Roth) for 10 min at room temperature. Cells were washed once with 300 μL washing buffer for 2 min (150 mM NaCl, 25 mM Tris, pH 7.5) and blocked for 1 h using 800 μL blocking buffer (30 g/L skim milk powder in washing buffer). After removal of the blocking solution, 200 μL/well of anti-HA rabbit IgG antibody (Sigma-Aldrich, catalog # H6908, 1:4,000 in blocking solution) were added. After 60 min of incubation, wells were washed twice for 2 min (300 μL/well) and blocked again for 1 h at room temperature, before 200 μL/well antirabbit IgG-HRP antibody (Invitrogen by Thermo Fisher Scientific, catalog # G-21234, 1:1,000 in blocking solution) was added. After incubation for 1 h, cells were washed three times for 2 min (300 μL/well), before the substrate reaction was initiated by the addition of substrate buffer (6 mM o-phenylenediamine in 35 mM citric acid, 66 mM Na_2_HPO_4_, pH 5.0). After 15 min incubation in the dark, the reactions were terminated by addition of 1 M H_2_SO_4_ (200 μL/well). For each well, 2 × 150 μL of the resulting mixture were transferred to a clear, flat bottom 96-well plate and absorption was measured at 492 nm in a microplate reader. The measured absorbance values were baseline-corrected using cells transfected with a nontagged muscarinic receptor (M3R, cdna.org) as negative control. These baseline-corrected values were normalized to 3xHA-CCR7 expression.

##### Emission and Excitation Spectra of the Fluorescent Ligand

For the detection of the emission and excitation spectra of the fluorescent ligand, we referred to a previously published procedure.^10,11^ For the excitation spectrum, the fluorescent ligand was diluted to 500 μM in aqueous solution and 25 μL of this solutions were pipetted into a 384-well plate. Then, the excitation spectrum was measured with a CLARIOstar microplate reader. The emission spectrum of the fluorescent ligand (1 mM in DMSO) was recorded using a CLARIOstar (BMG Labtech, Ortenberg, Germany) microplate reader and 480 nm as excitation wavelength.

##### Emission Spectra of Nluc-Labeled CCR7 Proteins

Furimazine (Promega, Mannheim, Germany 1:2,000) was added to the membrane preparations (5 μg protein/well). After 5 min incubation in the dark, the emission spectra were measured ranging from 350 to 700 nm using a CLARIOstar (BMG Labtech, Ortenberg, Germany) microplate reader. The emission spectra of CCR7_GSSG_Nluc has already been reported by Huber et al.^10,11^

##### NanoBRET Binding Assays: Membrane-Based NanoBRET Saturation Assay

For the establishment of our NanoBRET binding assay, we referred to recently published protocols.^10,11^ The fluorescent ligand Mz437 (**4**) was dissolved in DMSO (1 mM) and further diluted to varying concentrations in assay buffer (50 mM Na_2_HPO_4_, 50 mM KH_2_PO_4_, pH 7.4, 1 mg/mL saponin, 5% FBS) and 5 μL of these dilutions were pipetted to a 384-well plate. To determine total binding, 5 μL of assay buffer were added to the corresponding wells, while 5 μL of a solution of the nonfluorescent SLW131 (**10**, final assay concentration: 15 μM) in assay buffer were used to determine nonspecific binding. Then, 20 μL of the membrane preparation (CCR7_GSSG_Nluc) diluted in assay buffer (3 μg total protein/well) were added and the plates were incubated for 90 min at 37 °C. Subsequently, 5 μL of a furimazine solution (Promega, Mannheim, Germany, final assay dilution: 1:5,000) were added to each well (final assay volume: 35 μL) before measuring luminescence with a CLARIOstar microplate reader using 620/10 nm and 475/30 nm emission filters after 5 min of incubation in the dark. Bioluminescence resonance energy transfer (BRET) was determined as the ratio of acceptor fluorescence and donor luminescence. The algorithms for one-site saturation binding from PRISM10.2.1 (GraphPad, USA) were utilized to analyze total, nonspecific and specific binding. Specific binding signals were calculated as a difference of total and nonspecific binding. If required, netBRET values were calculated as the difference between total BRET values and the values obtained in the absence of a fluorescent ligand.

##### Membrane-Based NanoBRET Competition Assay

The fluorescent ligand Mz437 (**4**) was dissolved in assay buffer and 5 μL of this solution (final assay concentration: 500 nM) were pipetted to a 384-well plate, followed by the addition of 5 μL of varying dilutions of the competing ligand dissolved in assay buffer. Then, 20 μL of the membrane preparations (CCR7_GSSG_Nluc diluted in assay buffer, 2.5 μg total protein/well) were added and the plates were incubated for 90 min at 37 °C. Subsequently, 5 μL of a furimazine solution (final assay dilution: 1:5,000 in assay buffer) were added to each well (final assay volume: 35 μL). Plates were read on a CLARIOstar microplate reader using 620/10 nm and 475/30 nm emission filters after 5 min of incubation in the dark. To determine the inhibition constants (*K*_i_) of the nonlabeled ligands, data were analyzed using the one site-fit *K*_i_ equation in PRISM10.2.1 (GraphPad, USA). For compounds that showed more than 50% competition at the highest concentration tested, we manually set a constraint for the curve fitting to approach the value detected for nonspecific binding (0% specific BRET). For compounds that showed less than 50% competition at the highest competitor concentration tested, only the values for percentual inhibition of tracer binding at a given concentration are provided.

##### Membrane-Based NanoBRET Association Kinetic Assay

5 μL of a solution of the fluorescent ligand Mz437 (**4**) diluted to varying concentrations (final assay concentrations: 100–1000 nM) in assay buffer and 5 μL of assay buffer were transferred to a 384-well plate. For the determination of nonspecific binding, we added a solution of a nonlabeled competitor SLW131 (**10**) dissolved in assay buffer (final assay concentration: 10 μM) instead. After the addition of 5 μL of a furimazine solution (final assay dilution: 1:630 in assay buffer), plates were incubated for 3 min in the dark at ambient temperature. Subsequently, 20 μL of the membrane preparations (CCR7_GSSG_Nluc, 4 μg total protein/well) were added (final assay volume: 35 μL). BRET ratios were measured with a CLARIOstar microplate reader using 620/10 nm and 475/30 nm emission filters over time at ambient temperature. The obtained data were analyzed using the association kinetics (one ligand concentration) algorithm in PRISM10.2.1 (GraphPad, USA) to determine association kinetics using a predetermined *k*_off_ as a constraint.

##### Membrane-Based NanoBRET Dissociation Kinetic Assay

5 μL of a solution of the fluorescent ligand Mz437 (**4**) diluted to varying concentrations (final assay concentrations: 250–1000 nM) in assay buffer and 5 μL of assay buffer were transferred to a 384-well plate. In order to determine nonspecific binding, we added a solution of a nonlabeled competitor SLW131 (**10**) dissolved in assay buffer (final assay concentration: 10 μM) instead of the 5 μL of assay buffer. Then, 20 μL of the membrane preparation (4 μg total protein/well) were added, and plates were incubated for 1.5–2 h at ambient temperature in the dark. Subsequently, 5 μL of a furimazine solution (final assay dilution: 1:630 in assay buffer) were added. Plates were incubated for further 5 min in the dark at ambient temperature. Thereafter, 1 μL of a solution of the unlabeled competitor SLW131 (**10**) dissolved in assay buffer (final assay concentration: 10 μM) was added. For control experiments, we added 1 μL of assay buffer instead of the competitor solution. BRET ratios were measured with a CLARIOstar microplate reader using 620/10 nm and 475/30 nm emission filters over time at ambient temperature. Specific BRET ratios were calculated as a difference of total and nonspecific binding. The obtained data were analyzed using the dissociation—one phase exponential decay algorithm in PRISM10.2.1 (GraphPad, USA) to determine dissociation kinetics.

##### Live Cell NanoBRET

HEK293T cells were transfected with 5.5 μg of the plasmid (CCR7_GSSG_Nluc) using polyethylenimine (PEI; 7.5 μg) as transfection reagent. After 24 h at 37 °C and 5% CO_2_, the cells were detached with DMEM and transferred to a white F-bottom assay 384-well plate [10,000 cells/well], which was coated with poly-d-lysine (0.1 mg/mL, dissolved in water), and incubated for further 24 h at 37 °C and 5% CO_2_. Subsequently, cells were washed with phosphate-buffered saline (Gibco DPBS, with CaCl_2_ and MgCl_2_). Assay medium (Gibco DMEM/F-12, 15 mM HEPES, no phenol red supplemented with 5% FBS) was added and cells were incubated at 37 °C for 30 min. Then, 5 μL of a solution containing the fluorescent ligand Mz437 (**4**), diluted in assay medium at varying concentrations, were added in case of saturation binding experiments. To determine nonspecific binding, 5 μL of a solution of the unlabeled competitor SLW131 (**10**) dissolved in assay medium (final assay concentration: 10 μM) were added. For competition binding experiments, 5 μL of a solution of the fluorescent ligand (**4**) diluted in assay medium (final assay concentration: 500 nM) and 5 μL of a solution of the potential competitor (diluted from 10 mM DMSO-stock solutions with assay medium) at varying concentrations were added to the corresponding wells. After 90 min of incubation at 37 °C, 5 μL of a furimazine solution (final assay dilution: 1:2,500–1:2,700, diluted with assay medium) were added. At the 96-well plates the double volume was added. After a further incubation of 5 min in the dark at 37 °C, BRET ratios were measured with a CLARIOstar microplate reader using 620/10 nm and 475/30 nm emission filters. Total, nonspecific and specific binding, which was calculated as a difference of total and nonspecific binding, were analyzed using the algorithms for one-site saturation binding in PRISM10.2.1 (GraphPad, USA). To determine the inhibition constants (*K*_i_) of the potential competitors, data were normalized to total and nonspecific binding and analyzed using the one site-fit *K*_i_ equation in PRISM10.2.1 (GraphPad, USA).

### Cell Culture

HEK293T cells (obtained from the American Type Culture Collection) were grown in Dulbecco’s Modified Eagle Medium (DMEM, Thermo Fisher Scientific) supplemented with 10% fetal bovine serum (FBS, PANbiotech), 100 units/mL penicillin and 100 μg/mL streptomycin at 37 °C and 5% CO_2_. For culture of stable hCCR7-HEK293 DMEM was supplemented with G418 (500 μg/mL) (InvivoGen). For generation of single clones, cells were detached and seeded into a 96-well plate at a density of 1 cell per well in complete culture media. Cells that grew to confluency were detached and expanded stepwise.

### β-Arrestin2 NanoBiT Assay

HEK293T cells were transfected with 500 ng hCCR7-smBiT, 50 ng LgBiT-β-arrestin2 mutant (R393E, R395E) (kindly provided by Asuka Inoue) and 4450 ng empty pcDNA3.1 per 3 × 10^6^ cells using PEI. At 28 h post-transfection arrestin recruitment was measured using NanoBiT technology. Briefly, cells were counted, resuspended in HBSS with 20 mM HEPES and transferred to a white 96-well plate with 60,000 cells per well. After preincubation of cells with antagonists or vehicle (final DMSO concentration 0.1%) for 30 min, cells were loaded with Nano-Glo Nano luciferase substrate (Promega, final dilution 1:1000) for 3 min, baseline luminescence was recorded and cells were stimulated with the indicated CCL19 concentration. Luminescence signals were measured using a PHERAstar FSX microplate reader (BMG Labtech). Luminescence changes were baseline- and buffer-corrected; concentration-inhibition-curves were derived from the maximum luminescence responses and expressed as percentage of the indicated concentration of CCL19.

### Real-Time BRET-Based G Protein Activation Assay

HEK293T cells were transfected in suspension with 1500 ng hCCR7, 800 ng Gαo, 400 ng Venus(156–239)-Gβ1, 400 ng Venus(1–155)-Gγ2 and 400 ng masGRK3ct-Nluc (BRET sensor plasmids were kindly provided by Kirill A. Martemyanov) using polyethylenimine (PEI, 25 kDa, linear, Polysciences, 1 mg/mL, DNA to PEI solution ratio 1:3). Empty pcDNA3.1 vector was used to adjust the total amount of DNA to 5000 ng per transfection of 3 × 10^6^ cells in a 10 cm dish (typical transfection efficiency ∼50–70%). At 28 h post-transfection, BRET was measured according to previously published protocol.^55^ Briefly, cells were counted, resuspended in Hank’s Balanced Salt Solution (HBSS) supplemented with 20 mM HEPES and transferred to a white 96-well plate with 80,000 cells per well. HEK293T cells were preincubated with antagonists or vehicle (final DMSO concentration 0.1%) for 30 min. Afterward Nano-Glo nanoluciferase substrate (Promega, final dilution 1:1000) was added for 3 min before recording baseline BRET and stimulating cells with the indicated CCL19 concentration. BRET was recorded with a PHERAstar FSX microplate reader (BMG Labtech) and calculated as ratio of the fluorescence emitted by Venus-Gβγ (535 ± 30 nm) and the luminescence of GRK3-Nluc (475 ± 30 nm). The difference between the BRET ratio before and after agonist stimulation was calculated and buffer-corrected. Concentration-inhibition-curves were derived from the maximum BRET signals and expressed as percentage of the indicated concentration of CCL19.

### Label-Free Whole Cell Biosensing Based on Detection of Dynamic Mass Redistribution (DMR)

For DMR experiments, stable hCCR7-HEK293 cells were seeded at a density of 18,000 cells per well into fibronectin-coated 384-well biosensor plates. Murine BMDCs were plated on 100 mm Petri dishes (nonadhesive plastic; Fisher; cat.nr: 10470613) at a density of 2 × 10^6^ cells/plate in complete medium (RPMI 1640 supplemented with 10% fetal calf serum, 2 mM l-glutamine, 100 U/mL penicillin, 100 μg/mL streptomycin, 50 μM β-mercaptoethanol; all purchased from Thermo Fisher Scientific) containing 10% GM-CSF (supernatant from hybridoma culture). DC differentiation was induced with 200 ng/mL LPS from *E. coli* 0127:B8 (Sigma-Aldrich) for 24–48 h at 37 °C and 5% CO_2_. The supernatant was then collected to harvest the mature nonadherent BMDCs, cells were washed with assay buffer (HBSS with 20 mM HEPES) and seeded at a density of 80,000 cells per well on 384-well fibronectin-coated biosensor plates. Cells were incubated for 60 min at 37 °C in the EPIC reader (EnSight Multimode Plate Reader, PerkinElmer, MA, US), followed by a 3 min baseline read. Thereafter, SLW131 (**10**) and SLW132 (**21m**) were added using the Selma semiautomatic liquid handling system (Analytik Jena AG, Jena, DE) for another 60 min. After a second 3 min baseline read, CCL19 was added and wavelength shifts over time were recorded for 3600 s. Real-time DMR recordings were buffer-corrected and are presented as wavelength shift over time; concentration-effect-curves were derived from the peak wavelength shifts, concentration-inhibition-relationships are expressed as percentage of the indicated concentration of CCL19.

### Fluorescence Microscopy

Stably transfected hCCR7-HEK293 cells were seeded onto poly-d-lysine (PDL)-coated 8-well μ-slides (Ibidi) at a density of 1 × 10^5^ cells per well and cultured overnight at 37 °C and 5% CO_2_. For immunostaining, the cells were first fixed with 4% paraformaldehyde solution, washed with PBS, and then blocked for 60 min with 10% goat serum and 1% fatty acid-free bovine serum albumin (BSA) in PBS at 37 °C. Cells were then incubated with the primary antibody, anti-CCR7 mouse IgG (R and D Systems, catalog # 150503, 1:100 in blocking solution) for 60 min at 37 °C. Following intense washing with PBS, the secondary antibody, goat antimouse IgG (H + L) conjugated to FITC (Sigma-Aldrich, catalog # AP124F, 1:500 in blocking solution) was added for 60 min at 37 °C. After a second round of intense washing with PBS, cells were counterstained with 4′,6-diamidin-2-phenylindole (DAPI) solution (0.1 μg/mL) for 15 min in the dark at room temperature. The following day, cells were treated with either cmp2105 (**1**), SLW131 (**10**), or SLW132 (**21m**) (at a final concentration of 10 μM each, or vehicle solution (OptiMEM reduced serum medium (Gibco) with 0.1% DMSO) for 60 min at 37 °C. Thereafter, the fluorescent CCR7 probe Mz437 (**4**) (at a final concentration of 500 nM) was added for 60 min at 37 °C and fluorescence images were acquired. Microscopy was performed using an AxioObserver.Z1 microscope (Carl Zeiss, Jena, Germany) equipped with an ApoTome imaging system and a Heating unit XL S, utilizing a Plan-Apochromat ×63/1.40 Oil objective (filter set 43 (red), 38 (green), 49 (blue)). Image processing and line scan analysis were conducted using Zen Blue Imaging software. Fluorescence intensity values were normalized to the vehicle control.

### CD4^+^ T Cell Transwell Migration Assay

For the determination of CCR7 expression by naive CD4^+^ T cells, single-cell suspensions were prepared from skin-draining lymph nodes of wild-type C57BL/6 mice (Charles River Laboratories) and filtered through a 70 μm cell strainer. Cells were washed using 1× PBS and stained in 96-well V-bottom plates. Unspecific binding was blocked by preincubating cells with 2% normal rat serum (STEMCELL Technologies) and anti-CD16/CD32 in 1× PBS. The following antibodies were used for surface staining at 37 °C for 30 min in flow cytometry buffer (2% FBS, 2 mM EDTA and 0.01% NaN_3_ in 1× PBS: CD4-BV510 (clone RM4-5, BioLegend, Cat# 100559), CD8a-BUV737 (clone 53–6.7, BD, Cat# 612759), CD19-BUV737 (clone 1D3, BD, Cat# 612781), CD44-BV650 (clone IM7, BD, Cat# 740455), and CD197(CCR7)-APC (clone 4B12, eBioscience, Cat# 17-1971-81). Cells were subsequently washed and stained with the Fixable Viability Dye eFluor 780 (eBioscience, Cat# 65-0865-14) to exclude dead cells. Samples were acquired on a BD LSRFortessa. Data were analyzed with FlowJo software (Treestar) and compensated with OneComp eBeads Compensation Beads (Thermo Fisher Scientific, #01-1234-42).

For the transwell migration assay, wild-type C57BL/6 mice were used to isolate naive CD4^+^ T cells from their skin-draining lymph nodes and spleens using the EasySep Mouse Naive CD4^+^ T Cell Isolation Kit (Stemcell). The purity of naive CD4^+^ T cells was consistently over 95%. The T cells (1 × 10^6^ cells/ml) were incubated with 10 μM of compound Cmp2105 (**1**), SLW131 (**10**), SLW132 (**21m**), and a DMSO control in chemotaxis buffer (RPMI + 0,5% BSA + 10 mM HEPES + 100 U/ml penicillin/streptomycin + 1 mM sodium pyruvate + 1× nonessential amino acid solution + 50 μM β-mercaptoethanol) for 30 min. In the lower compartment of the transwell plate (Corning 3421), 600 μL of chemotaxis buffer were added, with and without 100 ng/mL CCL19 or 100 ng/mL CXCL12. A total of 100 μL of the cell suspension was then transferred directly into the upper compartment of a transwell insert. After a 2.5-h incubation period, live migrated CD4^+^ T cells were quantified using flow cytometry on a BD LSRFortessa with the aid of counting beads.^56^ The percentage of net migration was calculated by subtracting the number of cells that migrated in the absence of chemokines from the number of cells that migrated to CCL19 or CXCL12, then dividing the result by the number of cells in the input control, and multiplying by 100. The input control consisted of 100 μL of cells added directly to the lower compartment.

### Dendritic Cell (DC) Culture

Cultures were started from freshly isolated bone marrow of 8–12-week-old mice with C57BL/6J background. DC differentiation was induced by plating 2 × 10^6^ cells in 10 mL complete medium (Roswell Park Memorial Institute (RPMI) 1640 supplemented with 10% Fetal Calf Serum, 2 mM l-Glutamine, 100 U/mL Penicillin, 100 μg/mL Streptomycin, 50 μM β-Mercaptoethanol) (all purchased from Thermo Fischer) containing 10% Granulocyte-Monocyte Colony Stimulating Factor (GM-CSF, supernatant from hybridoma culture). Cells were fed on day 3 and 6 with complete medium supplemented with 20% GM-CSF. To induce maturation, cells were stimulated overnight with 200 ng/mL lipopolysaccharide (LPS) from *E. coli* 0127:B8 (Sigma) and used for experiments on day 8–9 (mature DCs). DC differentiation and maturation was confirmed by flow cytometric assessment of surface markers such as MHCII, CD11c, CD86 and CCR7. For 3D collagen migration experiments, mature cells were treated with 10 μM cmp2105 (**1**), SLW131 (**10**), SLW132 (**21m**) or DMSO (ctrl) for 1 h, casted into migration chambers and recorded by video-microscopy.

### Flow Cytometry

Before staining, 1–2 × 10^6^ cells were incubated for 15 min at 4 °C with blocking buffer (1 × PBS, 1% BSA, 2 mM EDTA) containing 5 mg/mL anti-CD16/CD32 antibody (2.4G2; BD Biosciences). For cell surface staining, cells were incubated for 30 min at 4 °C with conjugated monoclonal antibodies diluted in blocking buffer. The following antibodies were used: mouse anti-mouse CCR7-PE (4B12, 1:300), rat anti-mouse I-A/I-E-eFluor450 (M5/114.15.2, 1:800), hamster anti-mouse CD11c-APC (N418, 1:300), anti-mouse CD86 PE (GL1, 1:500). For live dead staining the Live/Dead Fixable Dead Cell Stain Kit (1:1000, Invitrogen) or DRAQ7 (1:1000, Biolegend) was used. Flow cytometry was performed on a LSR flow cytometer (BD Biosciences). Data analysis was carried out using FlowJo X 10.0.7r2.

### *In Vitro* 3D Collagen Migration Assay

For 3D *in vitro* migration, 2 × 10^5^ bone marrow-derived DCs were pretreated with 10 μM Cmp2105 (**1**), SLW131 (**10**), SLW132 (**21m**) or DMSO (ctrl) for 1 h and subsequently suspended in a medium-collagen I mixture (PureCol bovine collagen, (INAMED) in 1× minimum essential medium eagle (MEM, Invitrogen) and 0.4% sodium bicarbonate (Sigma) at a volume ratio of 1:2 yielding in a final collagen concentration of 1.73 mg/mL. Collagen gel mixtures were casted into custom-made migration chambers as previously described^52,57^ and incubated for 45 min at 37 °C to allow polymerization of the gel. CCL19 was suspended in full medium to a final concentration 100 nM and placed on top of the gel. To prevent drying-out of the gels, migration chambers were sealed with Paraffin wax (Sigma-Aldrich). Gels that failed to polymerize were excluded from the analysis.

Image acquisition was performed with a Nikon Eclipse widefield microscope and a C-Apochromat 10×/0.3 PH1 air objective. Images were acquired in 120 s intervals for 2 h at 37 °C, 5% CO_2_. 50 cells were tracked manually, using the “Manual tracking Plug-in” for ImageJ. The ImageJ Chemotaxis tool was used to determine average (frame-to-frame) speed and persistence distance in gradient direction/total distance). Additionally, automized y-displacement over time was measured using an in-house generated MATLAB script as recently described.^52,57^

## Supporting Information Available

Supplementary figures, schemes, and tables, NMR spectra, and HPLC traces (PDF)
Coordinate information on docked complexes (PDB)
(PDB)
(PDB)
Molecular strings formula (CSV)

## Supplementary Material

jm4c02102_si_001.pdf

jm4c02102_si_002.pdb

jm4c02102_si_003.pdb

jm4c02102_si_004.pdb

## Abbreviations

BMDC: bone-marrow derived dendritic cell
BRET: bioluminescence-resonance energy transfer
BSA: bovine serum albumin
CCL19: C–C chemokine ligand 19
CCR1: C–C chemokine receptor 1
CCR2: C–C chemokine receptor 2
CCR6: C–C chemokine receptor 6
CCR7: C–C chemokine receptor 7
CCR9: C–C chemokine receptor 9
CD4: cluster of differentiation 4
CDCl_3_: deuterated chloroform
CXCL12: C-X-C chemokine ligand 12
CXCR1: C-X-C chemokine receptor 1
CXCR2: C-X-C chemokine receptor 2
CXCR4: C-X-C chemokine receptor 4
DC: dendritic cell
DCM: dichloromethane
DIPEA: *N,N-*diisopropylethylamine
DMF: *N*,*N*-dimethylformamide
DMR: dynamic mass redistribution
DMSO: dimethyl sulfoxide
dr: diastereomeric ratio
EC: effective concentration
EDTA: ethylenediaminetetraacetic acid
ELISA: enzyme-linked immunosorbent assay
eq: equivalent
ESI: electrospray ionization
Et_2_O: diethyl ether
GPCR: G protein-coupled receptor
HEK293: human embryonic kidney cells
HPLC: high-performance liquid chromatography
HR-MS: high-resolution mass spectrometry
IABS: intracellular allosteric binding site
IC: inhibitory concentration
*K*_i_: inhibitory constant
LR-MS: low-resolution mass spectrometry
MEM: minimum essential medium eagle
MeOH: methanol
MOE: molecular open environment
NMR: nuclear magnetic resonance
PBS: phosphate buffered saline
ppm: parts per million
PyBroP: bromo-tris-pyrrolidino-phosphonium hexafluorophosphate
SAR: structure–activity relationship
SEM: standard error of mean
SnCl_2_: tin(II)chloride
TAMRA: 5-carboxytetramethylrhodamine
TBTA: tris[(1-benzyl-1*H*-1,2,3-triazol-4-yl)methyl]amin)
TFA: trifluoroacetic acid
THF: tetrahydrofuran
Tris: tris(hydroxymethyl)aminomethane
