# Supplementary material for: A Fluorescent Probe Enables the Discovery of Improved Antagonists Targeting the Intracellular Allosteric Site of the Chemokine Receptor CCR7
Source: J Med Chem. 2025 Feb 12;68(4):4308–33. doi: 10.1021/acs.jmedchem.4c02102 (PMC11873976; doi:10.1021/acs.jmedchem.4c02102)
Supplement: Supplementary file 1 — jm4c02102_si_001.pdf [file jm4c02102_si_001.pdf]

Supporting Information

A fluorescent probe enables the discovery of improved antagonists targeting the intracellular allosteric site of the chemokine receptor CCR7

Silas L. Wurnig,[1,‡] Max E. Huber,[2,‡] Corinna Weiler,[3,‡] Hanna Baltrukevich,[4] Nicole Merten,[3] Isabel Stötzel,[5] Yinshui Chang,[6] Teresa Steffen,[6] René H. L. Klammer,[1] Dirk Baumjohann,[6] Eva Kiermaier,[5] Peter Kolb,[4] Evi Kostenis,[3,] Matthias Schiedel,[2,7,]* Finn K. Hansen[1,]**

[1]Department of Pharmaceutical & Cell Biological Chemistry, Pharmaceutical Institute, University of Bonn, An der Immenburg 4, 53121 Bonn, Germany.

[2]Department of Chemistry and Pharmacy, Medicinal Chemistry, Friedrich-Alexander-University Erlangen-Nürnberg, Nikolaus-Fiebiger-Straße 10, 91058 Erlangen, Germany.

[3]Molecular, Cellular and Pharmacobiology Section, Institute for Pharmaceutical Biology, University of Bonn, Nussallee 6, 53115 Bonn, Germany.

[4]Department of Pharmaceutical Chemistry, University of Marburg, Marbacher Weg 8, 35037 Marburg, Germany.

[5]Life and Medical Sciences (LIMES) Institute, Immune and Tumor Biology, University of Bonn 53115 Bonn, Germany.

[6]Medical Clinic III for Oncology, Hematology, Immuno-Oncology and Rheumatology, University Hospital Bonn, University of Bonn, Venusberg-Campus 1, 53127 Bonn, Germany.

[7]Institute of Medicinal and Pharmaceutical Chemistry, Technische Universität Braunschweig, Beethovenstraße 55, 38106 Braunschweig, Germany.

Table of Contents

SUPPLEMENTAL FIGURES, TABLES, AND SCHEME S4
NMR SPECTRA OF SYNTHESIZED COMPOUNDS S13
HPLC CHROMATOGRAMS OF TARGET COMPOUNDS S38
REFERENCES S48

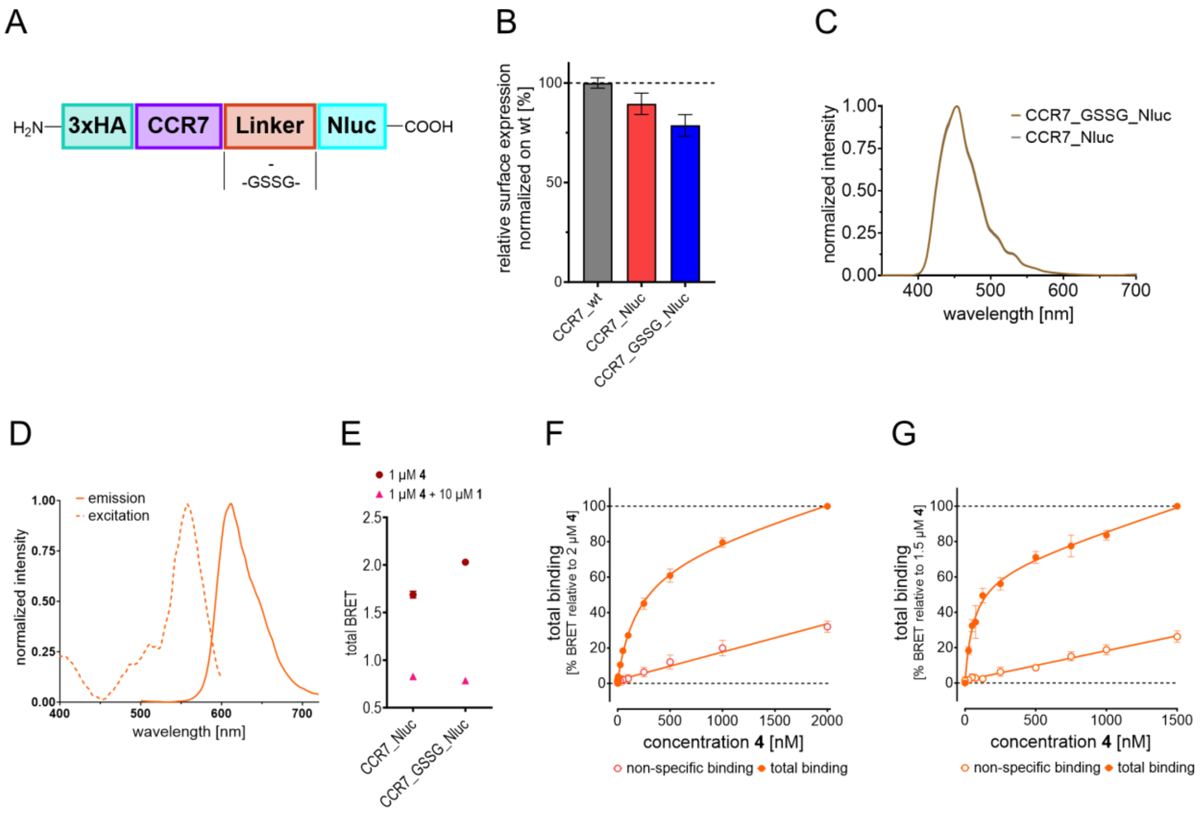

Figure S1. Development of a cell-free and cellular NanoBRET-based binding assay for the IABS of CCR7. A) Schematic representation of the genetic C-terminally Nluc-labeled constructs of CCR7 that were investigated in the course of assay development. B) Expression level of different 3xHA-CCR7-Nluc constructs detected via ELISA and normalized to the expression of wild-type 3xHA-CCR7 (CCR7_wt). Bar diagram represents the mean values ± SEM ($n \geq 3$) with each test performed in quadruplicate. The experiment indicates that all CCR7-Nluc constructs are well-expressed in HEK293T cells. C) Emission spectra of membrane preparations from HEK293T cells transiently overexpressing the CCR7_Nluc and CCR7_GSSG_Nluc construct, respectively. D) Spectral properties of fluorescent ligand Mz437 (**4**). Absorption and fluorescence emission spectra were normalized to the respective maximum signal of each sample. E) Representative NanoBRET assay windows for Mz437 (**4**), generated with membrane preparations from HEK293T cells expressing different CCR7-Nluc constructs. Tests were performed in duplicate. The signals for non-specific binding (full displacement of **4**, red triangles) were detected in the presence of **4** (1 µM) and **1** (10 µM), whereas the signals obtained for the vehicle controls represent total binding of **4** at a concentration of 1 µM (blue circles). As the application of CCR7_GSSG_Nluc resulted in a slightly larger assay window, we used this construct for further investigations. F) Binding curves for total and non-specific binding (triplicate measurement, $n = 3$) of **4** to CCR7_GSSG_Nluc membranes. G) Binding curves for total and non-specific binding (quadruplicate measurement, $n = 4$) of **4** to live HEK293T cells transiently overexpressing CCR7_GSSG_Nluc.

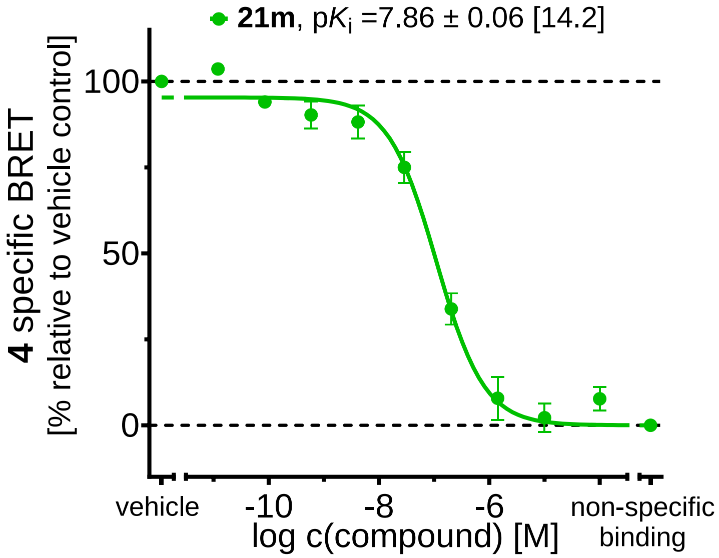

Figure S2. Cellular competition binding curve and pK_i value (mean ± SEM, quadruplicate measurement, n = 3) for **21m** obtained with **4** (500 nM) and live HEK293T cells transiently expressing CCR7_GSSG_Nluc. K_i value [nM] is given in square brackets.

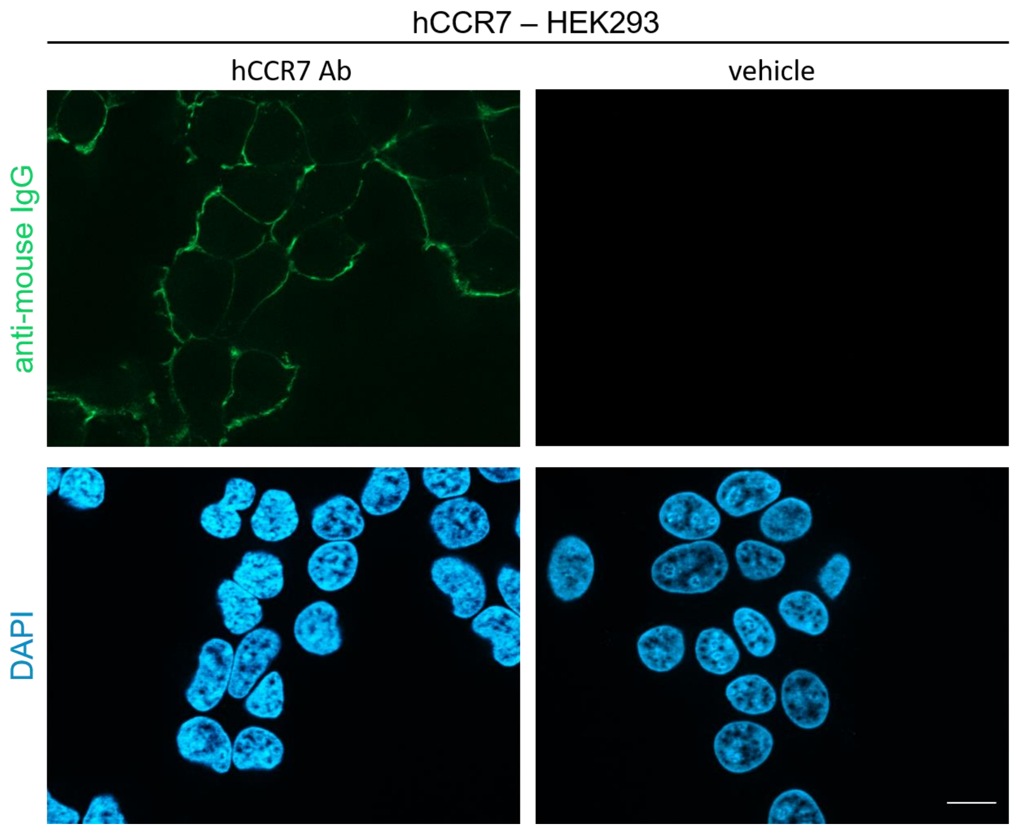

Figure S3. CCR7 immunostaining in the presence and absence of the primary hCCR7 antibody. Stable HEK293-CCR7 cells were fixed and immunostained with an anti-CCR7 antibody followed by addition of goat anti-mouse IgG conjugated to FITC. In the experiment designated vehicle control, the primary antibody was omitted in the staining protocol. Scale bar is 10 µm.

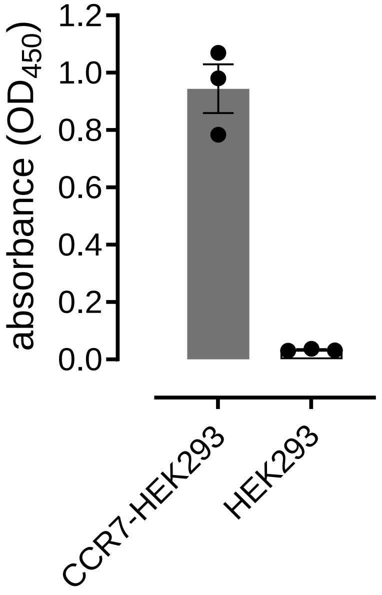

Figure S4. Cell surface expression of CCR7 in HEK293 cells. Plasma-membrane-resident CCR7 was assayed by ELISA in naïve and a stably transfected isogenic CCR7-HEK293 cell clone using an anti-CCR7 antibody (R&D Systems, MAB197, diluted 1:300). Absorbance values obtained for naïve HEK293 cells reflect nonspecific labeling of the cells. Data are mean values ± SEM from three independent experiments, each performed in technical triplicate.

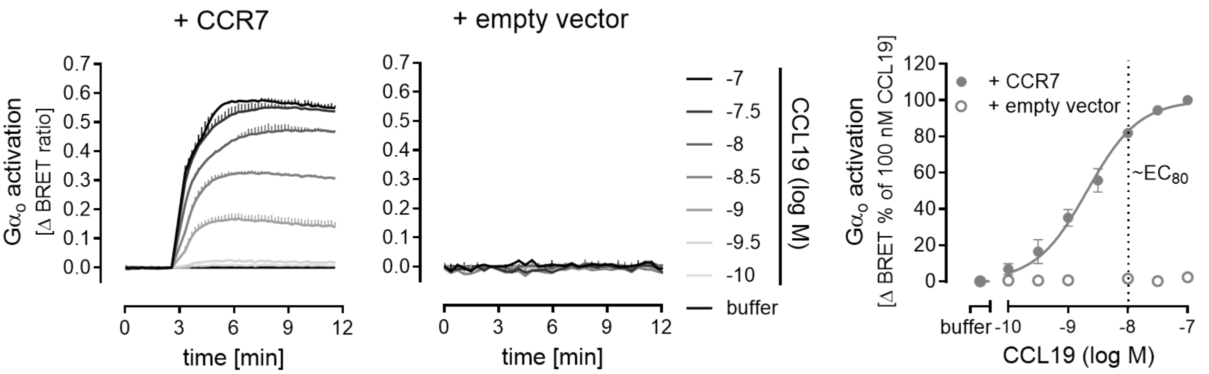

Figure S5. Concentration-dependent Go activation of CCR7 by CCL19. Representative kinetic recordings of BRET changes between masGRK3ct-Nluc and $G\alpha_o$-derived Gβγ (Venus-labeled) in HEK293T cells transiently transfected with hCCR7 or empty pcDNA3.1 control vector after treatment with increasing concentrations of CCL19. The summarized concentration-effect curve is shown as means ± SEM of n= 6 independent experiments for hCCR7 and as means ± SEM of one biological replicate performed in technical triplicate for vector transfected control cells. CCR7 binders were tested against 10 nM CCL19 (~EC_{80} as indicated in the curve).

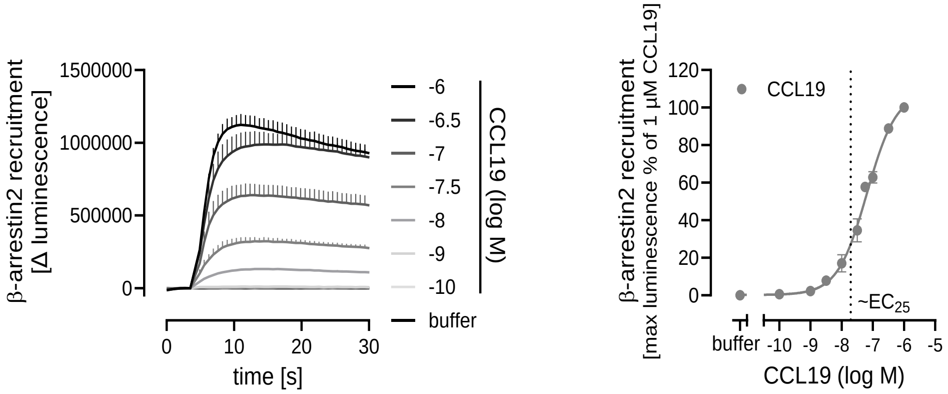

Figure S6. CCL19-induced β-arrestin2 recruitment to CCR7. Representative traces of NanoBiT complementation between CCR7-SmBiT and LgBiT-β-arrestin2 mutant (R393E, R395E) in transiently transfected HEK293T cells after stimulation with CCL19. Averaged concentration-effect curve derived from the traces shows means ± SEM of n= 2-5 independent experiments. CCR7 binders were tested against 18 nM CCL19 (~EC_{25} as indicated in the curve).

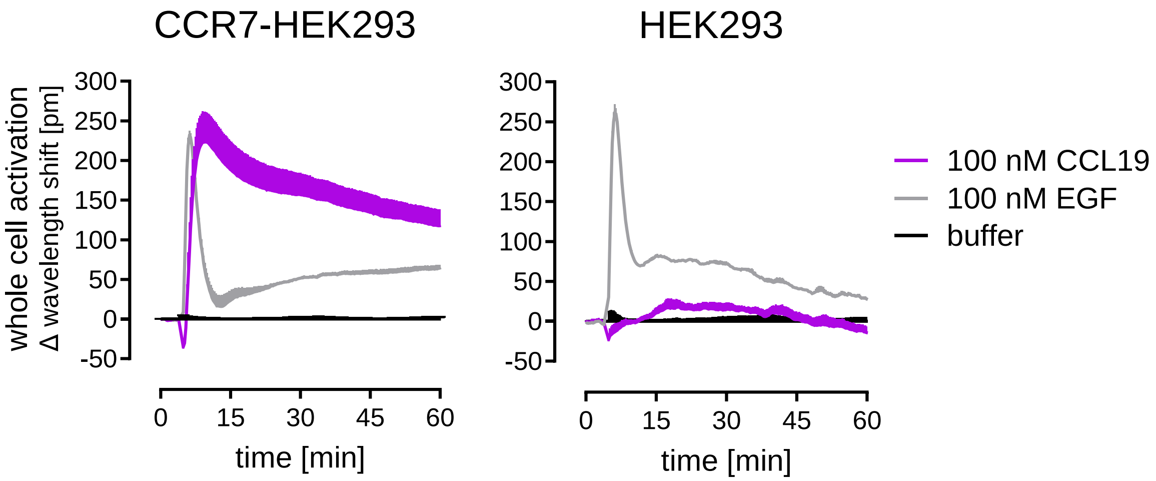

Figure S7. Label-free whole cell activation of HEK293 cell lines by CCL19. Representative real-time whole cell activation profiles of CCL19 evoked in a stable isogenic hCCR7-HEK293 cell line and in naïve HEK293 cells. Epidermal growth factor (EGF) is used as viability control. Data are means + SEM and are representative of three independent experiments, each performed in technical triplicates.

A

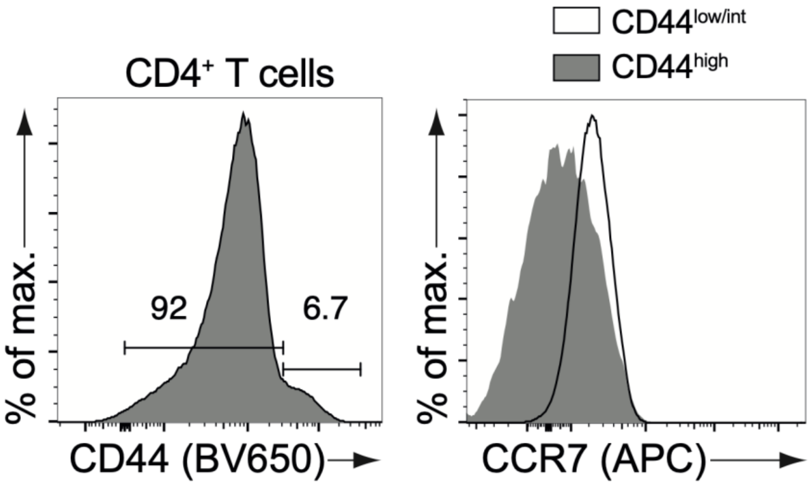

B

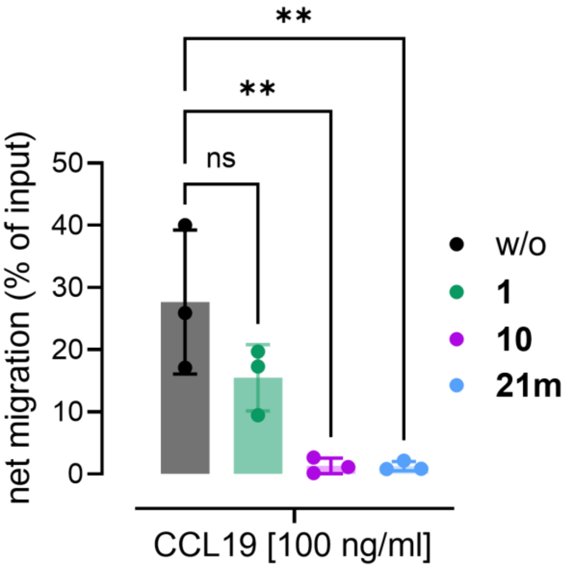

Figure S8. CCR7 expression and effect of intracellular CCR7 binders on CCL19-mediated migration of murine CD4^{+} T cells. A) CD4^{+} T cells are CCR7-high. CCR7 expression in naive (CD44$^{low/int}$) and activated (CD44^{high}) CD4^{+} T cells from skin-draining lymph nodes of wild-type C57BL/6 mice. B) Impact of **1**, **10**, and **21m** on CCR7-mediated transmigration of murine CD4^{+} T cells towards a 100 ng/ml CCL19 gradient. at a final concentration of 10 µM. Statistical significance was calculated with a one-way ANOVA with Dunnett`s post-hoc analysis; **, $P < 0.01$; ns, not significant.

Table S1. Kinetic parameters detected for the interaction between Mz437 (**4**) and CCR7_GSSG_Nluc using our membrane-based NanoBRET assay and by applying the indicated conditions. A) Dissociation at room temperature. B) Association at room temperature. For the calculation of k_{on}, a pre-determined k_{off} of 0.2234 min^{-1} was used.

A

Dissociation at room temperature (membranes)

	k_{off} [min^{-1}]	t_R [min]
1000 nM	0.2414	4.14
500 nM	0.2036	4.91
250 nM	0.2253	4.44
mean	**0.2234**	**4.50**
SEM	0.0110	0.22

B

Association at room temperature (membranes)

	k_{on} [M^{-1} min^{-1}]
1000 nM	5003923
	4576441
500 nM	3961621
	5455546
	3868786
250 nM	3210495
	3018297
100 nM	4501295
	4327296
	4852031
mean	**4277573**
SEM	244459

Table S2. Selectivity binding studies with **10** (SLW131) and **21m** (SLW132). CCR9 was selected because it is the most closely related chemokine receptor to CCR7.[1] CCR2 was selected as a second example of a chemokine receptor with a known intracellular allosteric binding pocket.[2] Cmp2105 (**1**) was used for comparison. NanoBRET-based binding studies were performed as previously described.[3,4] Tests were performed in triplicate (n = 3). K_i values are given in brackets.
[a]comp. = percentual competitive tracer displacement at given concentration

Compound	**CCR2**<br>**pK_i** ± SEM (K_i [nM]) or comp.	**CCR9**<br>**pK_i** ± SEM (K_i [nM]) or comp.[a]
**10**	13% comp. @ 20 µM	**5.83** ± 0.05 (1479)
**21m**	30% comp. @ 20 µM	**5.82** ± 0.02 (1514)
**Cmp2105 (1)**	3% comp. @ 10 µM	**6.28** ± 0.06 (530)

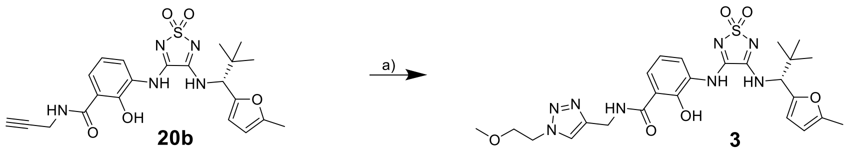

Scheme S1. Synthesis of the triazole-based CCR7 ligand-linker conjugate **3**. *Reagents and conditions*: a) 1-azido-2-methoxyethane, $CuSO_4 \cdot 5\,H_2O$, sodium ascorbate, TBTA, water/*tert*-BuOH/DMF mixture (1:1:1 (*v/v/v*)), rt, 2 days, 36%.

NMR SPECTRA OF SYNTHESIZED COMPOUNDS

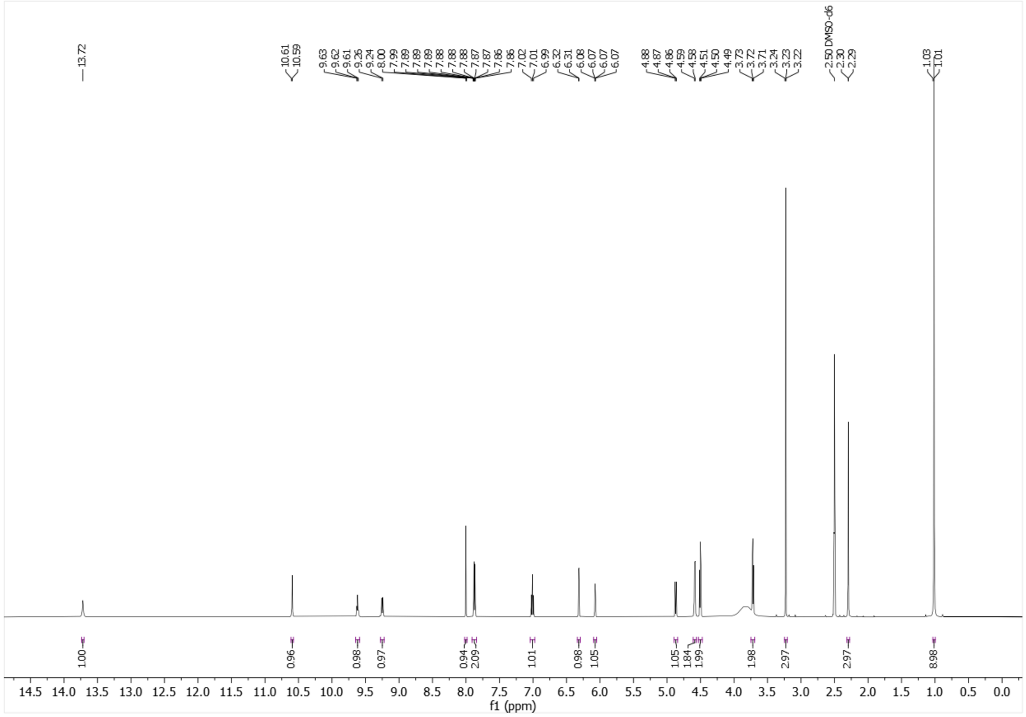

^{1}H-NMR (500 MHz, DMSO-d_6) of **3**.

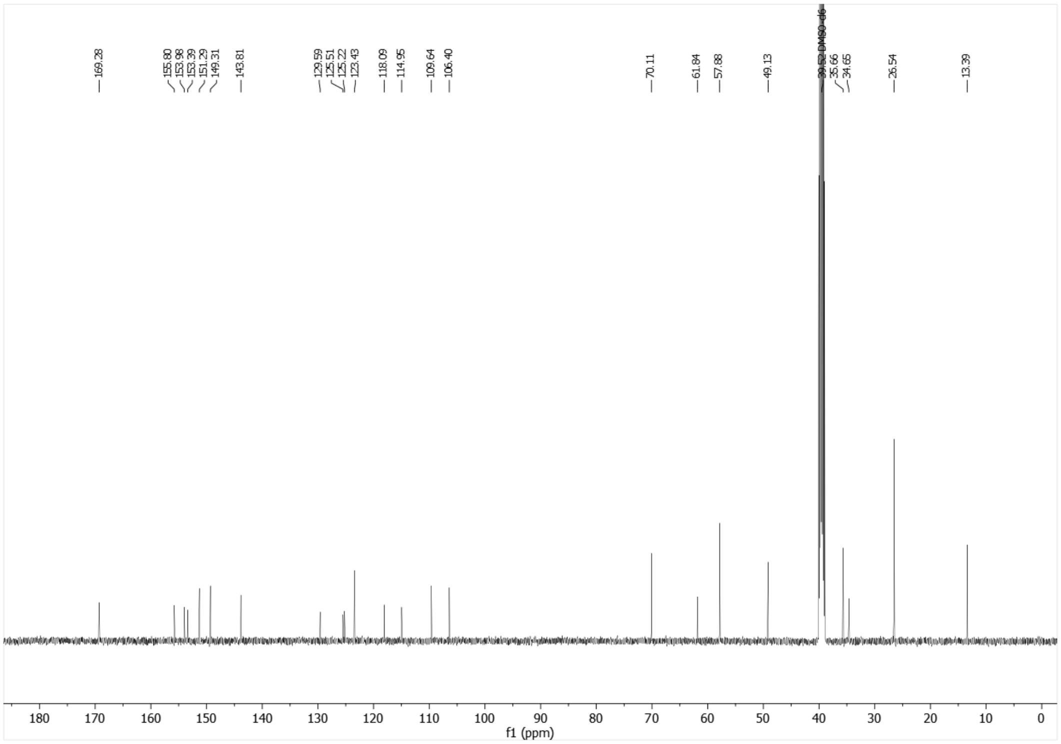

^{13}C-NMR (126 MHz, DMSO-d_6) of **3**.

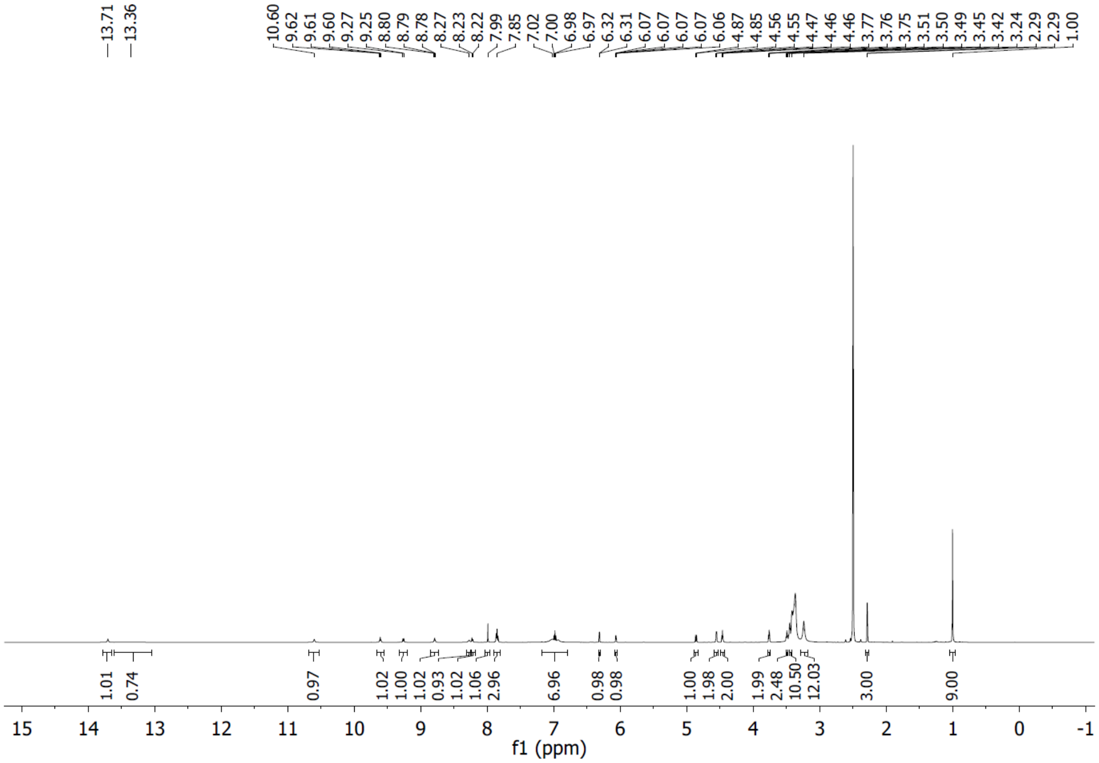

^{1}H-NMR (600 MHz, DMSO-d_6) of Mz437 (**4**).

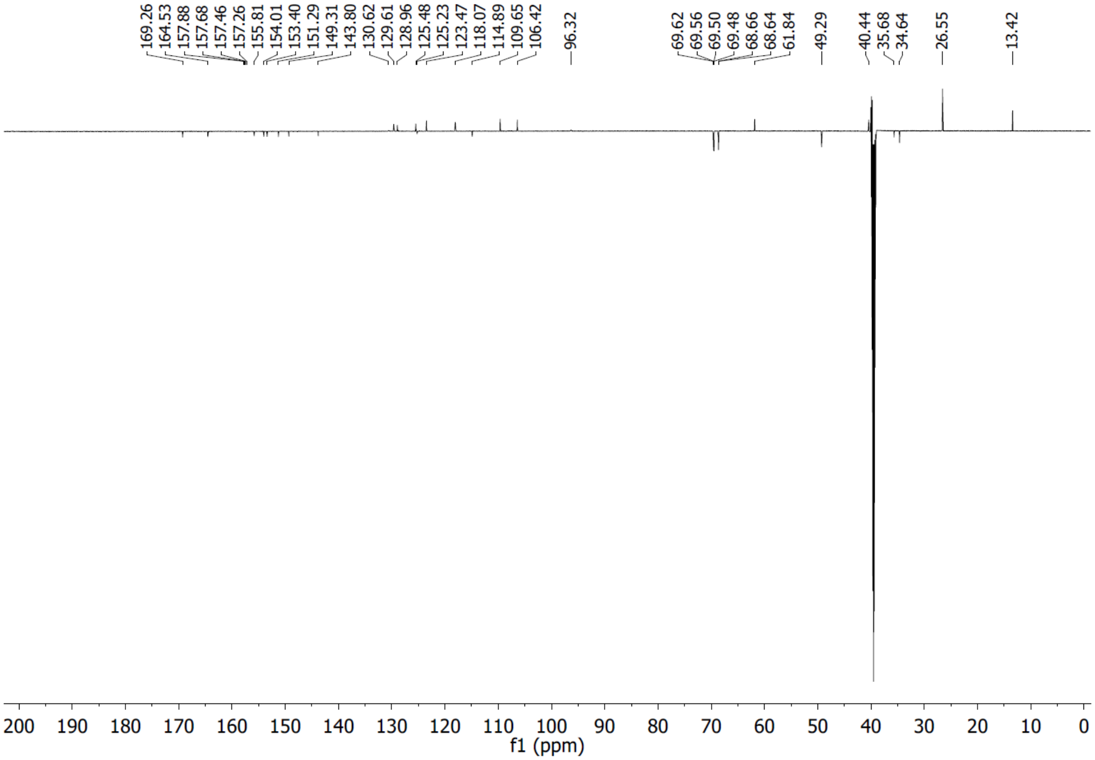

^{13}C-NMR (151 MHz, DMSO-d_6) of Mz437 (**4**).

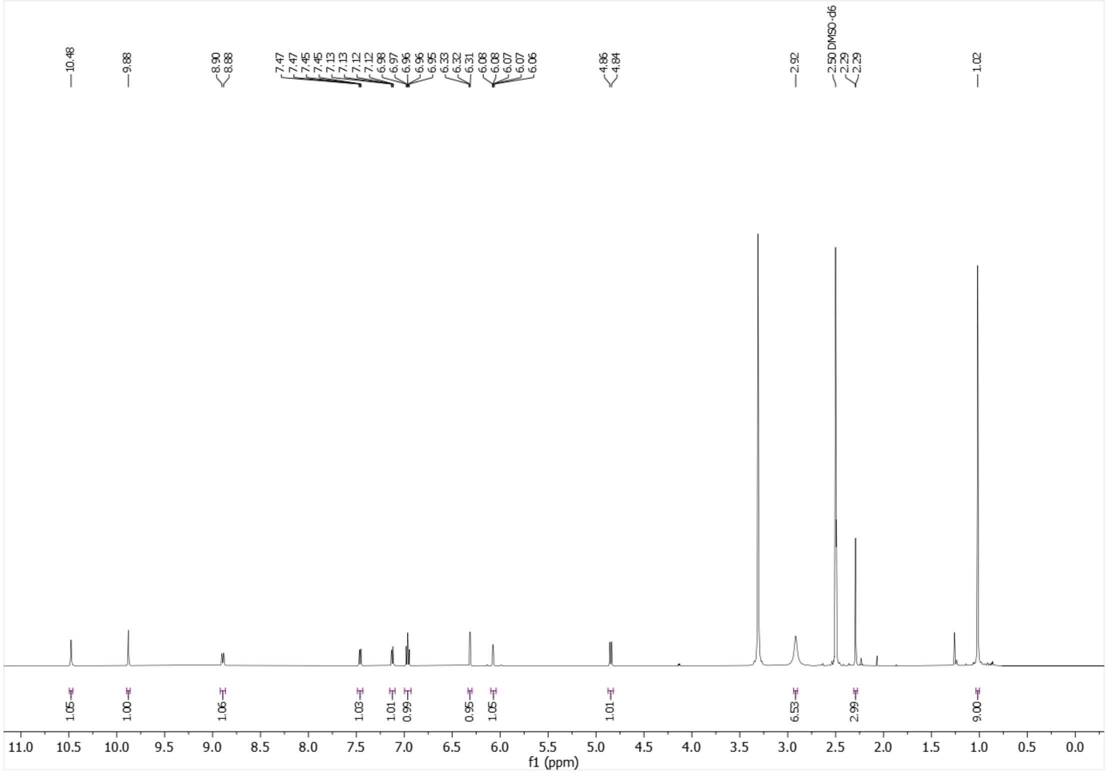

^{1}H-NMR (500 MHz, DMSO-d_6) of SLW131 (**10**).

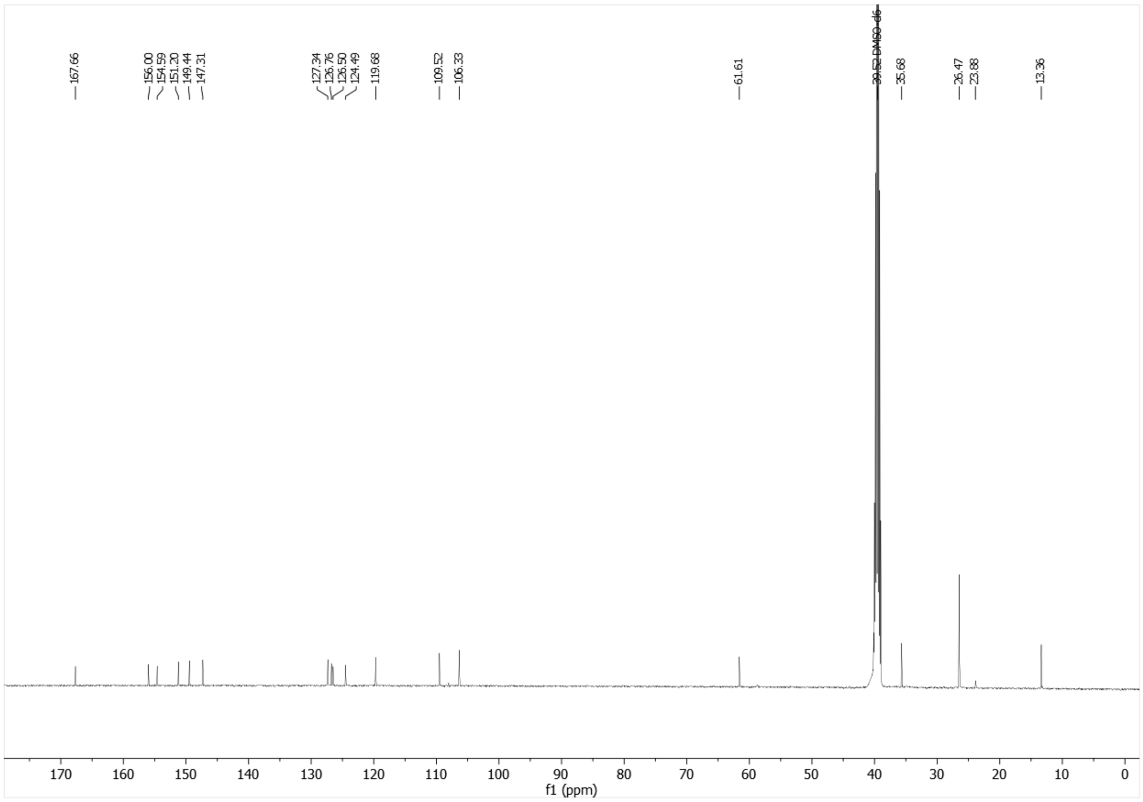

^{13}C-NMR (126 MHz, DMSO-d_6) of SLW 131 (**10**).

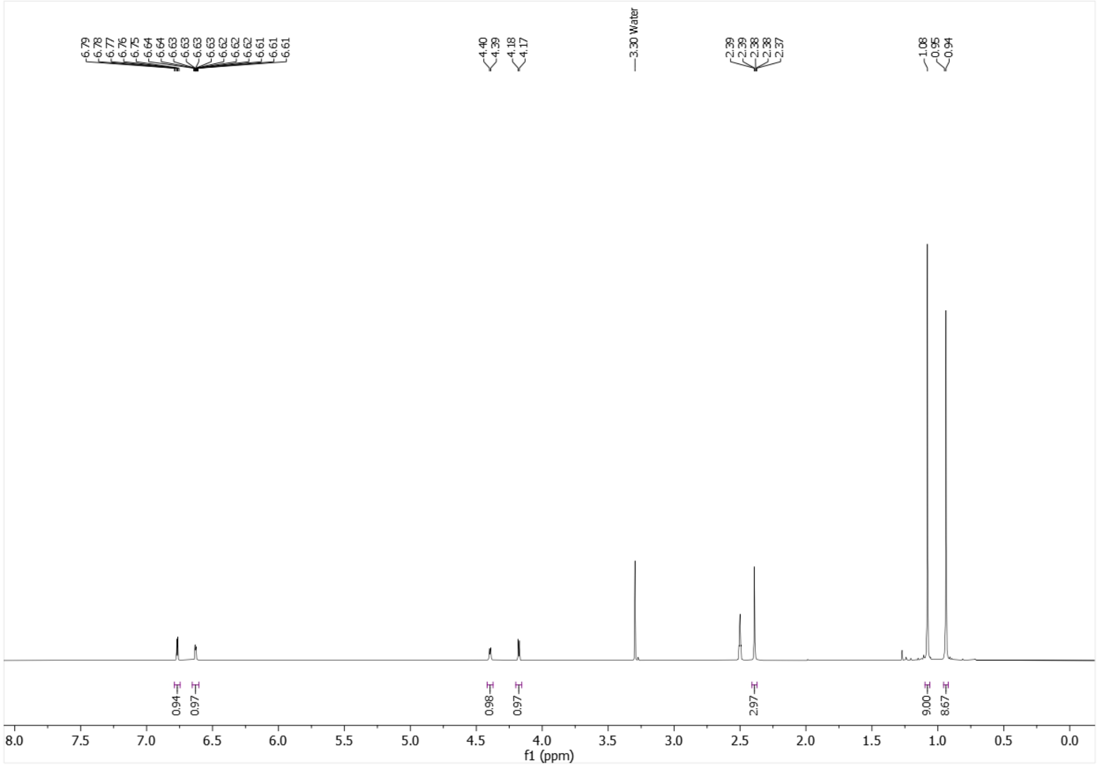

^{1}H-NMR (500 MHz, DMSO-d_6) of **14d**.

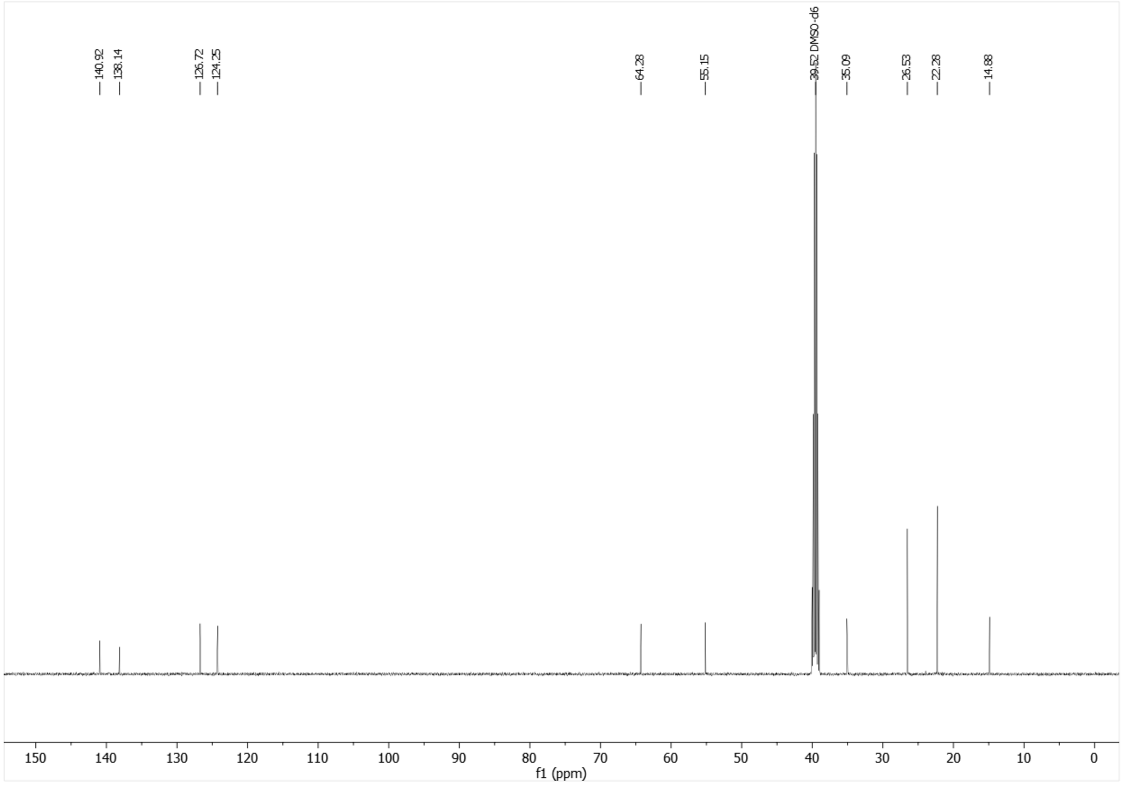

^{13}C-NMR (126 MHz, DMSO-d_6) of **14d**.

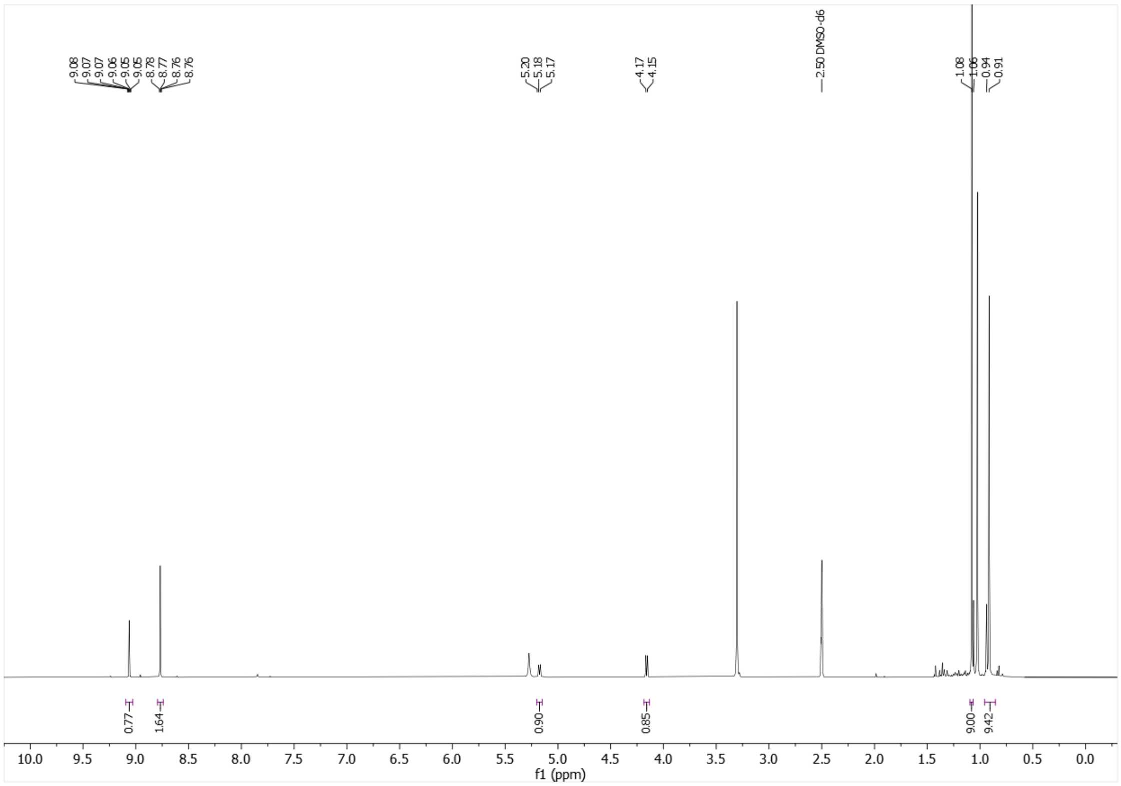

^{1}H-NMR (500 MHz, DMSO-d_6) of **14e**.

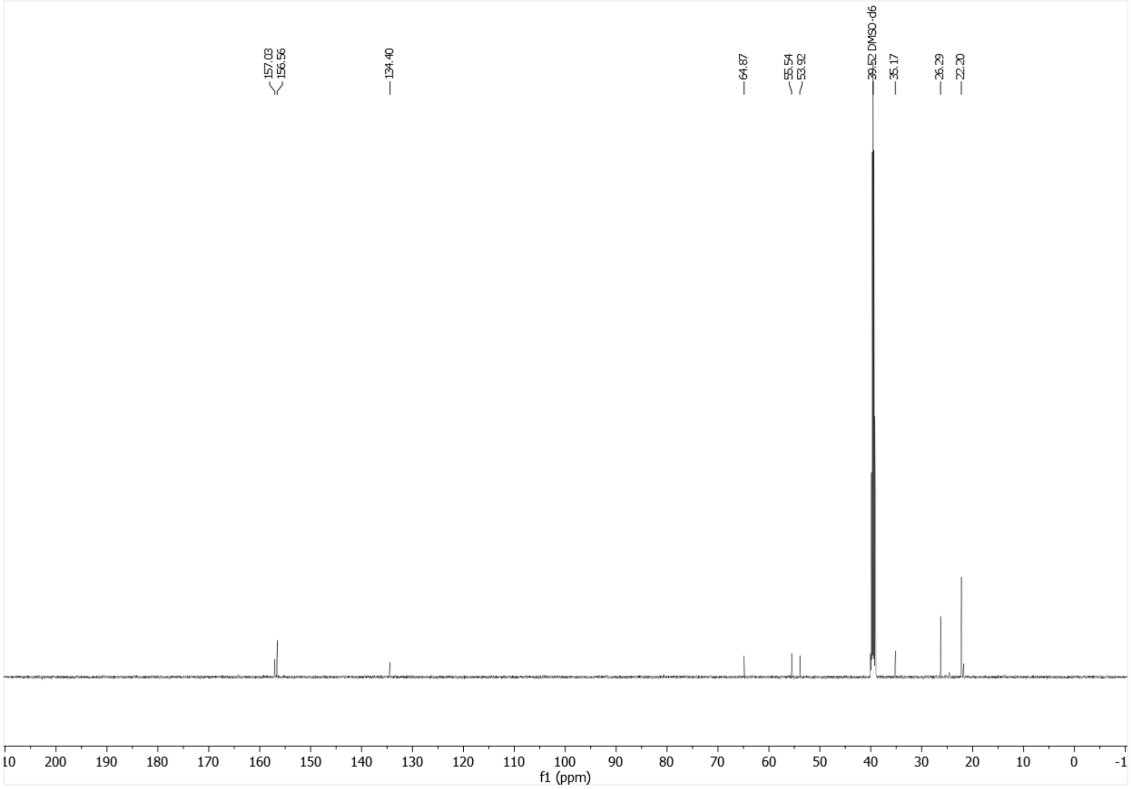

^{13}C-NMR (126 MHz, DMSO-d_6) of **14e**.

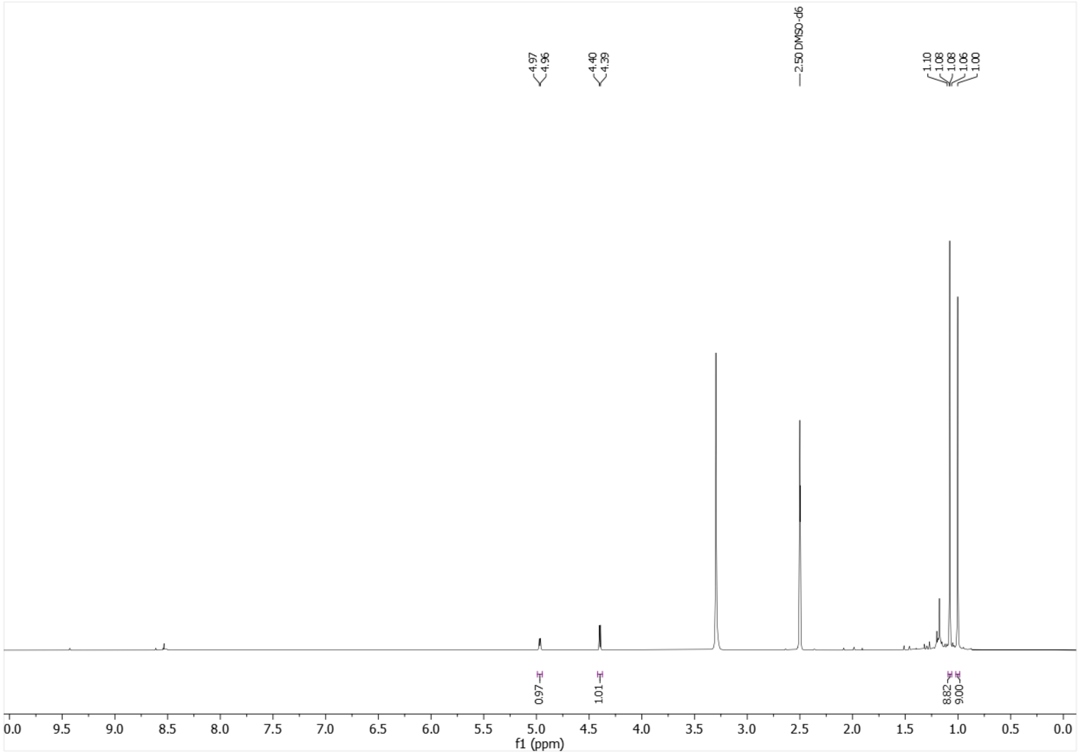

^{1}H-NMR (500 MHz, DMSO-d_6) of **14f**.

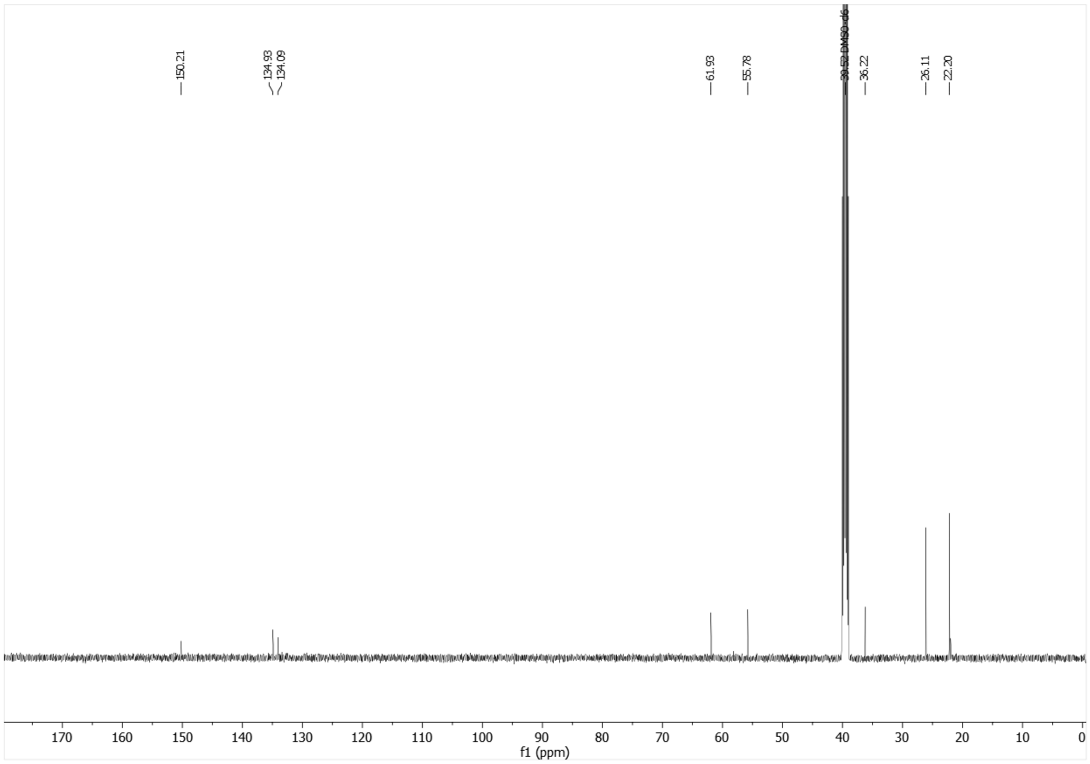

^{13}C-NMR (126 MHz, DMSO-d_6) of **14f**.

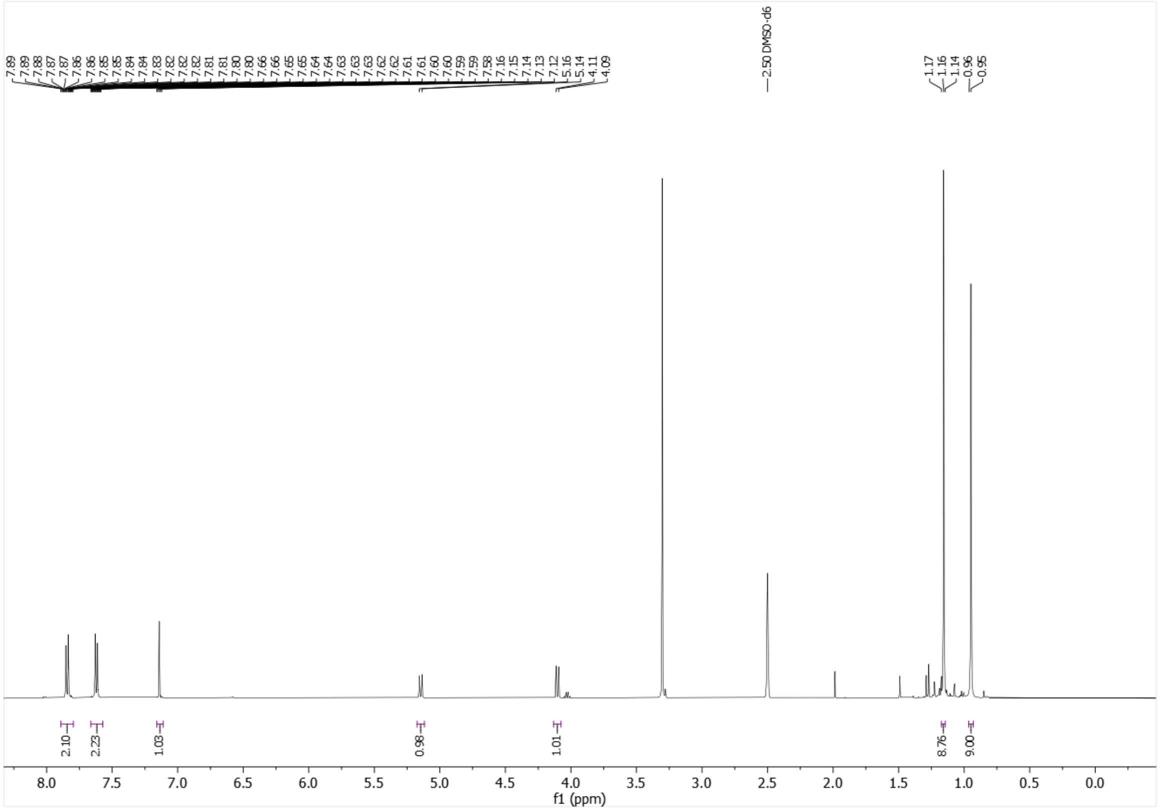

^{1}H-NMR (500 MHz, DMSO-d_6) of **14g**.

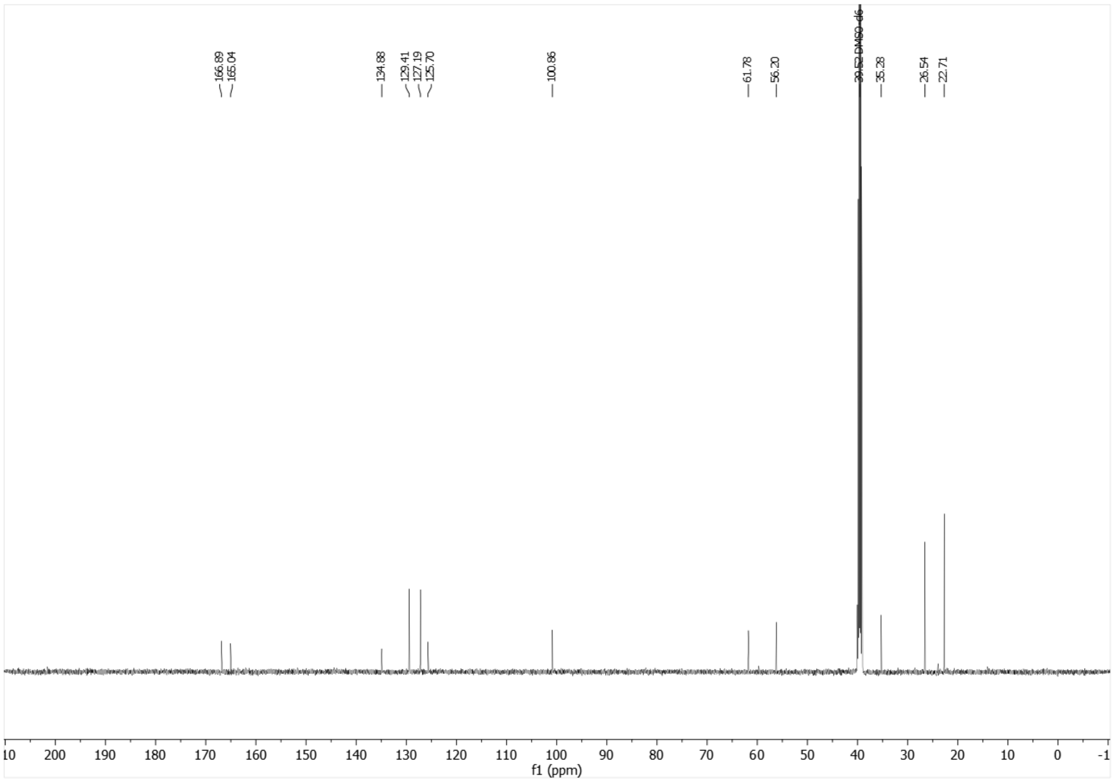

^{13}C-NMR (126 MHz, DMSO-d_6) of **14g**.

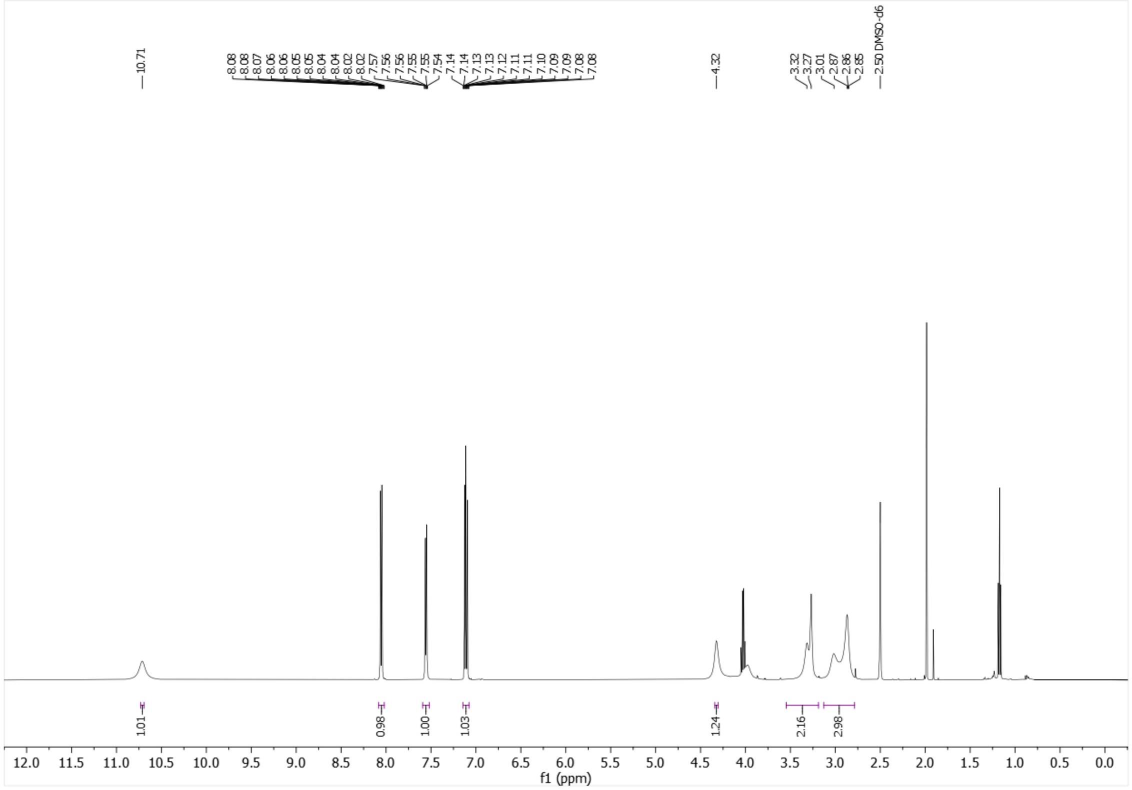

^{1}H-NMR (500 MHz, DMSO-d_6) of **16c**.

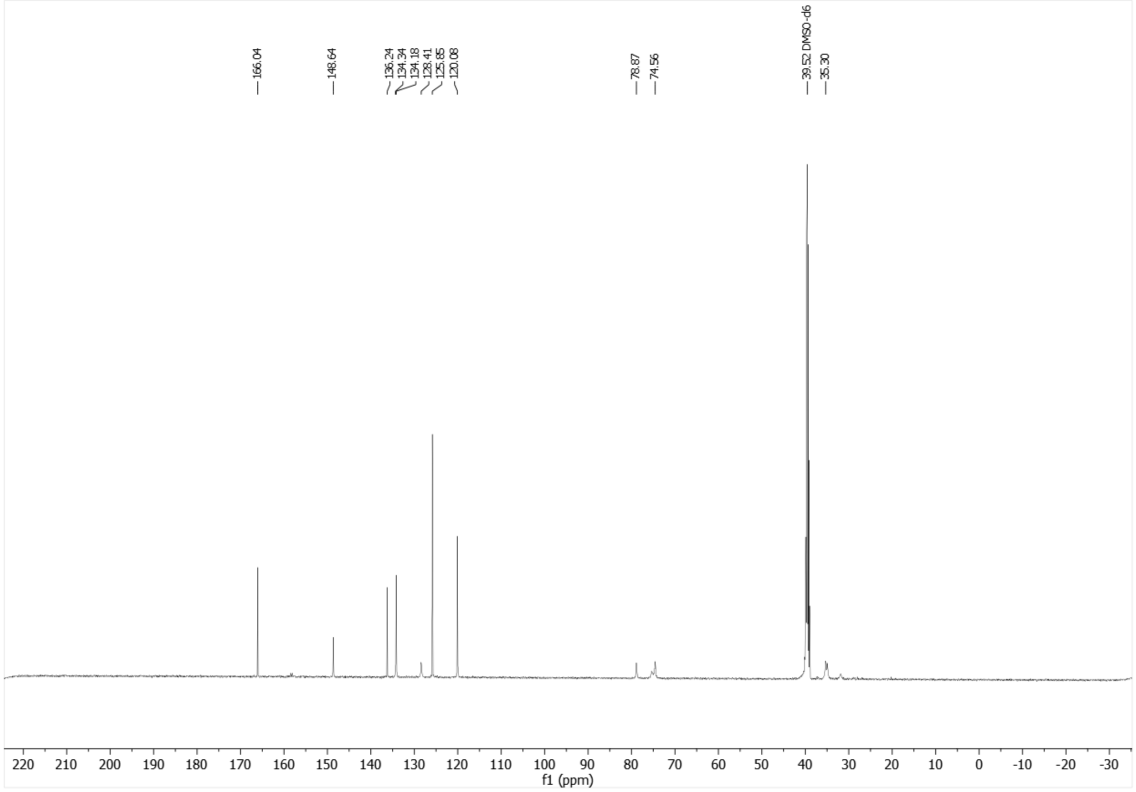

^{13}C-NMR (126 MHz, DMSO-d_6) of **16c**.

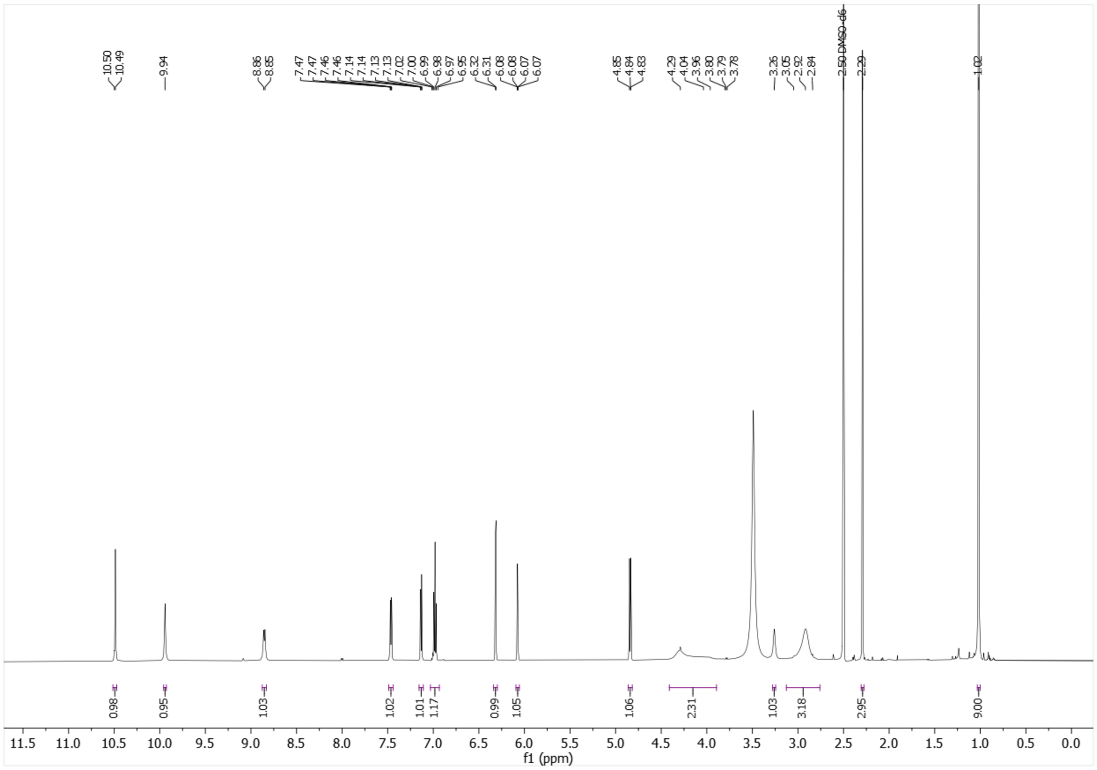

^{1}H-NMR (500 MHz, DMSO-d_6) of **20a**.

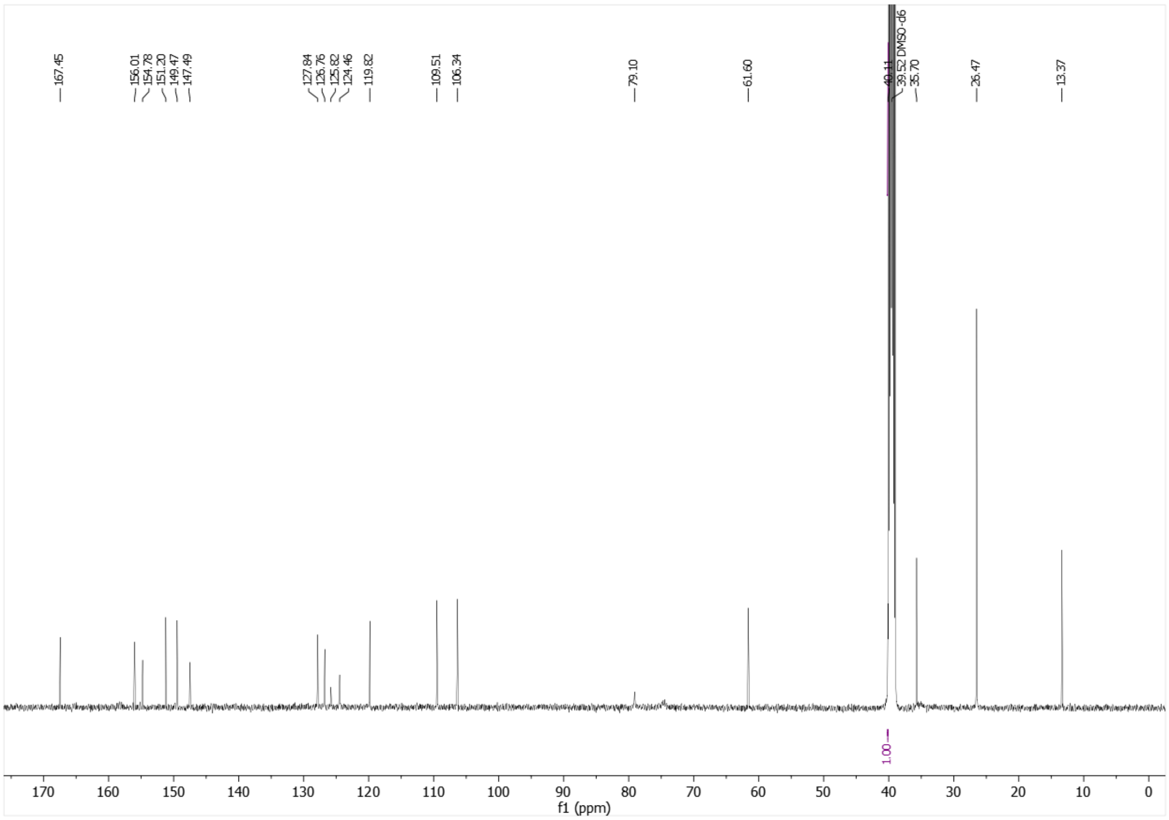

^{13}C-NMR (126 MHz, DMSO-d_6) of **20a**.

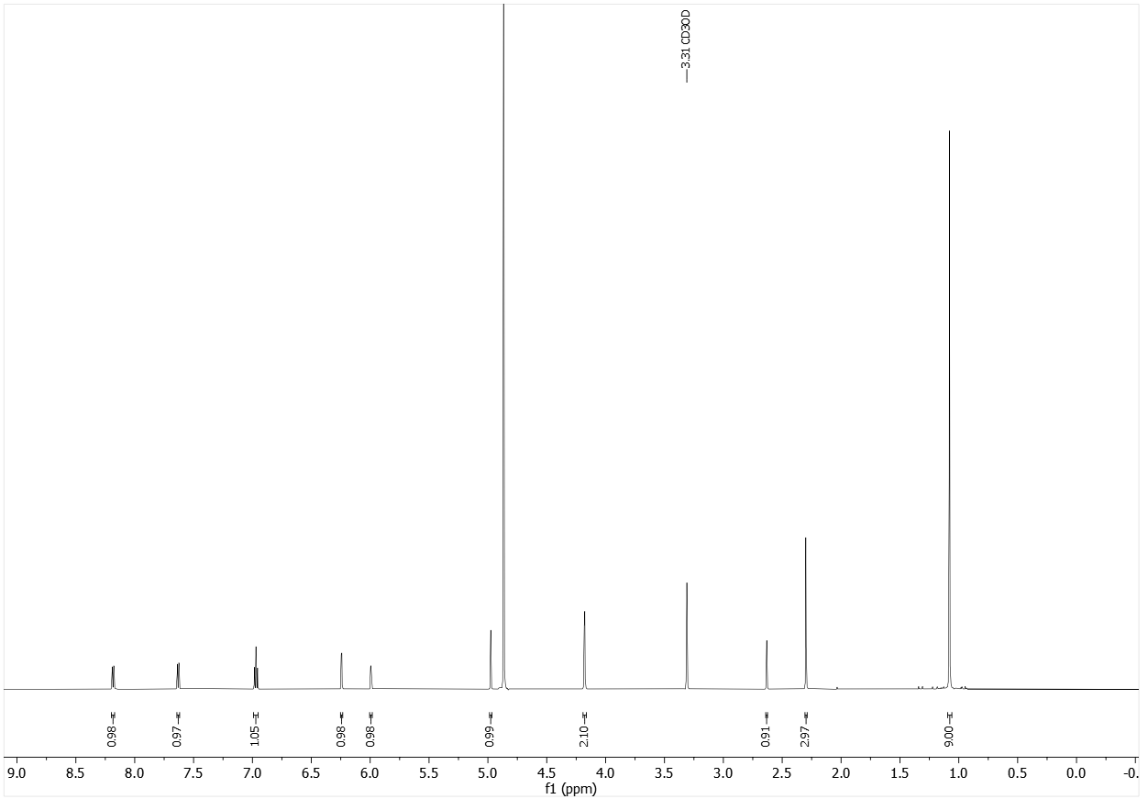

^{1}H-NMR (600 MHz, MeOD-d_4) of **20b**.

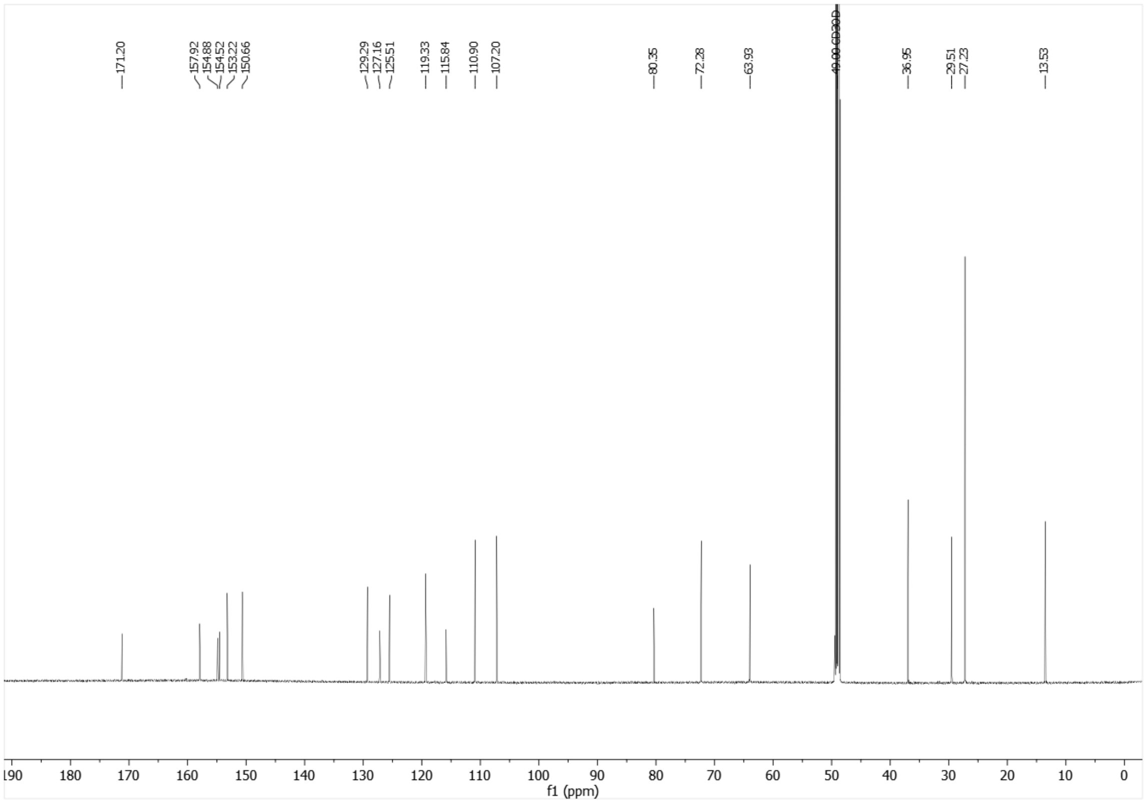

^{13}C-NMR (151 MHz, MeOD-d) of **20b**.

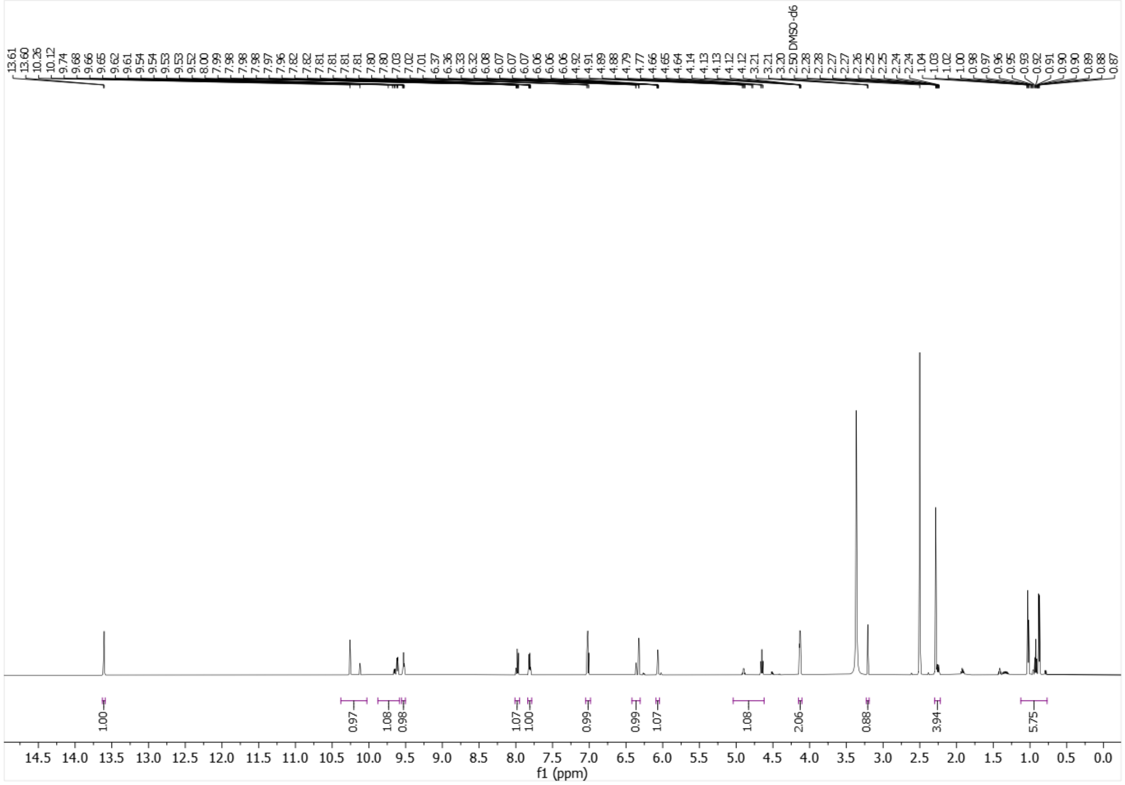

^{1}H-NMR (600 MHz, DMSO-d_6) of **20c**.

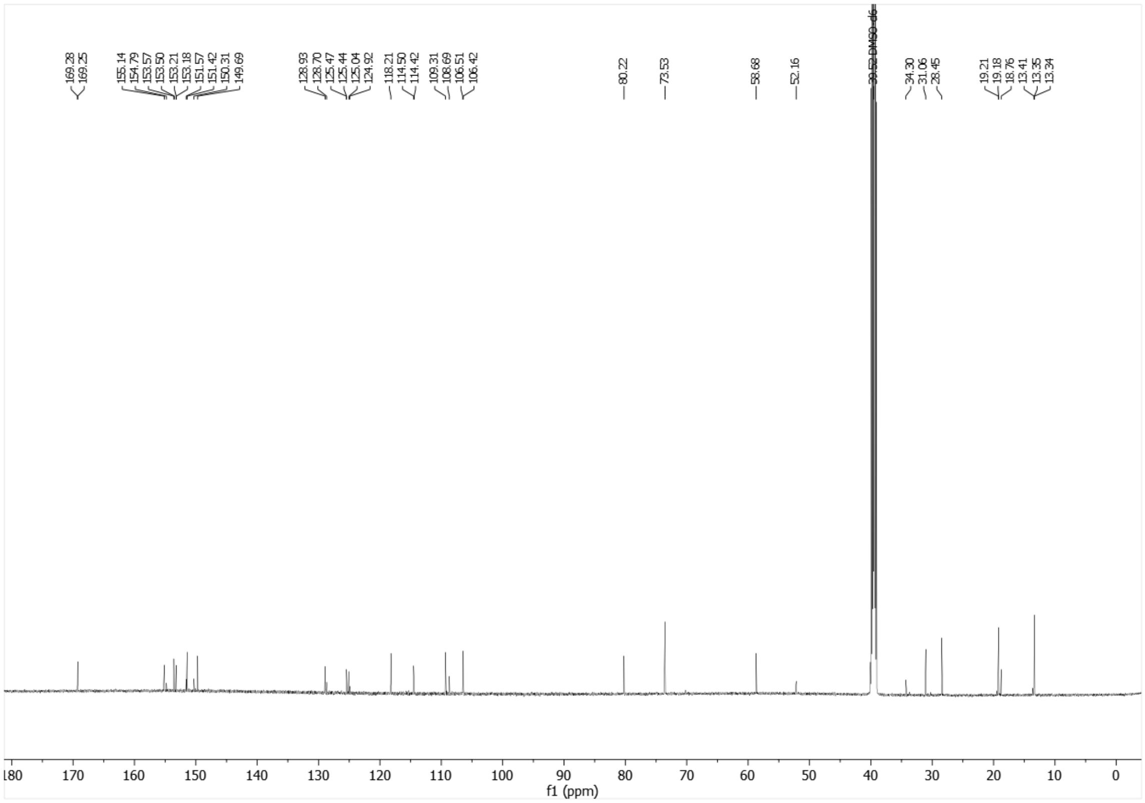

^{13}C-NMR (151 MHz, DMSO-d_6) of **20c**.

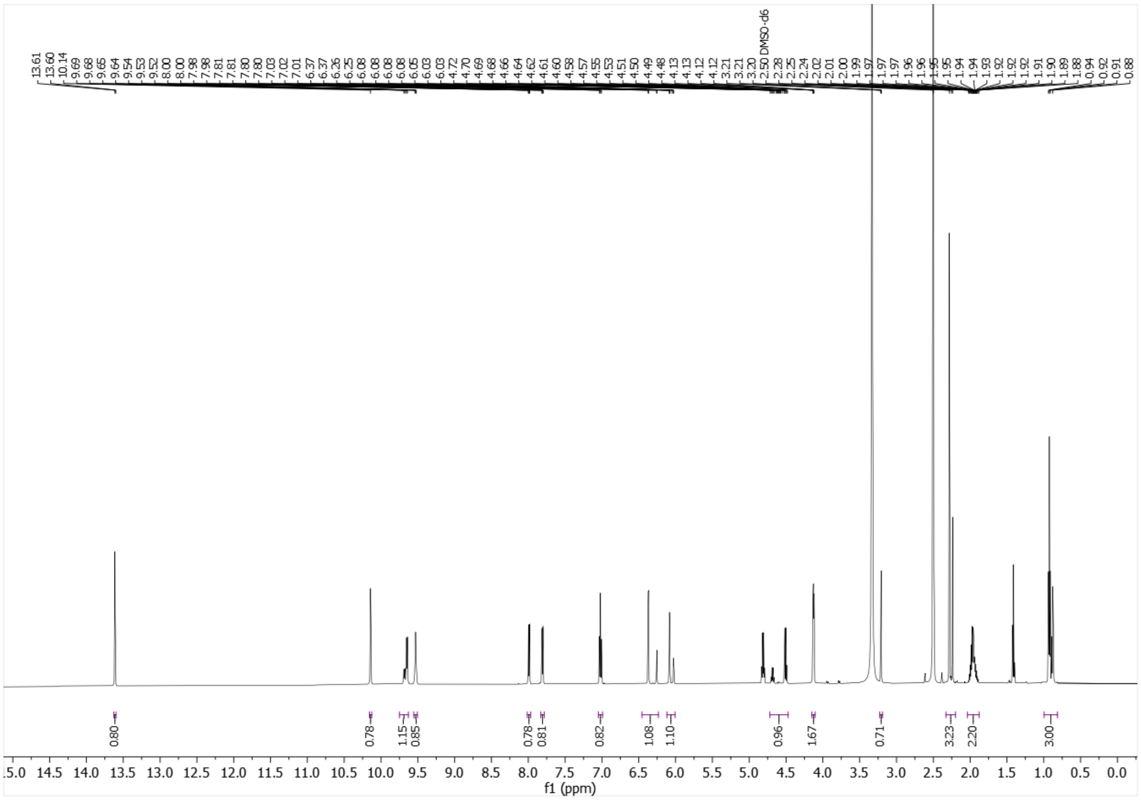

^{1}H-NMR (600 MHz, DMSO-d_6) of **20d**.

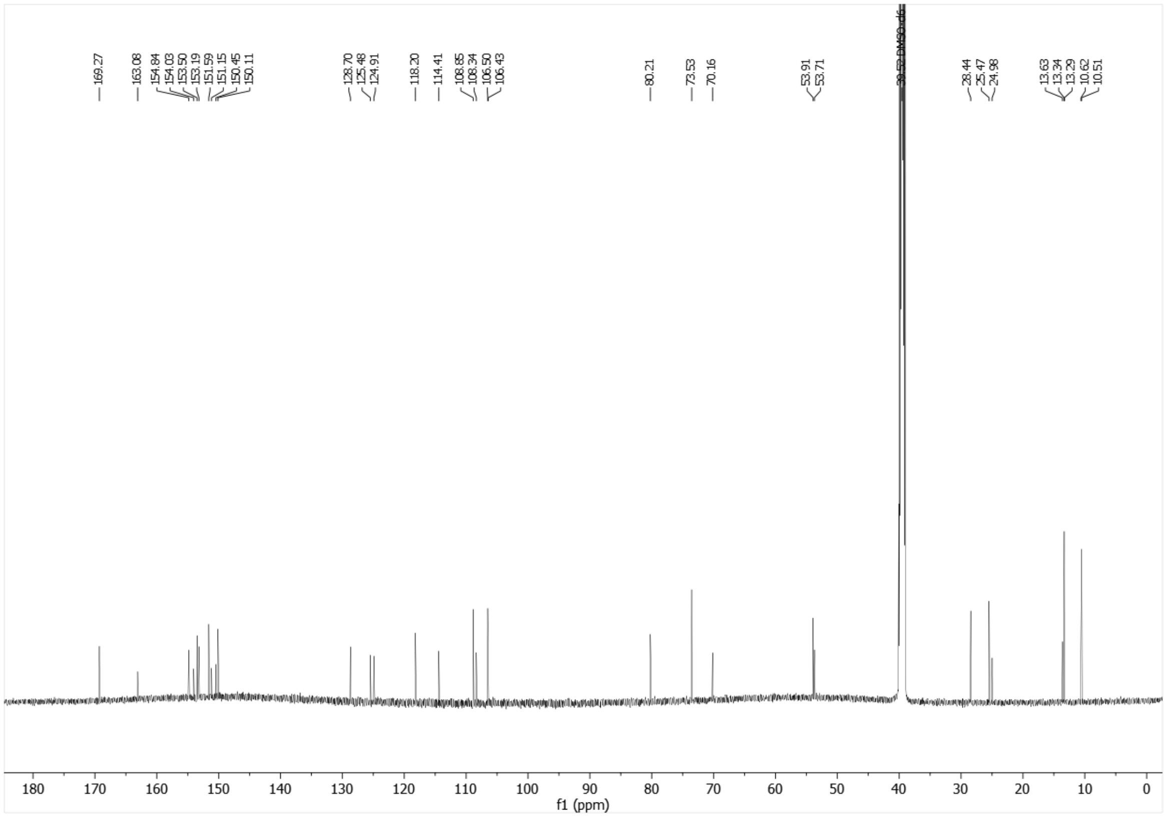

^{13}C-NMR (151 MHz, DMSO-d_6) of **20d**.

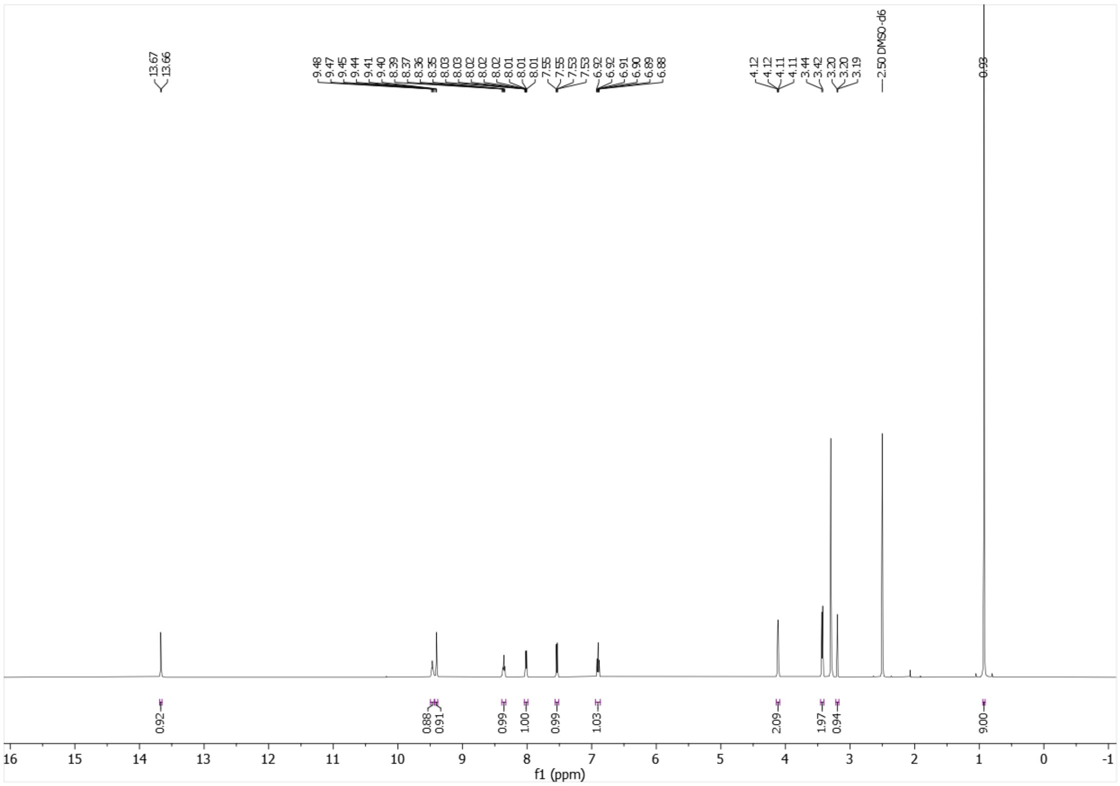

^{1}H-NMR (500 MHz, DMSO-d_6) of **21a**.

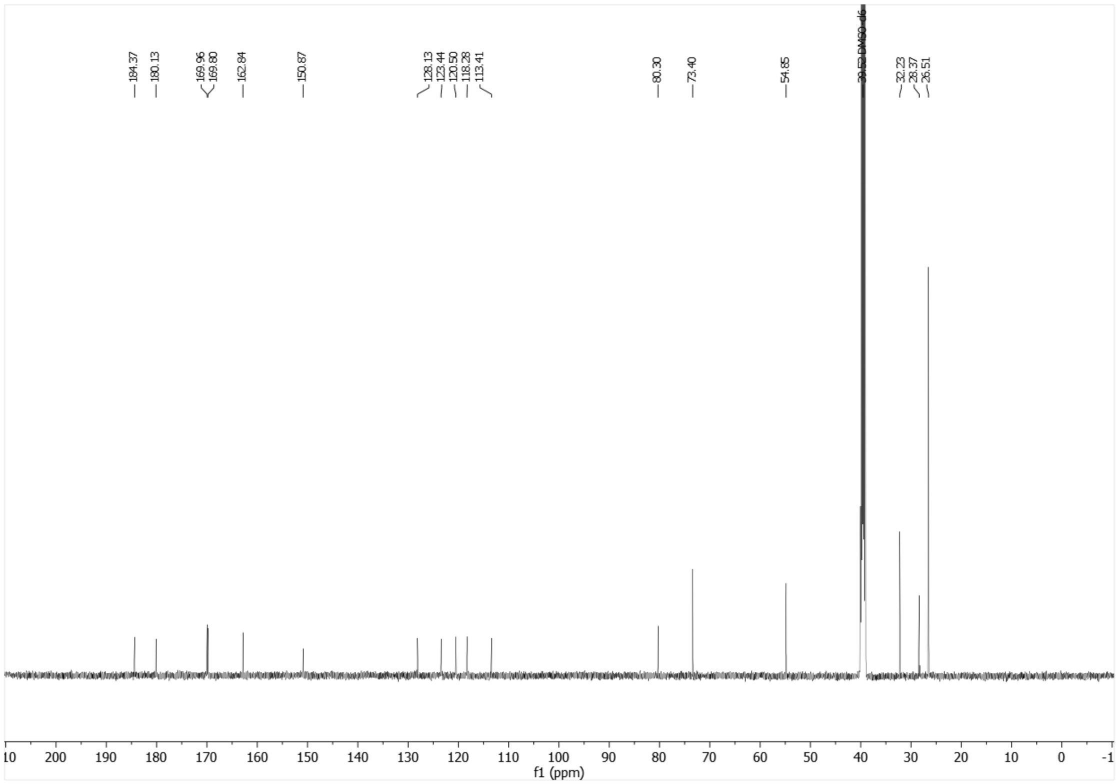

^{13}C-NMR (126 MHz, DMSO-d_6) of **21a**.

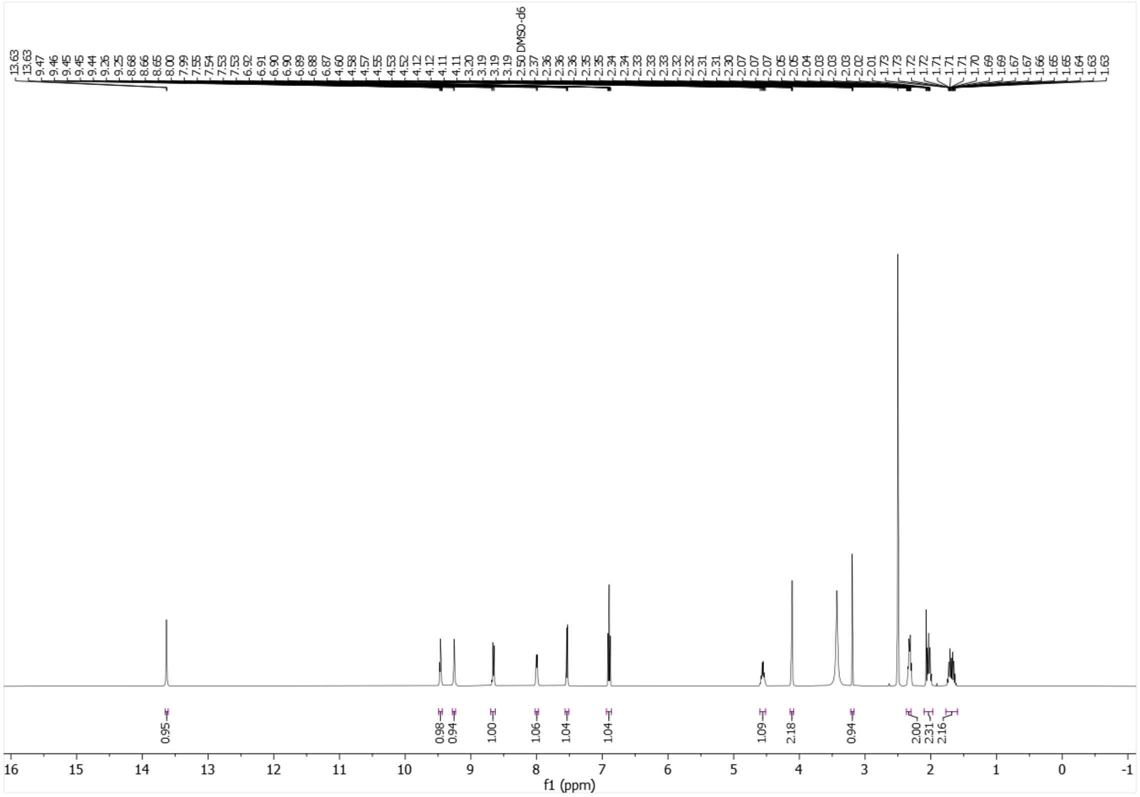

^{1}H-NMR (500 MHz, DMSO-d_6) of **21b**.

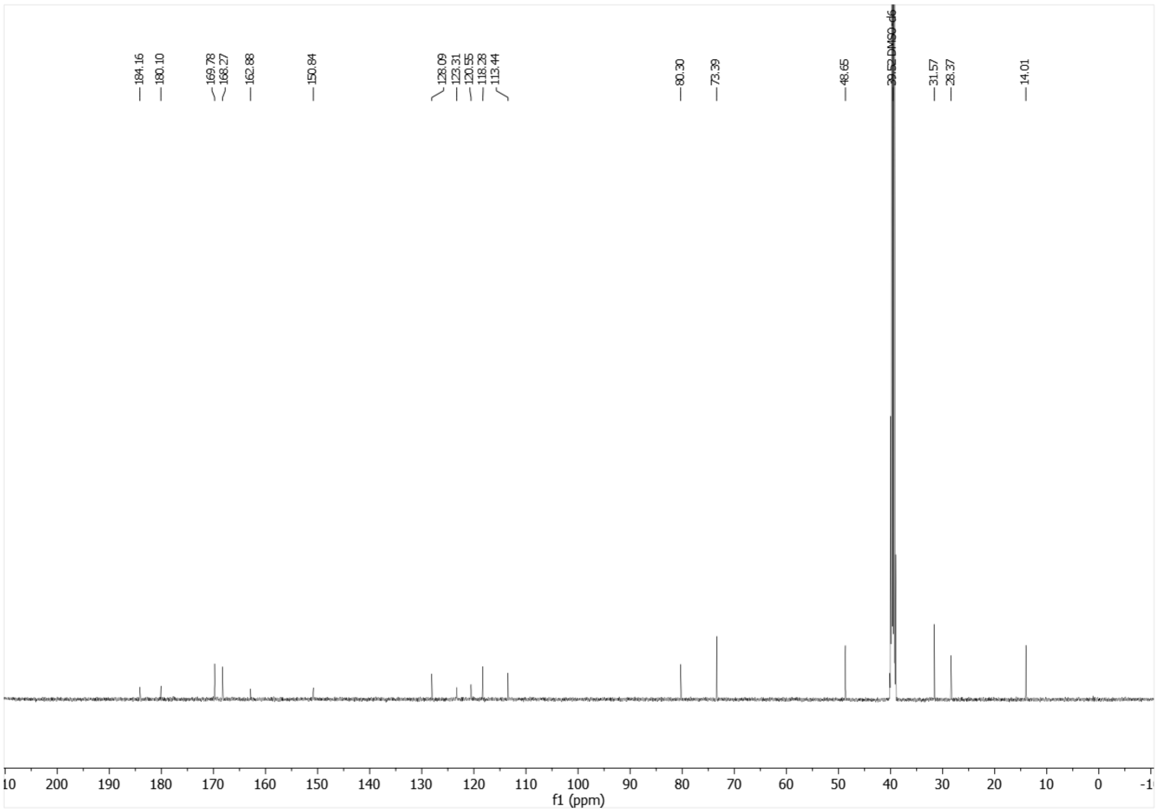

^{13}C-NMR (126 MHz, DMSO-d_6) of **21b**.

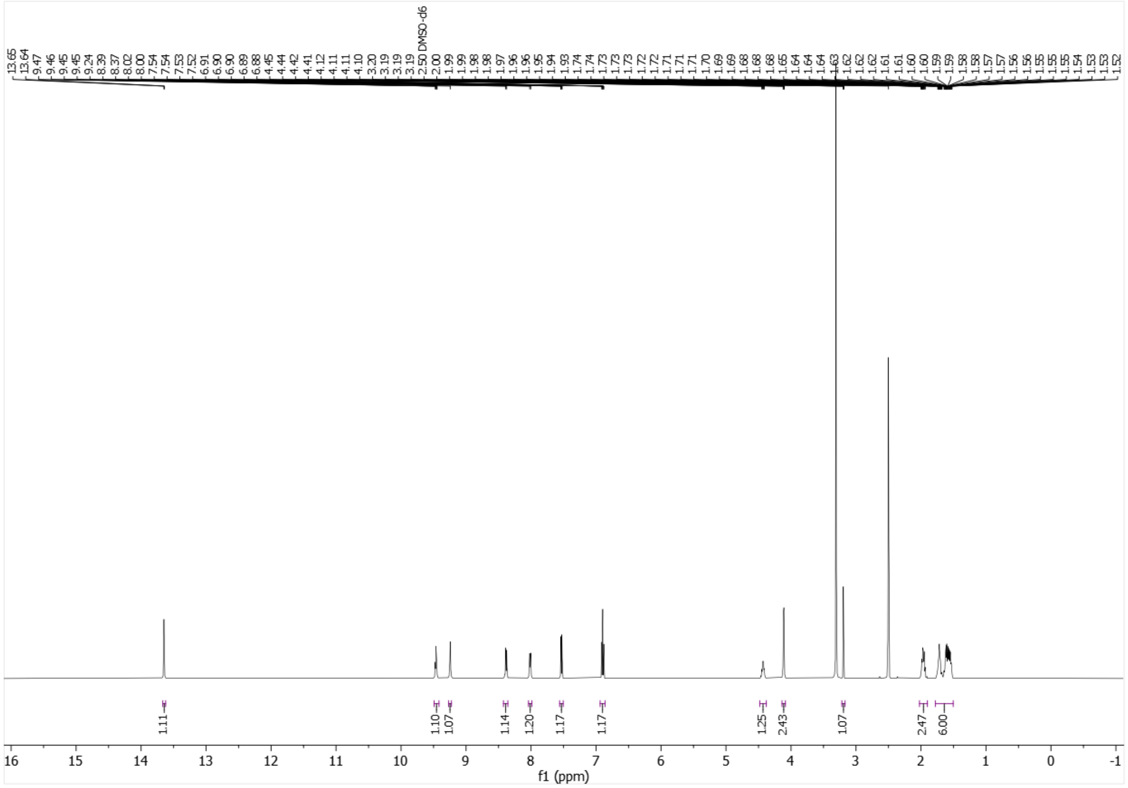

^{1}H-NMR (500 MHz, DMSO-d_6) of **21c**.

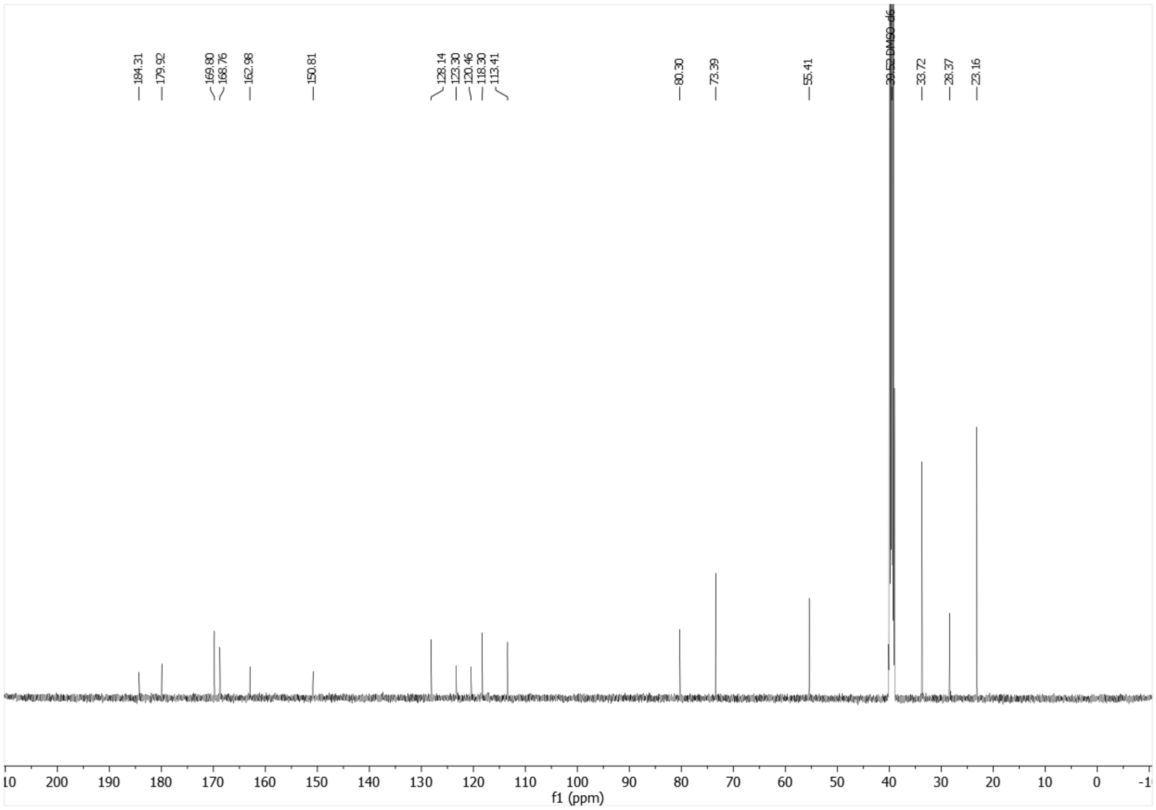

^{13}C-NMR (126 MHz, DMSO-d_6) of **21c**.

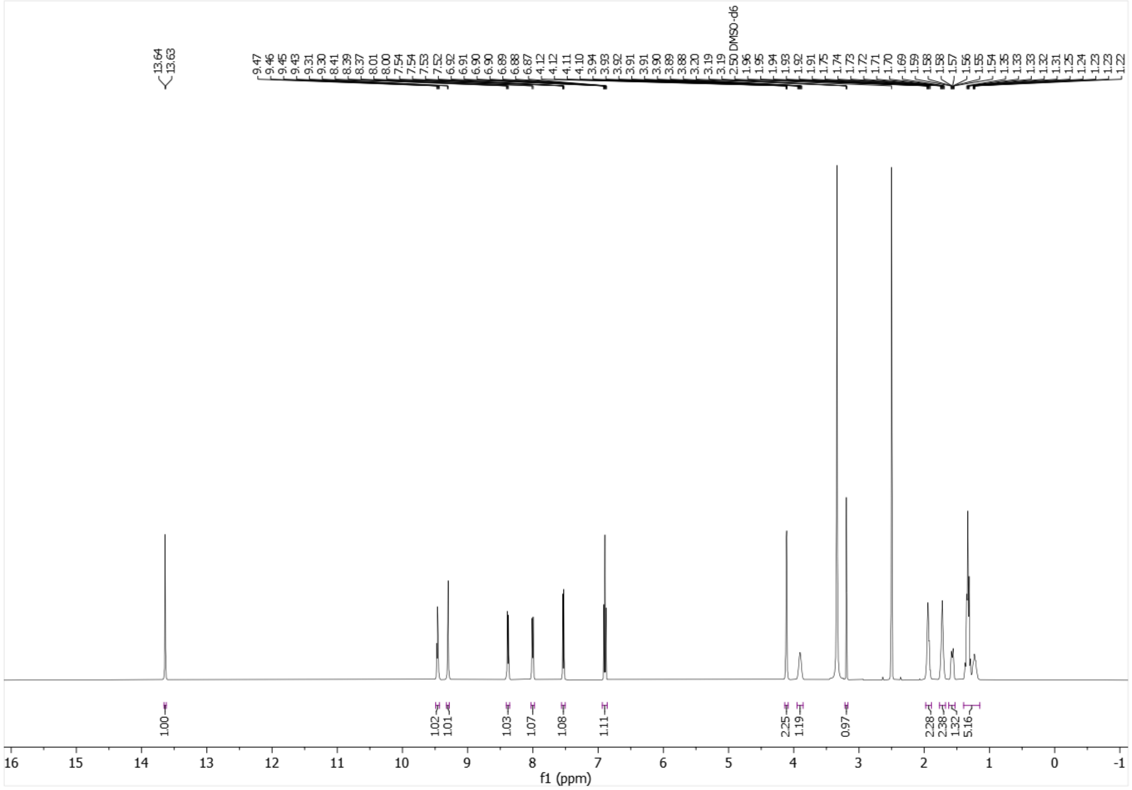

^{1}H-NMR (500 MHz, DMSO-d_6) of **21d**.

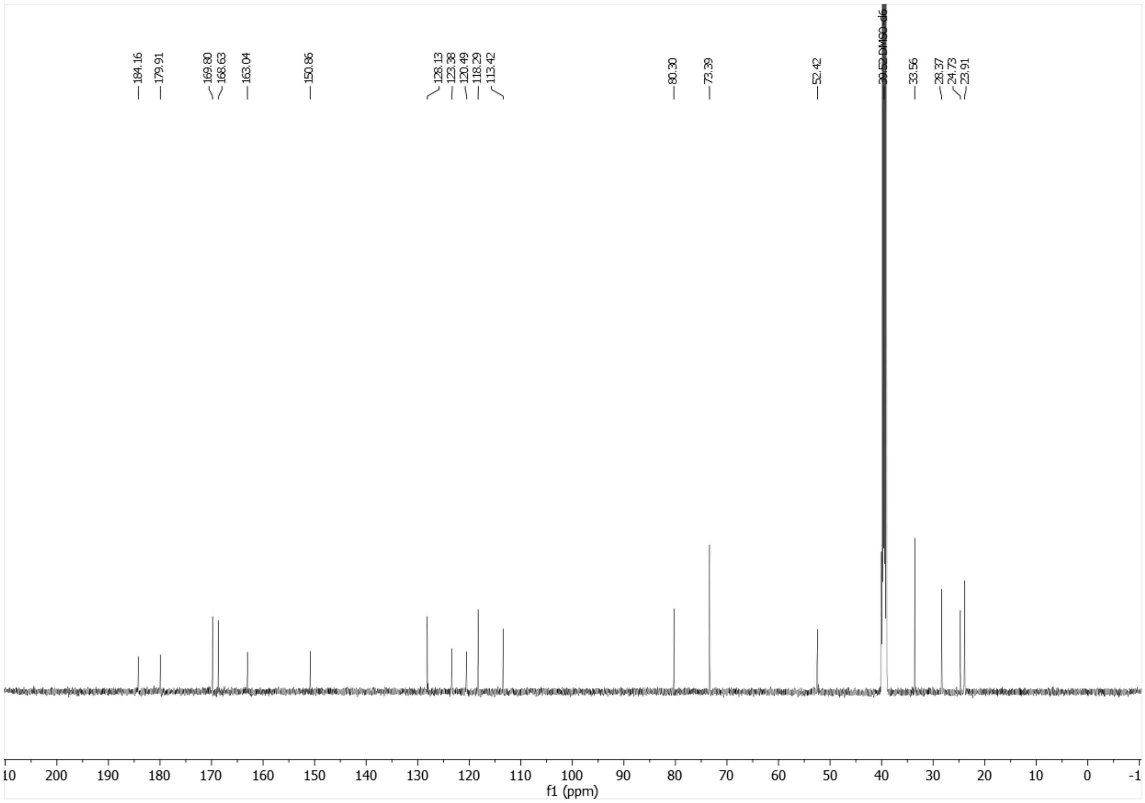

^{13}C-NMR (126 MHz, DMSO-d_6) of **21d**.

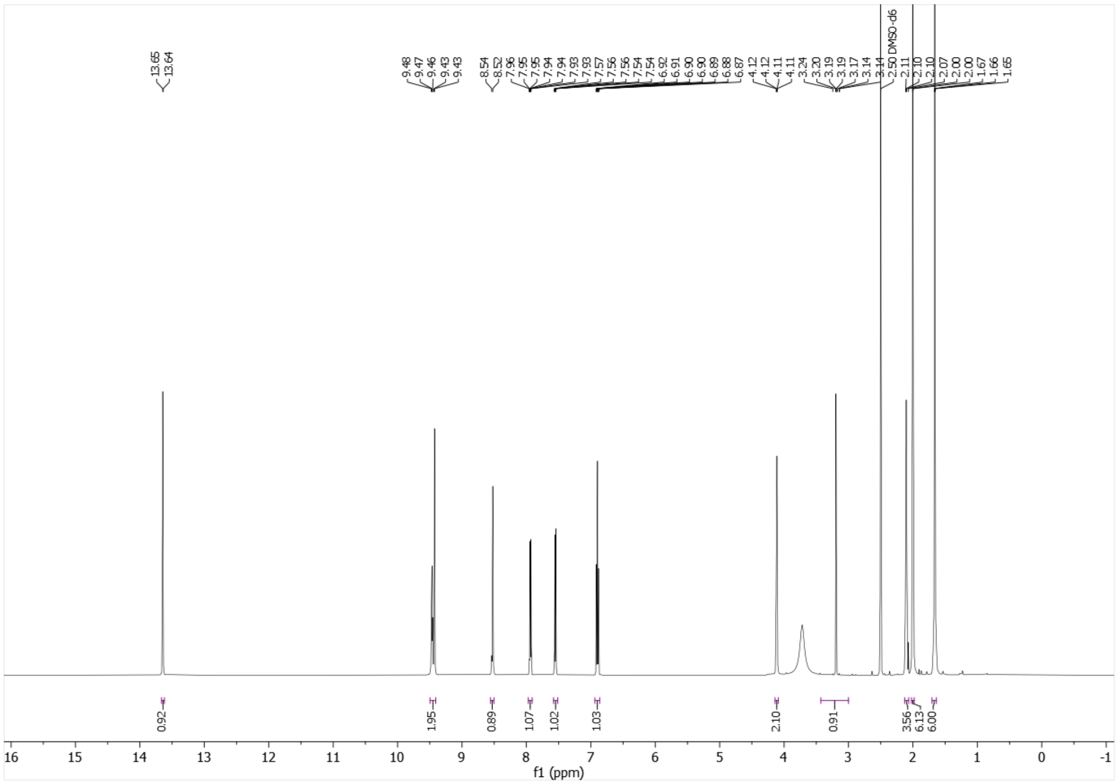

^{1}H-NMR (500 MHz, DMSO-d_6) of **21e**.

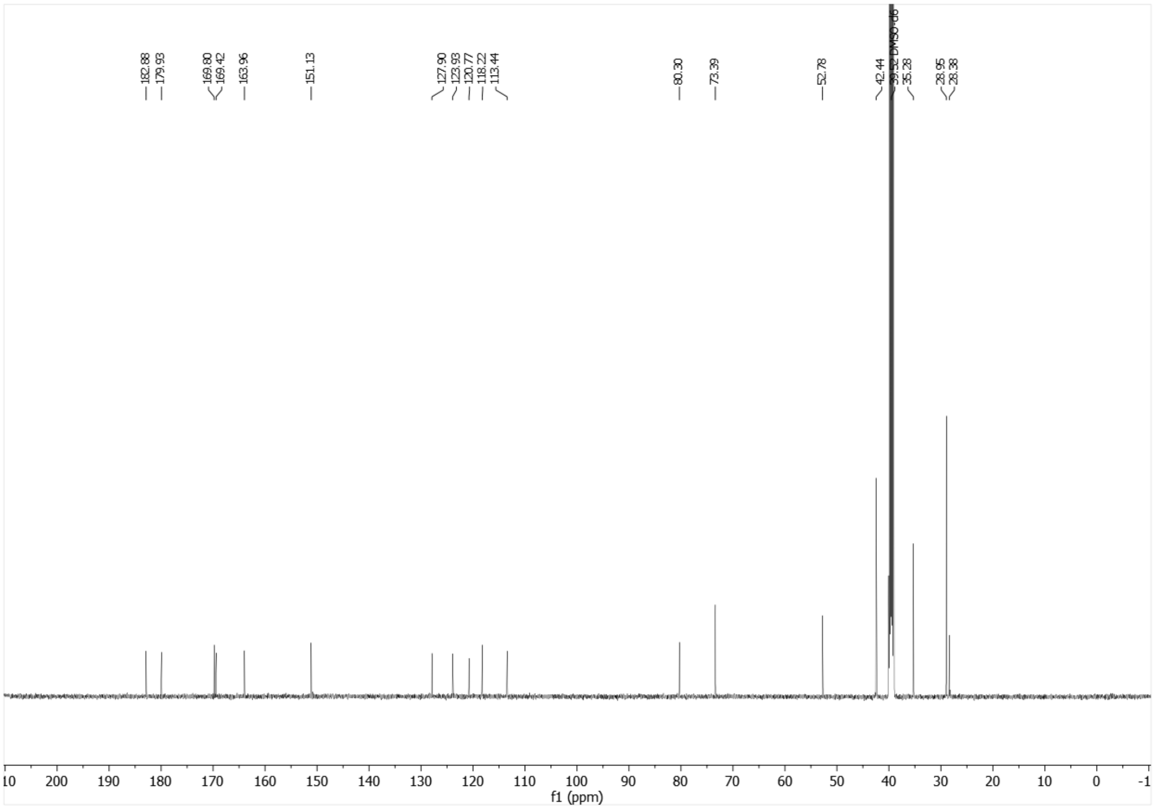

^{13}C-NMR (126 MHz, DMSO-d_6) of **21e**.

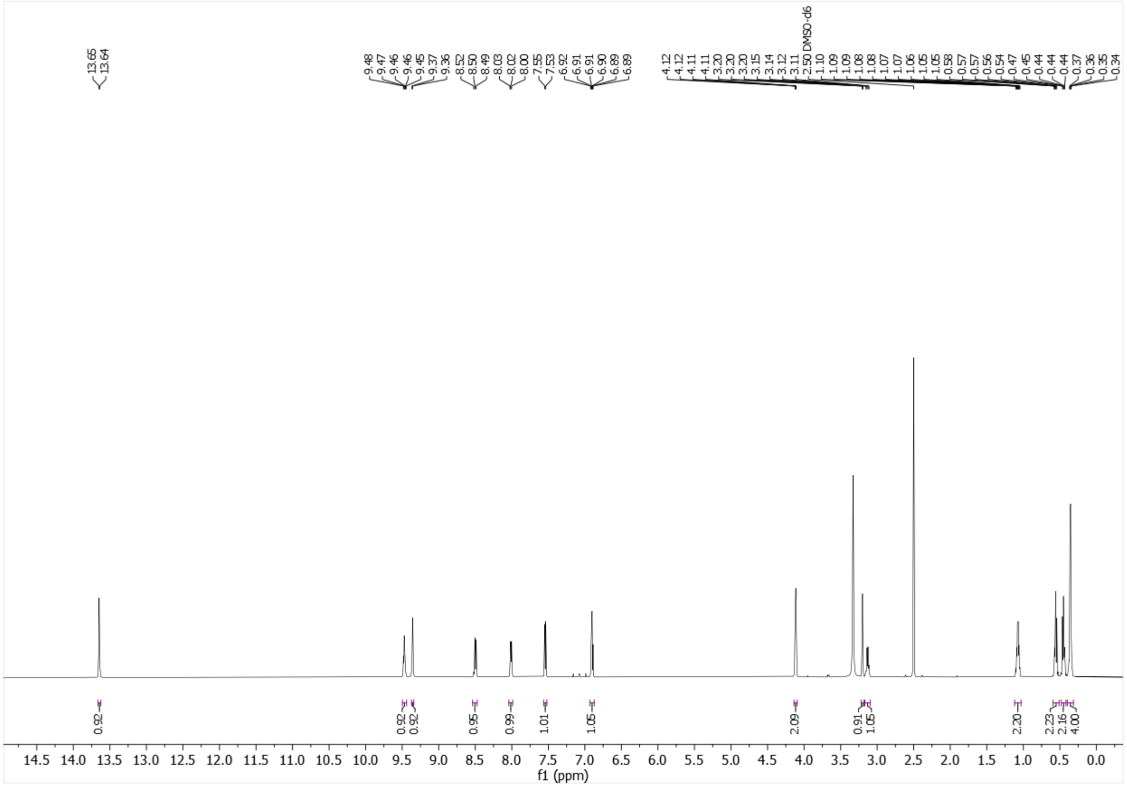

^{1}H-NMR (500 MHz, DMSO-d_6) of **21f**.

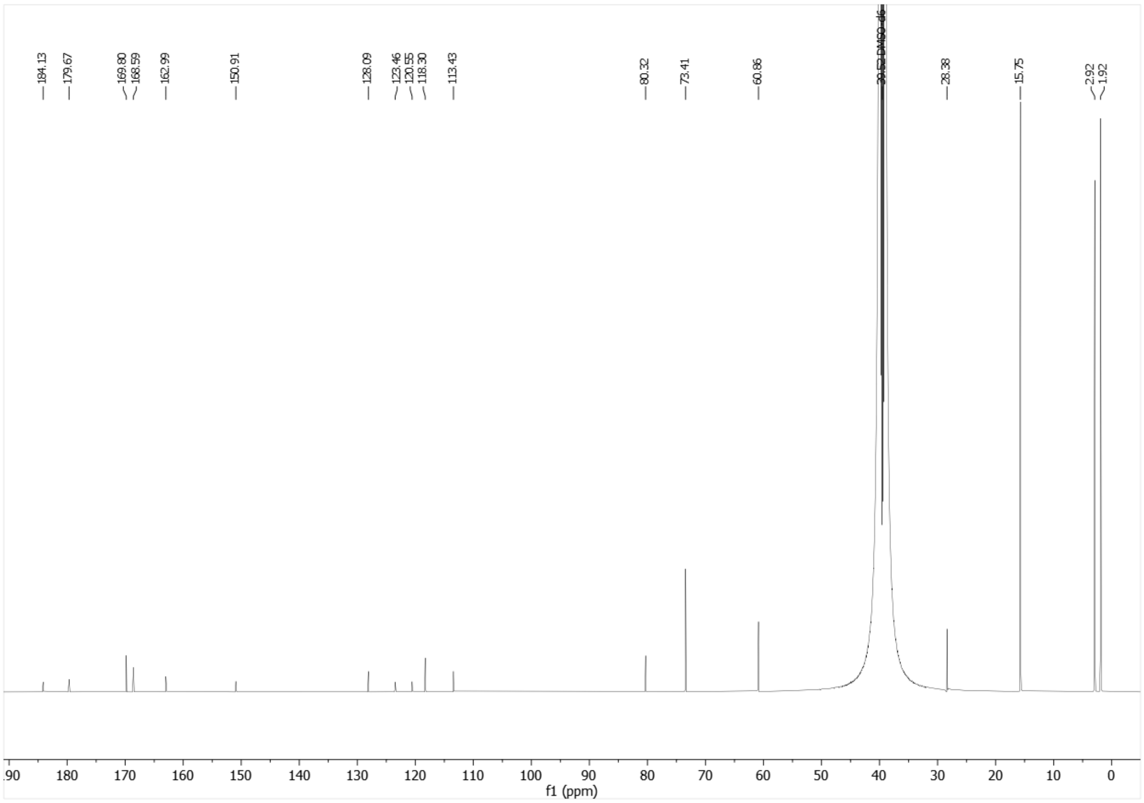

^{13}C-NMR (126 MHz, DMSO-d_6) of **21f**.

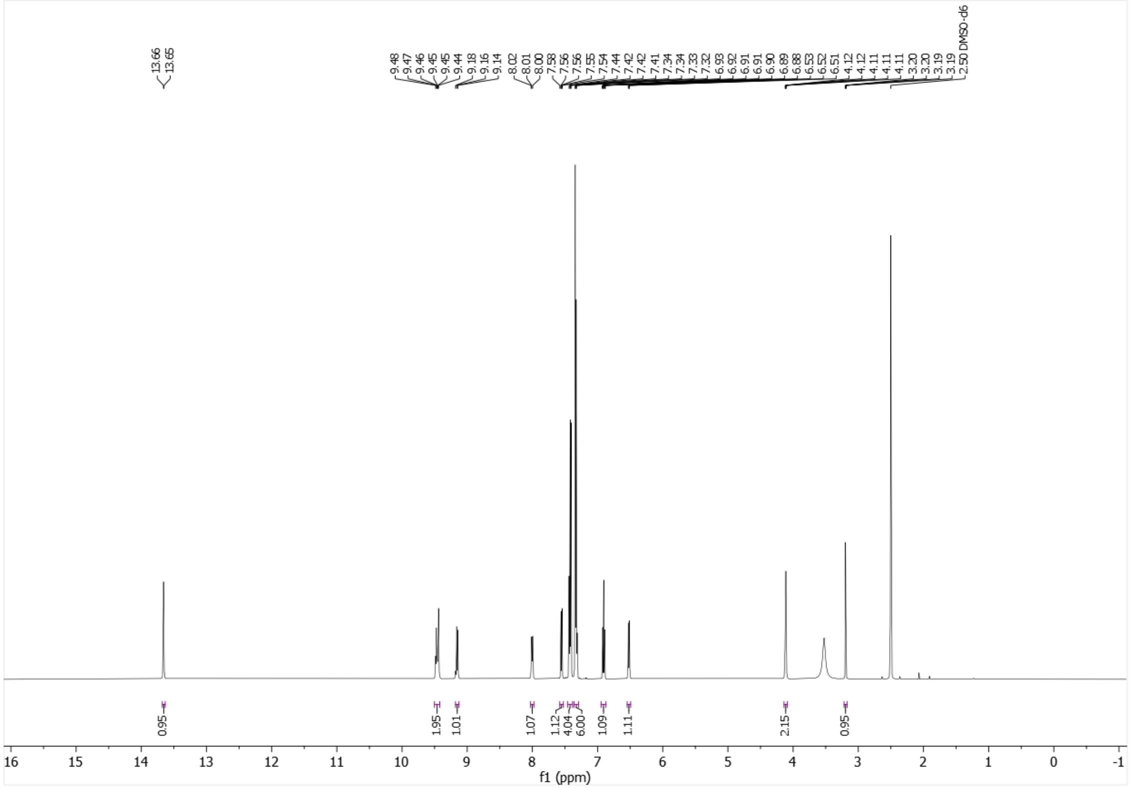

^{1}H-NMR (500 MHz, DMSO-d_6) of **21g**.

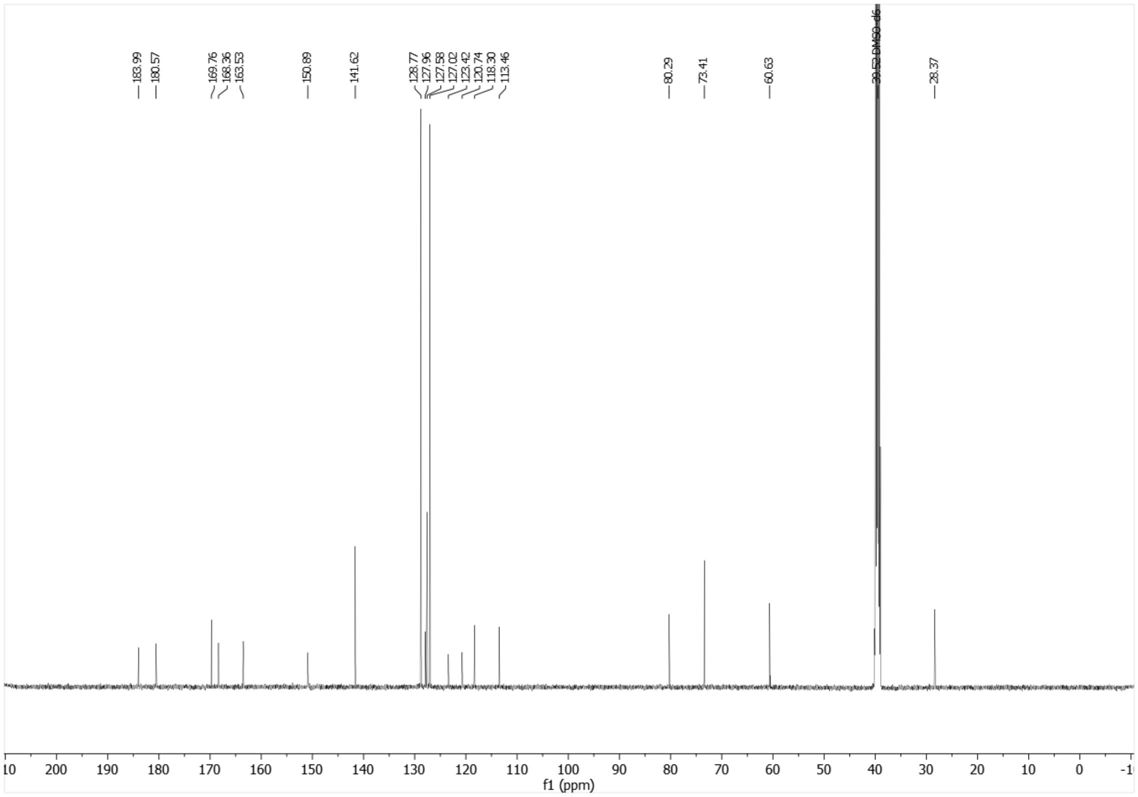

^{13}C-NMR (126 MHz, DMSO-d_6) of **21g**.

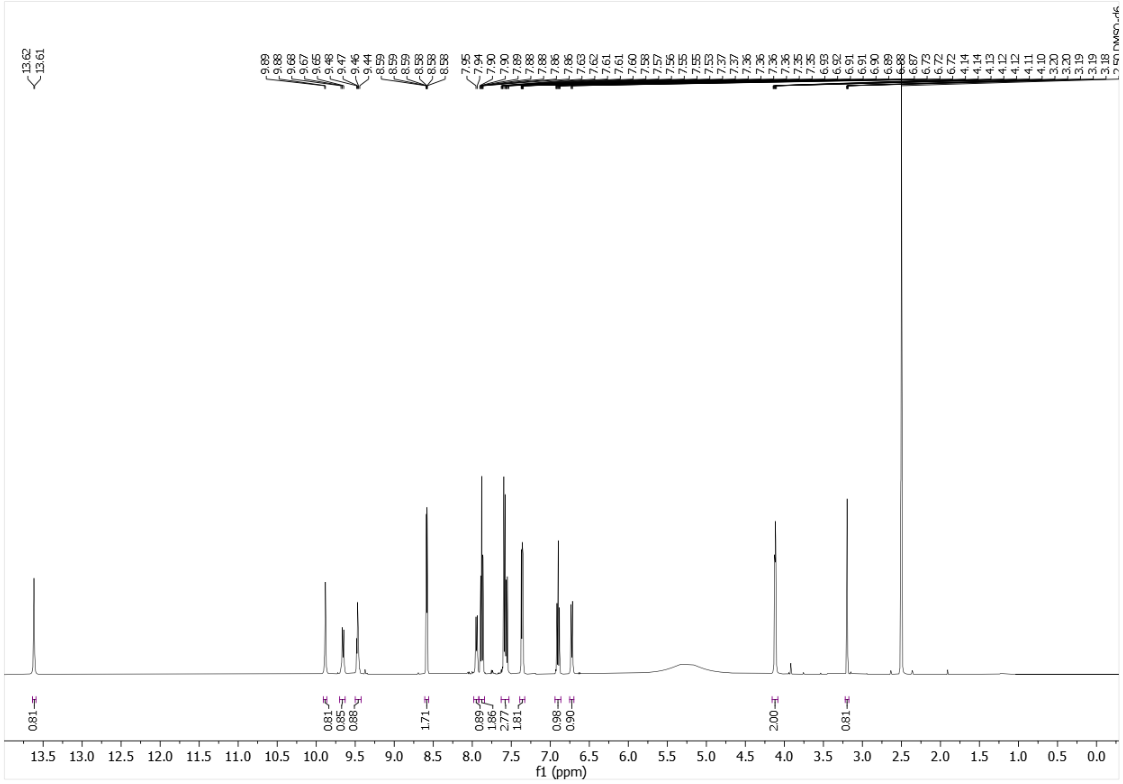

^{1}H-NMR (500 MHz, DMSO-d_6) of **21h**.

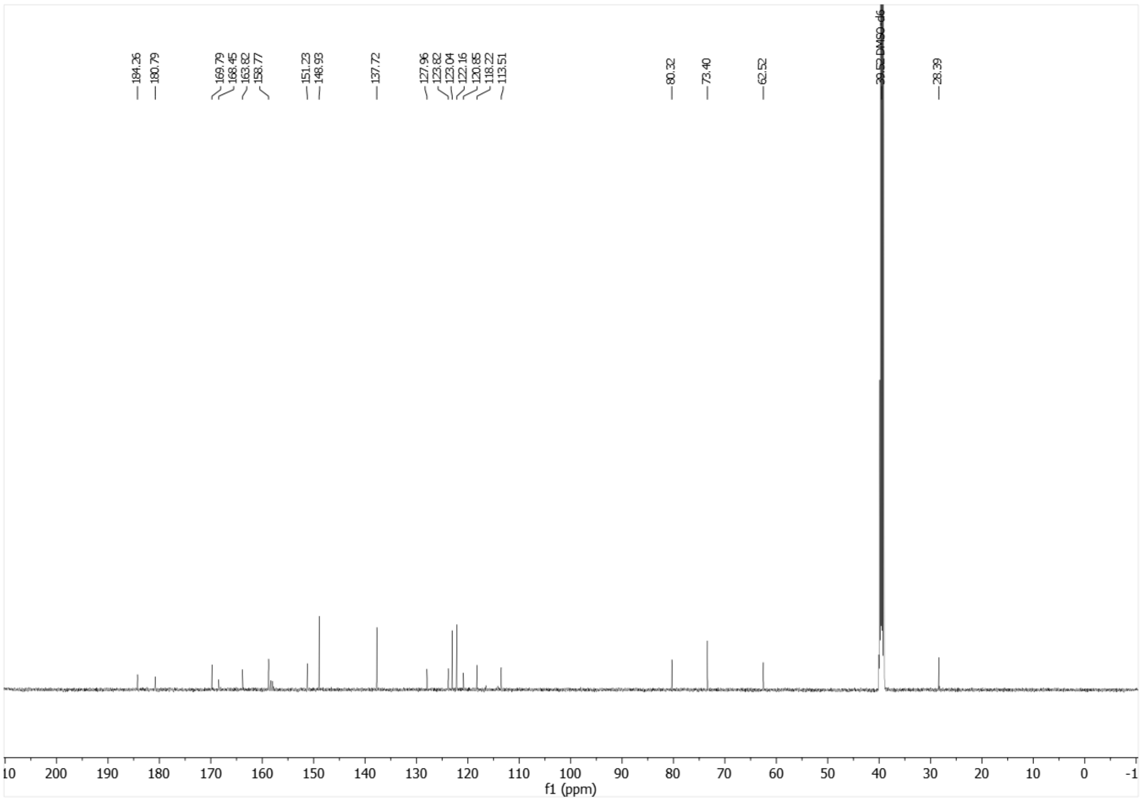

^{13}C-NMR (126 MHz, DMSO-d_6) of **21h**.

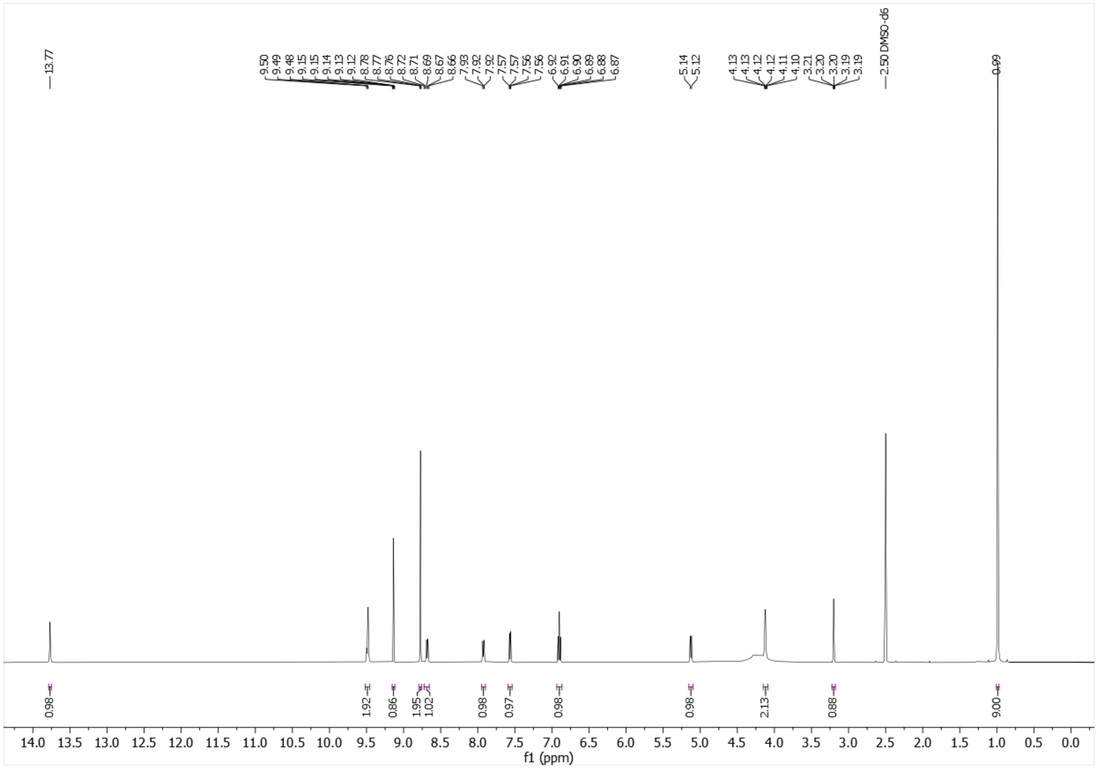

^{1}H-NMR (500 MHz, DMSO-d_6) of **21i**.

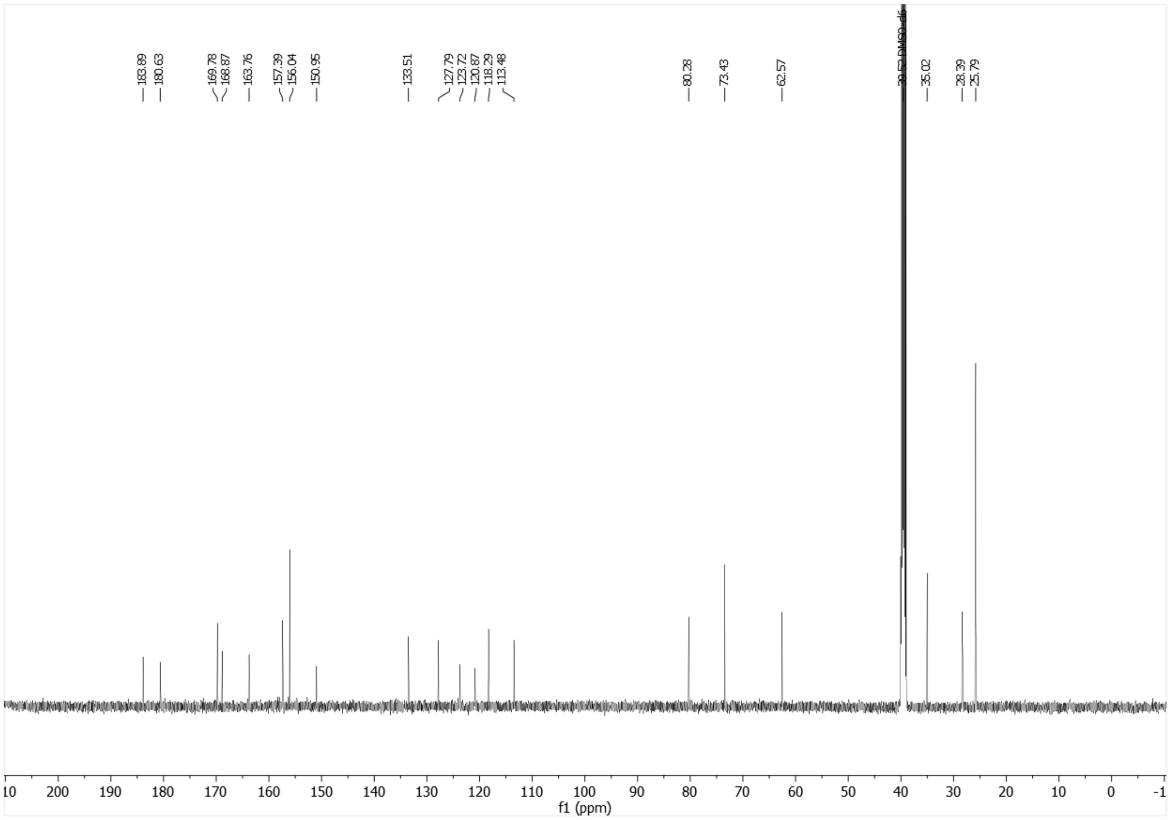

^{13}C-NMR (126 MHz, DMSO-d_6) of **21i**.

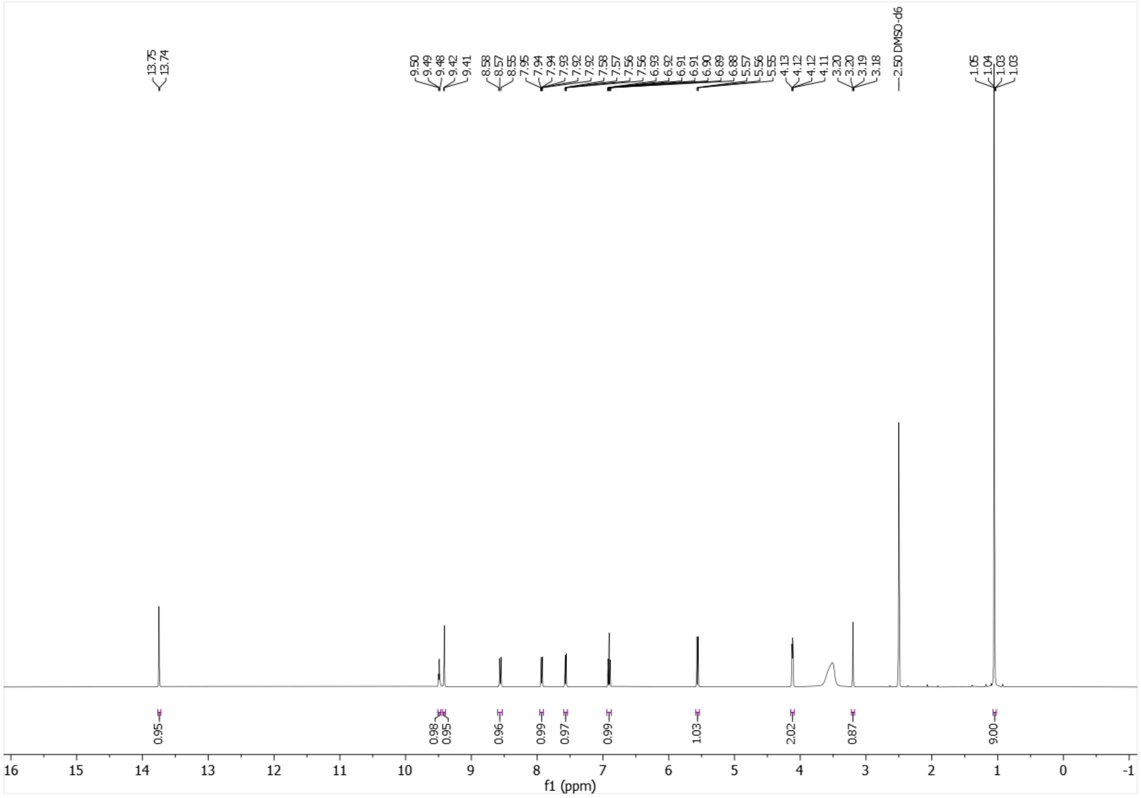

^{1}H-NMR (500 MHz, DMSO-d_6) of **21j**.

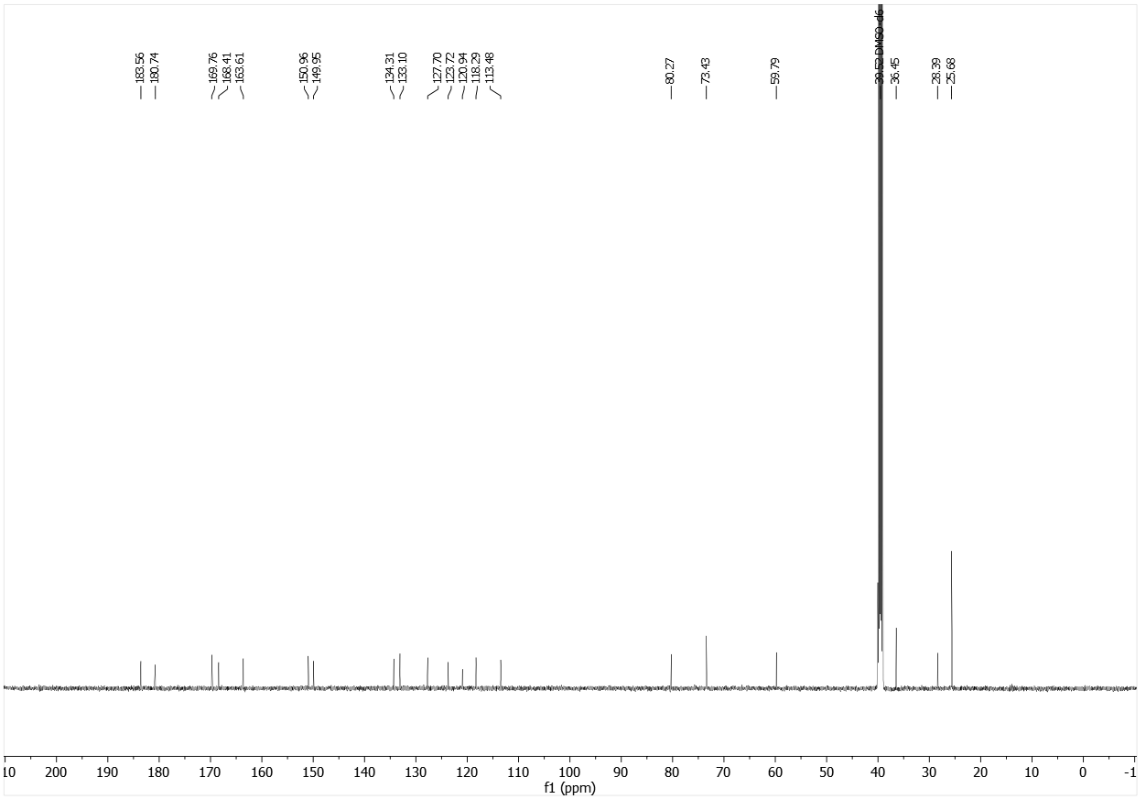

^{13}C-NMR (126 MHz, DMSO-d_6) of **21j**.

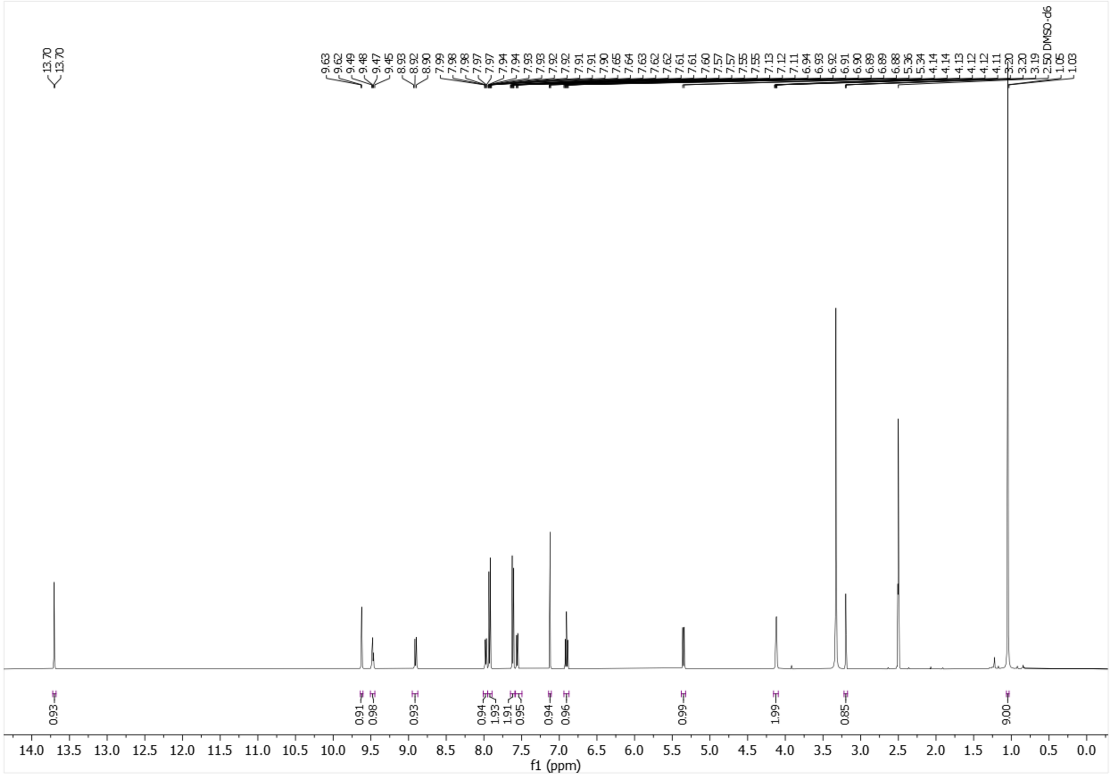

^{1}H-NMR (500 MHz, DMSO-d_6) of **21k**.

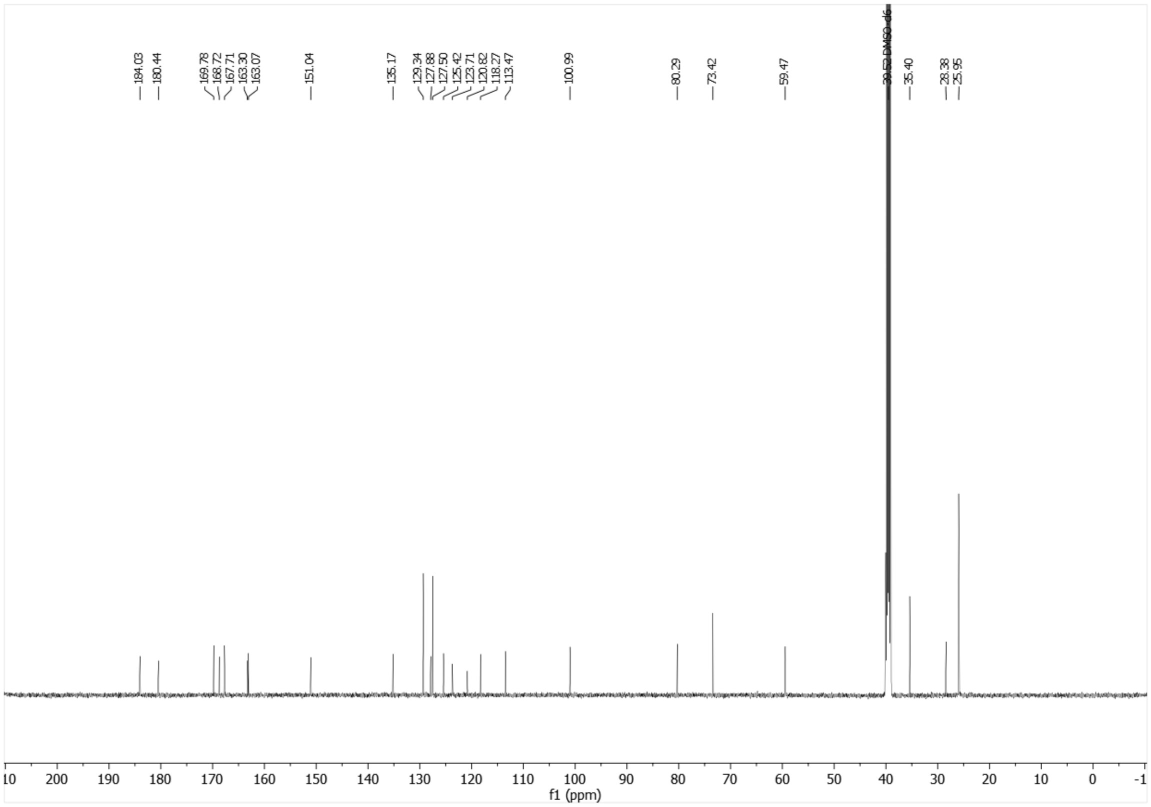

^{13}C-NMR (126 MHz, DMSO-d_6) of **21k**.

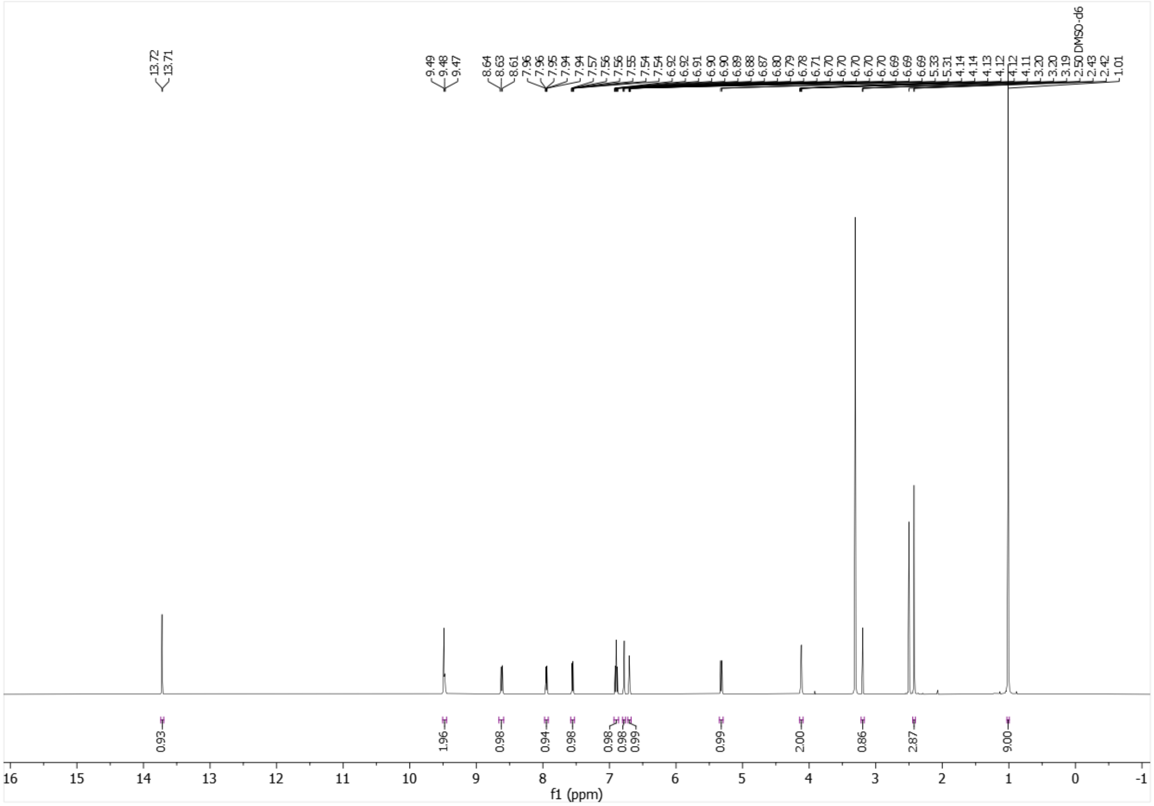

^{1}H-NMR (500 MHz, DMSO-d_6) of **21l**.

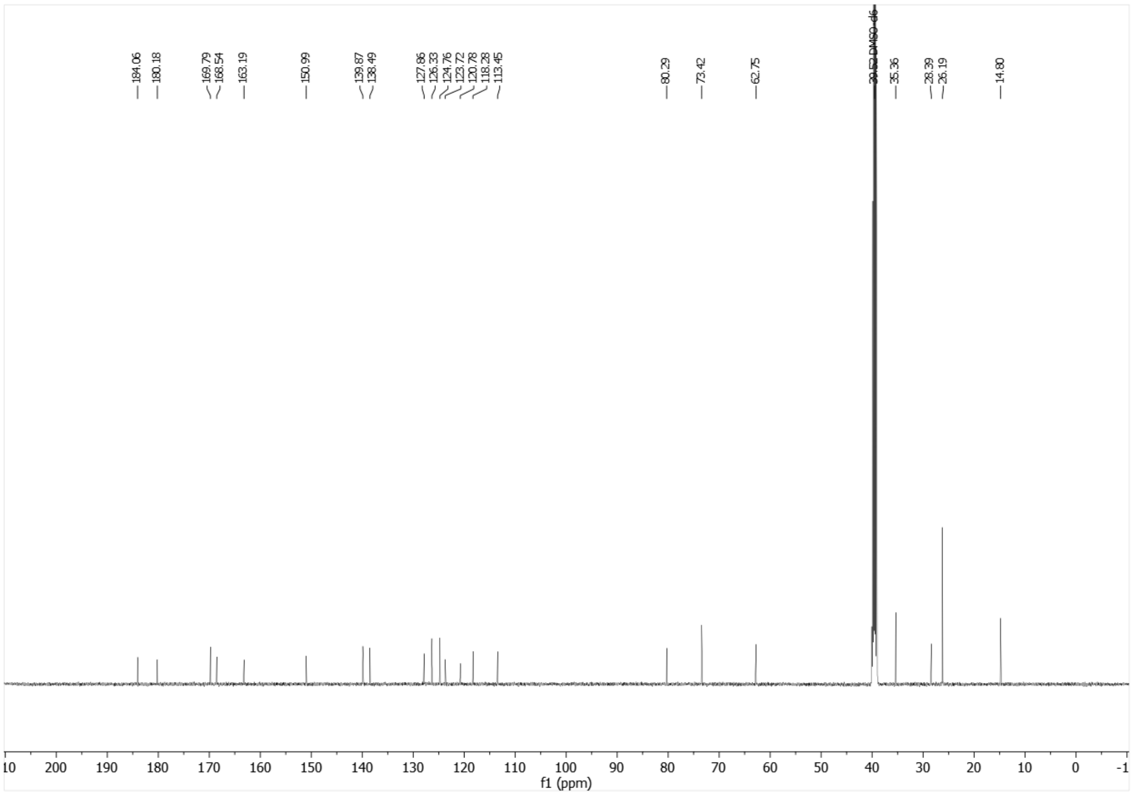

^{13}C-NMR (126 MHz, DMSO-d_6) of **21l**.

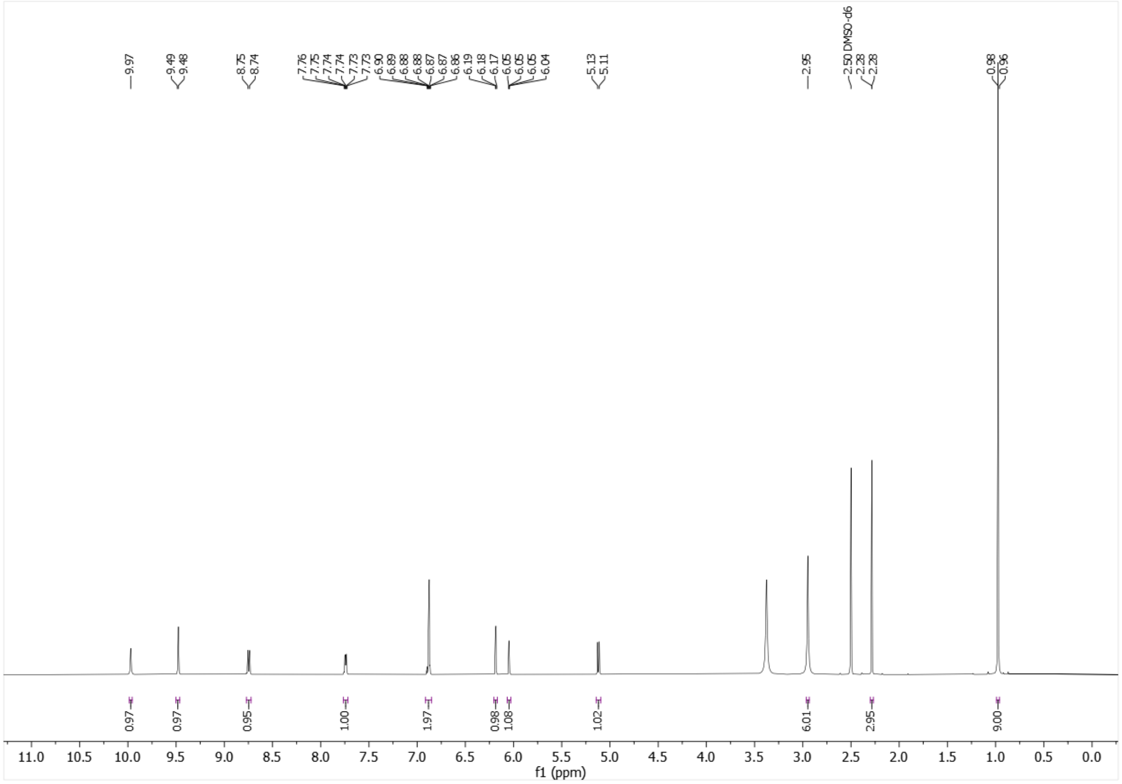

^{1}H-NMR (400 MHz, DMSO-d_6) of SLW132 (**21m**).

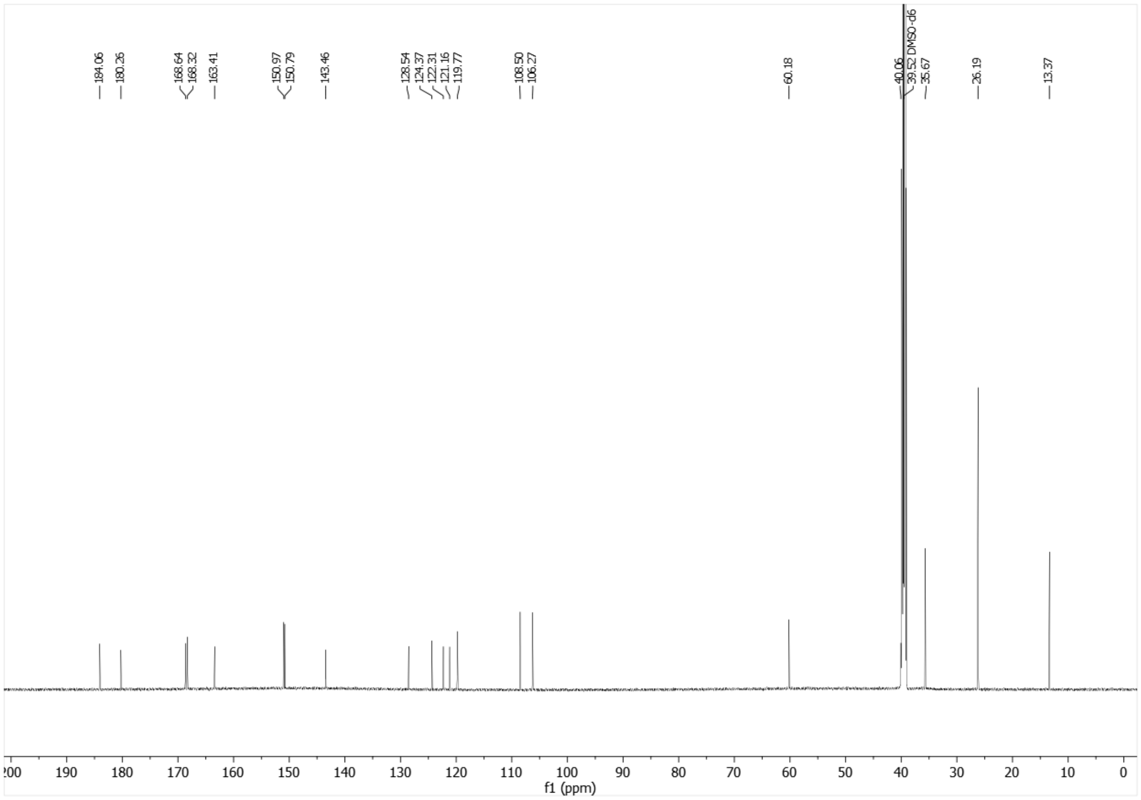

^{13}C-NMR (151 MHz, DMSO-d_6) of SLW132 (**21m**).

HPLC CHROMATOGRAMS OF TARGET COMPOUNDS

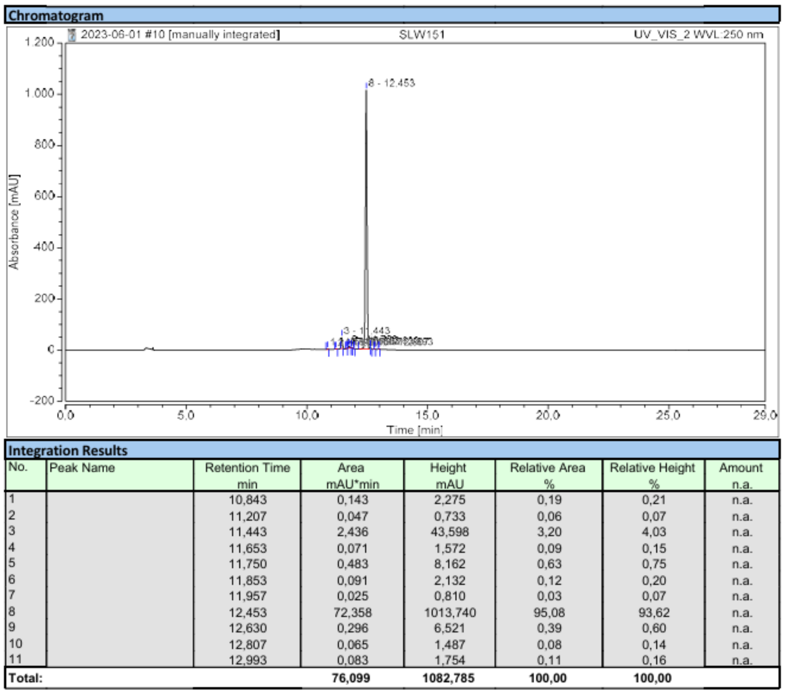

Integration Results

No.	Peak Name	Retention Time min	Area mAU*min	Height mAU	Relative Area %	Relative Height %	Amount n.a.
1		10,843	0,143	2,275	0,19	0,21	n.a.
2		11,207	0,047	0,733	0,06	0,07	n.a.
3		11,443	2,436	43,598	3,20	4,03	n.a.
4		11,653	0,071	1,572	0,09	0,15	n.a.
5		11,750	0,483	8,162	0,63	0,75	n.a.
6		11,853	0,091	2,132	0,12	0,20	n.a.
7		11,957	0,025	0,810	0,03	0,07	n.a.
8		12,453	72,358	1013,740	95,08	93,62	n.a.
9		12,630	0,296	6,521	0,39	0,60	n.a.
10		12,807	0,065	1,487	0,08	0,14	n.a.
11		12,993	0,083	1,754	0,11	0,16	n.a.
**Total:**			**76,099**	**1082,785**	**100,00**	**100,00**	

HPLC chromatogram of **3**.

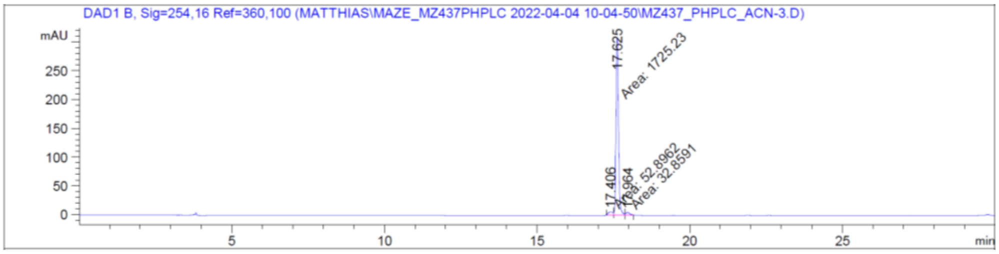


```
=====================================================================
                         Area Percent Report
=====================================================================

Sorted By              :      Signal
Multiplier             :      1.0000
Dilution               :      1.0000
Use Multiplier & Dilution Factor with ISTDs

        Signal 2: DAD1 B, Sig=254,16 Ref=360,100

        Peak RetTime Type  Width     Area      Height     Area
          #   [min]        [min]   [mAU*s]     [mAU]       %
        ----|-------|----|-------|----------|----------|--------|
           1  17.406 MF     0.1777   52.89622    4.96240   2.9209
           2  17.625 MF     0.0929 1725.22974  309.50720  95.2647
           3  17.964 FM     0.1302   32.85914    4.20531   1.8144

        Totals :                   1810.98510  318.67491
```

HPLC chromatogram of Mz437 (**4**).

Chromatogram

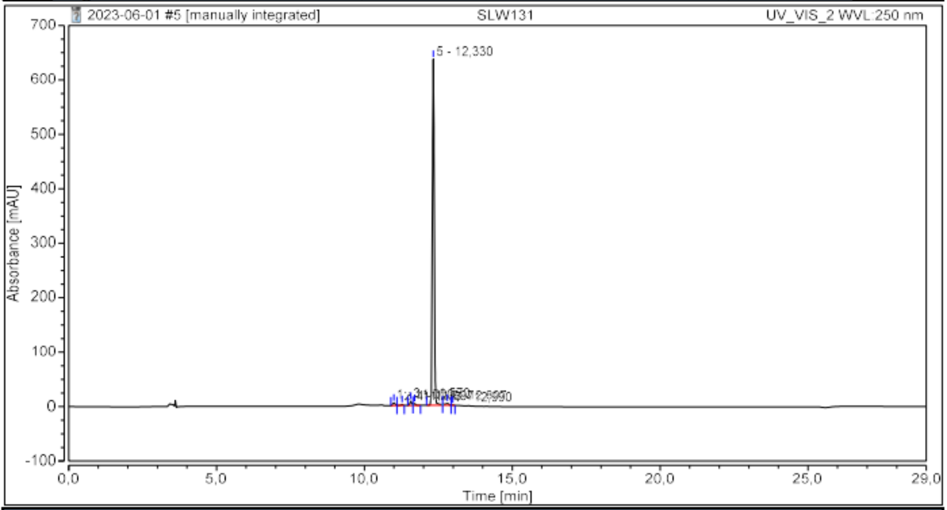

Integration Results

No.	Peak Name	Retention Time min	Area mAU*min	Height mAU	Relative Area %	Relative Height %	Amount n.a.
1		11,000	0,479	5,130	1,01	0,78	n.a.
2		11,273	0,183	2,128	0,39	0,32	n.a.
3		11,570	0,561	7,728	1,19	1,17	n.a.
4		11,697	0,274	3,466	0,58	0,53	n.a.
5		12,330	45,131	635,638	95,55	96,46	n.a.
6		12,807	0,552	4,103	1,17	0,62	n.a.
7		12,990	0,054	0,785	0,11	0,12	n.a.
**Total:**			**47,234**	**658,978**	**100,00**	**100,00**	

HPLC chromatogram of SLW131 (**10**).

Chromatogram

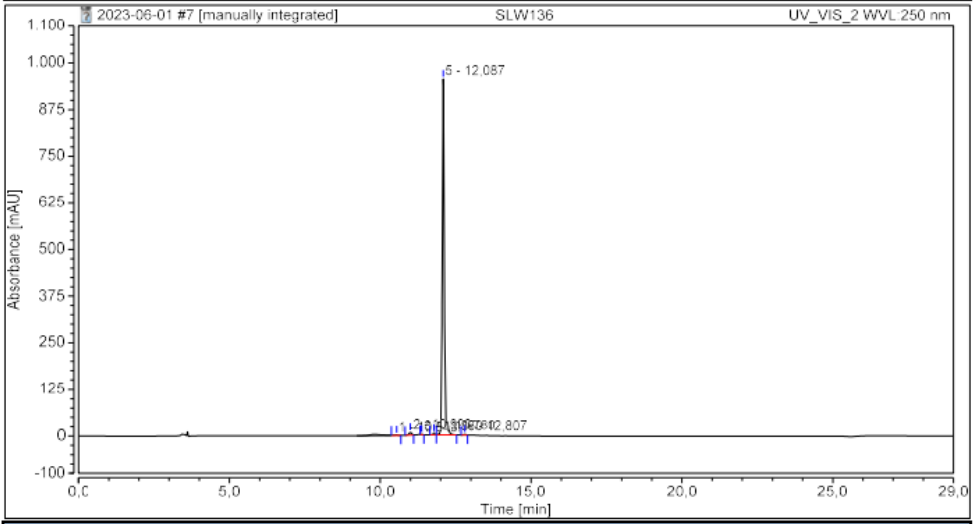

Integration Results

No.	Peak Name	Retention Time min	Area mAU*min	Height mAU	Relative Area %	Relative Height %	Amount n.a.
1		10,543	0,250	1,240	0,33	0,13	n.a.
2		10,993	0,720	7,566	0,95	0,78	n.a.
3		11,370	0,065	1,023	0,09	0,11	n.a.
4		11,780	0,365	4,639	0,48	0,48	n.a.
5		12,087	74,497	954,262	97,90	98,26	n.a.
6		12,807	0,199	2,448	0,26	0,25	n.a.
**Total:**			**76,096**	**971,177**	**100,00**	**100,00**	

HPLC chromatogram of **20a**.

Chromatogram

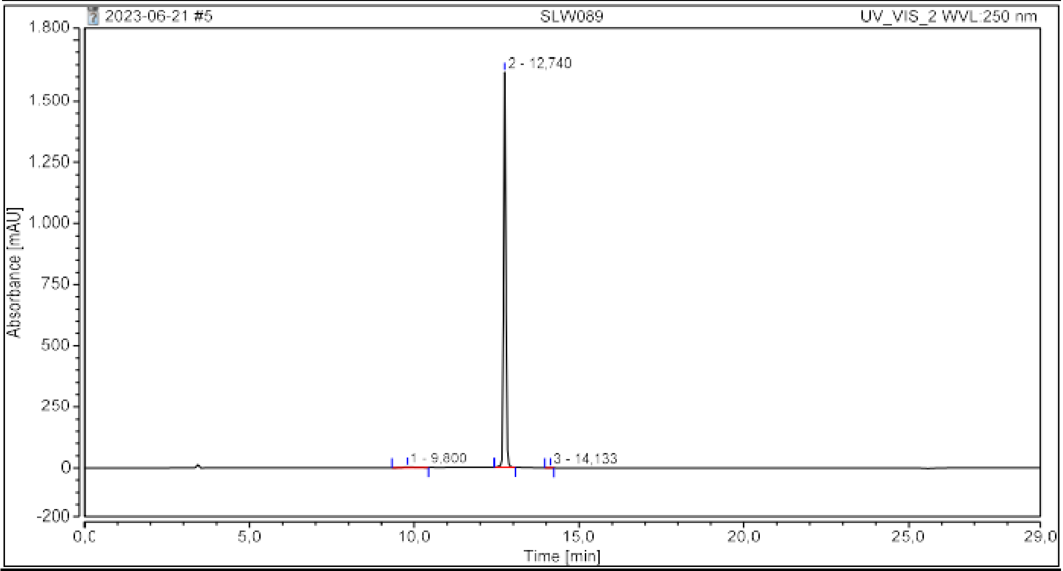

Integration Results

No.	Peak Name	Retention Time min	Area mAU*min	Height mAU	Relative Area %	Relative Height %	Amount n.a.
1		9,800	1,139	2,700	0,86	0,17	n.a.
2		12,740	130,727	1615,520	99,09	99,80	n.a.
3		14,133	0,062	0,604	0,05	0,04	n.a.
**Total:**			**131,928**	**1618,825**	**100,00**	**100,00**	

HPLC chromatogram of **20b**.

Chromatogram

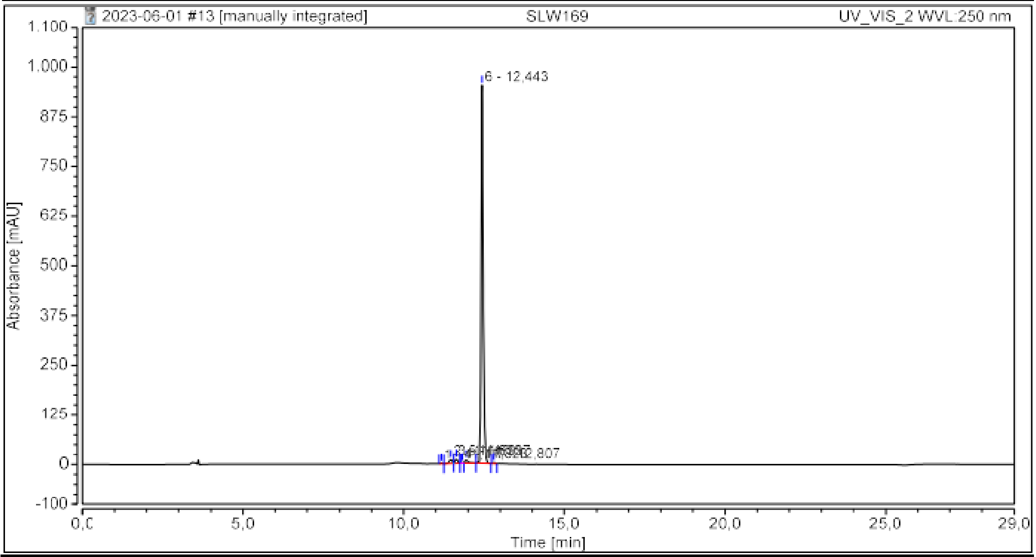

Integration Results

No.	Peak Name	Retention Time min	Area mAU*min	Height mAU	Relative Area %	Relative Height %	Amount n.a.
1		11,177	0,103	1,574	0,14	0,16	n.a.
2		11,470	1,088	10,113	1,50	1,03	n.a.
3		11,633	0,974	10,974	1,34	1,11	n.a.
4		11,823	0,050	1,003	0,07	0,10	n.a.
5		11,937	0,986	9,180	1,36	0,93	n.a.
6		12,443	69,278	951,017	95,36	96,41	n.a.
7		12,807	0,171	2,558	0,24	0,26	n.a.
**Total:**			**72,651**	**986,418**	**100,00**	**100,00**	

HPLC chromatogram of **20c**.

Chromatogram

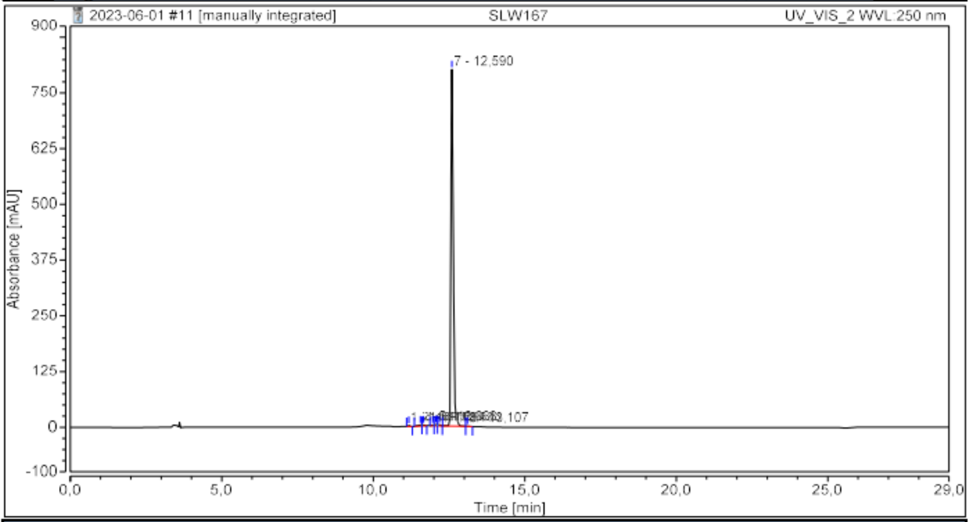

Integration Results

No.	Peak Name	Retention Time min	Area mAU*min	Height mAU	Relative Area %	Relative Height %	Amount n.a.
1		11,177	0,174	2,530	0,24	0,31	n.a.
2		11,553	0,363	2,774	0,50	0,34	n.a.
3		11,657	0,227	2,368	0,31	0,29	n.a.
4		11,963	0,146	2,035	0,20	0,25	n.a.
5		12,063	0,263	3,172	0,36	0,39	n.a.
6		12,173	0,270	2,497	0,37	0,31	n.a.
7		12,590	71,118	799,944	97,92	98,06	n.a.
8		13,107	0,066	0,436	0,09	0,05	n.a.
**Total:**			**72,627**	**815,755**	**100,00**	**100,00**	

HPLC chromatogram of **20d**.

Chromatogram

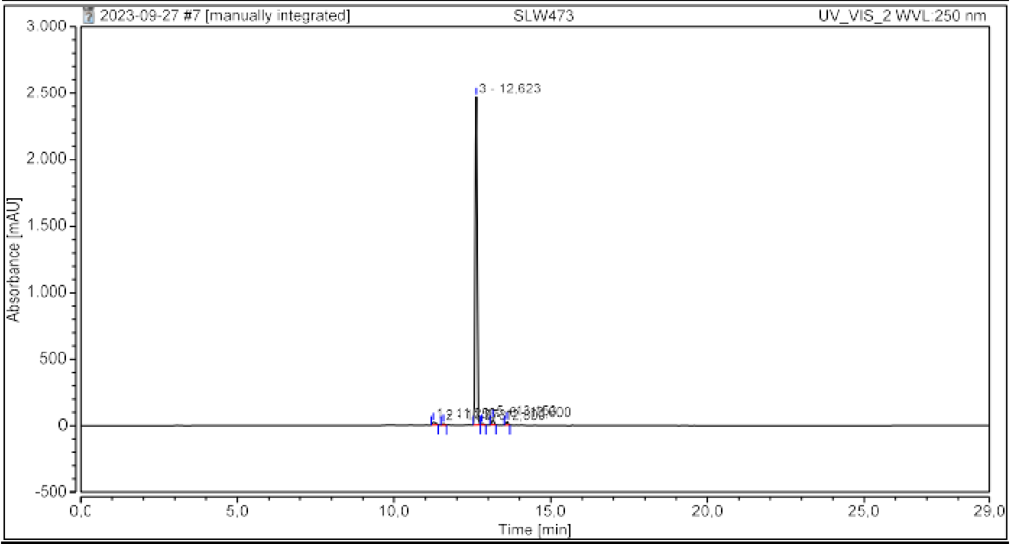

Integration Results

No.	Peak Name	Retention Time min	Area mAU*min	Height mAU	Relative Area %	Relative Height %	Amount n.a.
1		11,253	2,777	23,428	1,53	0,91	n.a.
2		11,573	0,743	10,712	0,41	0,41	n.a.
3		12,623	172,521	2466,286	95,02	95,53	n.a.
4		12,800	0,793	8,690	0,44	0,34	n.a.
5		13,153	2,824	44,217	1,56	1,71	n.a.
6		13,600	1,895	28,325	1,04	1,10	n.a.
**Total:**			**181,554**	**2581,658**	**100,00**	**100,00**	

HPLC chromatogram of **21a**.

Chromatogram

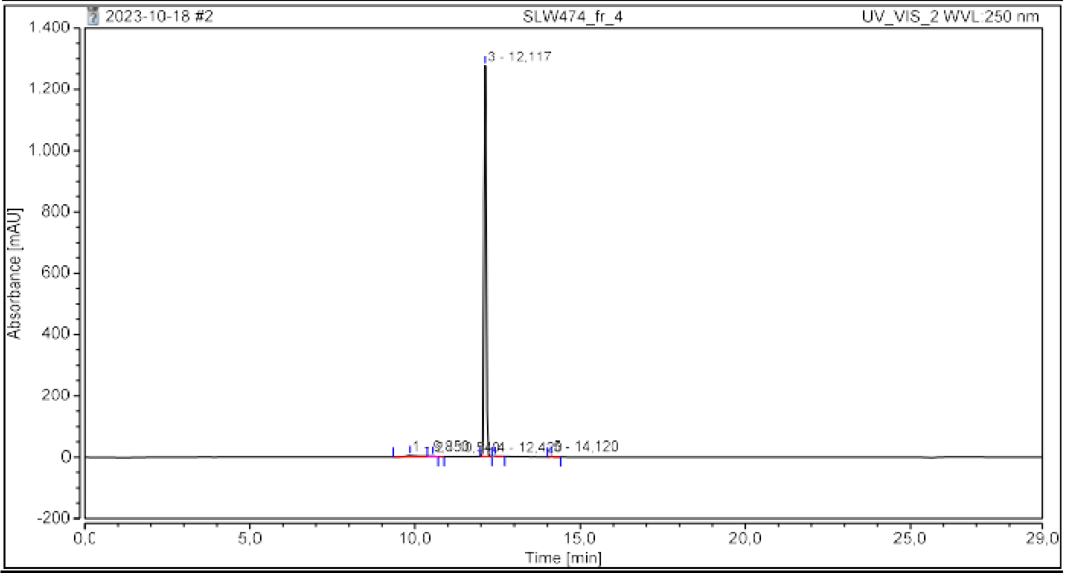

Integration Results

No.	Peak Name	Retention Time min	Area mAU*min	Height mAU	Relative Area %	Relative Height %	Amount n.a.
1		9,850	2,472	4,404	2,65	0,34	n.a.
2		10,540	0,127	0,831	0,14	0,06	n.a.
3		12,117	90,371	1274,775	96,79	99,30	n.a.
4		12,420	0,147	0,825	0,16	0,06	n.a.
5		14,120	0,249	2,912	0,27	0,23	n.a.
**Total:**			**93,367**	**1283,747**	**100,00**	**100,00**	

HPLC chromatogram of **21b**.

Chromatogram

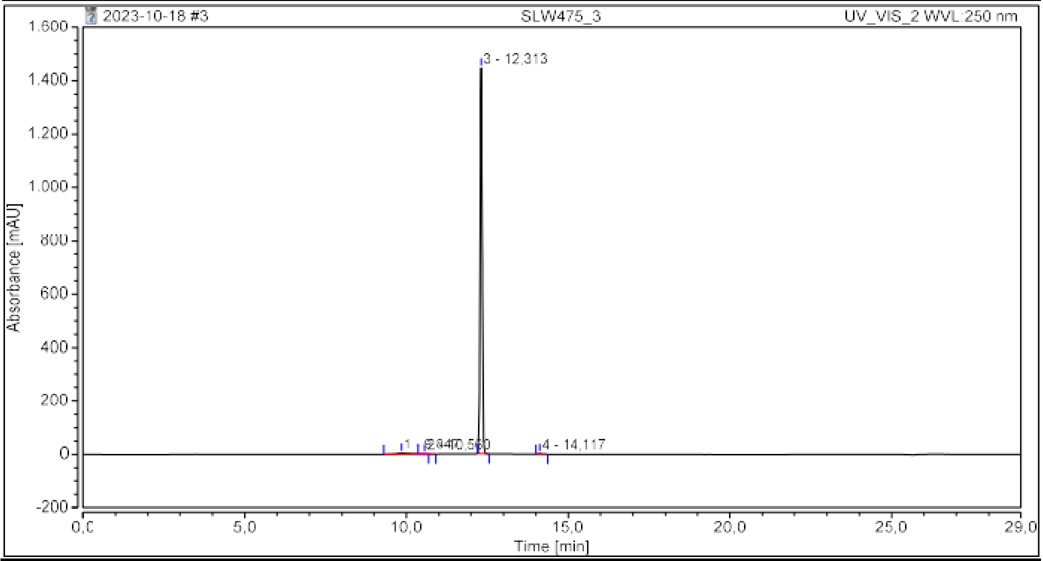

Integration Results

No.	Peak Name	Retention Time min	Area mAU*min	Height mAU	Relative Area %	Relative Height %	Amount n.a.
1		9,847	2,464	4,405	2,32	0,30	n.a.
2		10,560	0,147	0,915	0,14	0,06	n.a.
3		12,313	103,387	1444,231	97,30	99,41	n.a.
4		14,117	0,262	3,245	0,25	0,22	n.a.
**Total:**			**106,260**	**1452,796**	**100,00**	**100,00**	

HPLC chromatogram of **21c**.

Chromatogram

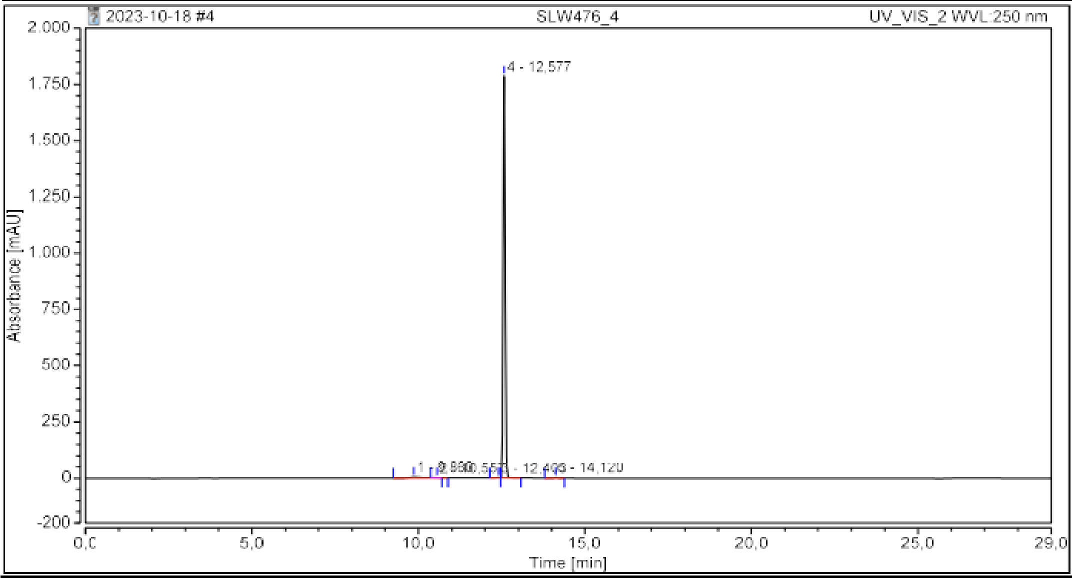

Integration Results

No.	Peak Name	Retention Time<br>min	Area<br>mAU*min	Height<br>mAU	Relative Area<br>%	Relative Height<br>%	Amount<br>n.a.
1		9,860	2,497	4,405	1,89	0,25	n.a.
2		10,557	0,132	0,826	0,10	0,05	n.a.
3		12,403	0,196	1,881	0,15	0,10	n.a.
4		12,577	129,293	1785,790	97,60	99,41	n.a.
5		14,120	0,348	3,439	0,26	0,19	n.a.
**Total:**			**132,466**	**1796,340**	**100,00**	**100,00**	

HPLC chromatogram of **21d**.

Chromatogram

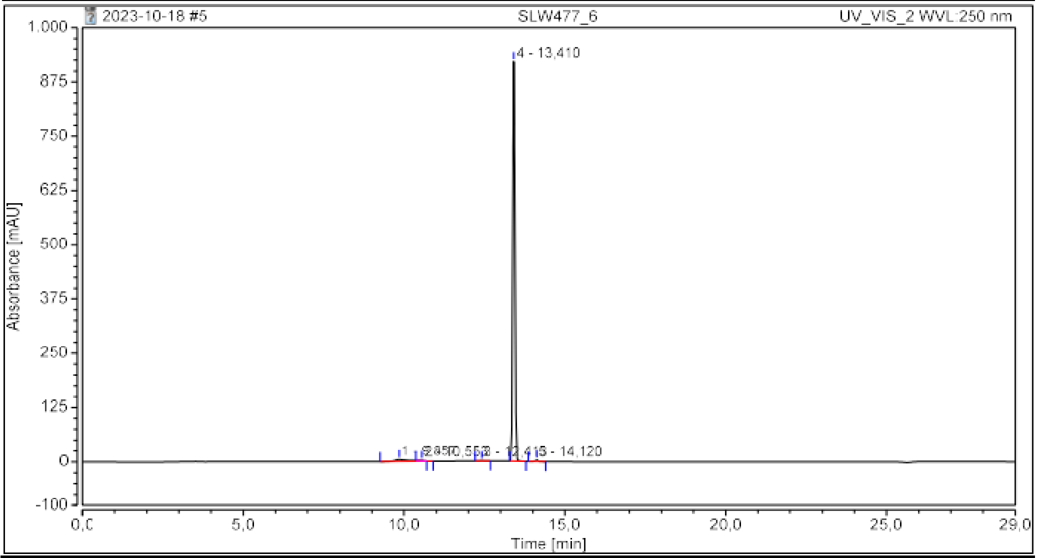

Integration Results

No.	Peak Name	Retention Time<br>min	Area<br>mAU*min	Height<br>mAU	Relative Area<br>%	Relative Height<br>%	Amount<br>n.a.
1		9,857	2,494	4,483	3,35	0,48	n.a.
2		10,553	0,141	0,914	0,19	0,10	n.a.
3		12,413	0,100	0,565	0,13	0,06	n.a.
4		13,410	71,404	919,660	95,88	98,99	n.a.
5		14,120	0,334	3,415	0,45	0,37	n.a.
**Total:**			**74,472**	**929,037**	**100,00**	**100,00**	

HPLC chromatogram of **21e**.

Chromatogram

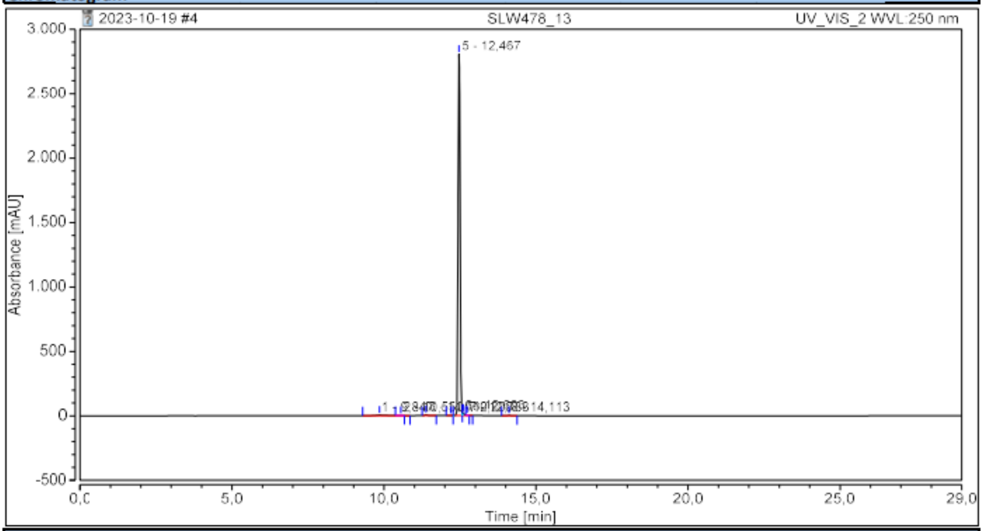

Integration Results

No.	Peak Name	Retention Time min	Area mAU*min	Height mAU	Relative Area %	Relative Height %	Amount n.a.
1		9,847	2,447	4,516	1,15	0,16	n.a.
2		10,550	0,109	0,746	0,05	0,03	n.a.
3		11,380	0,808	7,159	0,38	0,25	n.a.
4		12,200	0,110	1,136	0,05	0,04	n.a.
5		12,467	206,955	2804,777	97,44	98,64	n.a.
6		12,603	1,506	19,157	0,71	0,67	n.a.
7		12,733	0,143	2,391	0,07	0,08	n.a.
8		14,113	0,325	3,476	0,15	0,12	n.a.
**Total:**			**212,402**	**2843,358**	**100,00**	**100,00**	

HPLC chromatogram of **21f**.

Chromatogram

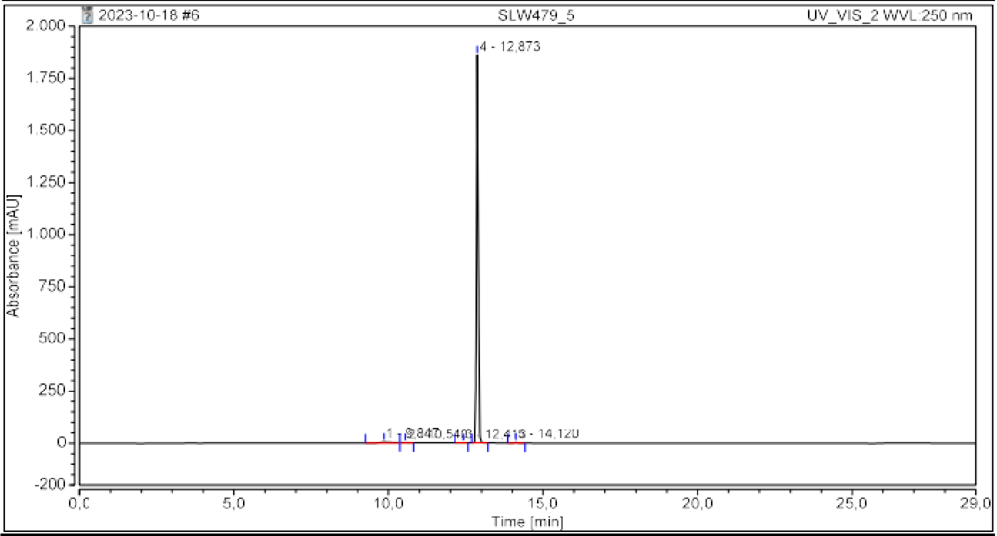

Integration Results

No.	Peak Name	Retention Time min	Area mAU*min	Height mAU	Relative Area %	Relative Height %	Amount n.a.
1		9,847	2,084	4,270	1,59	0,23	n.a.
2		10,540	0,443	1,781	0,34	0,10	n.a.
3		12,413	0,074	0,494	0,06	0,03	n.a.
4		12,873	128,498	1859,417	97,76	99,47	n.a.
5		14,120	0,343	3,438	0,26	0,18	n.a.
**Total:**			**131,442**	**1869,400**	**100,00**	**100,00**	

HPLC chromatogram of **21g**.

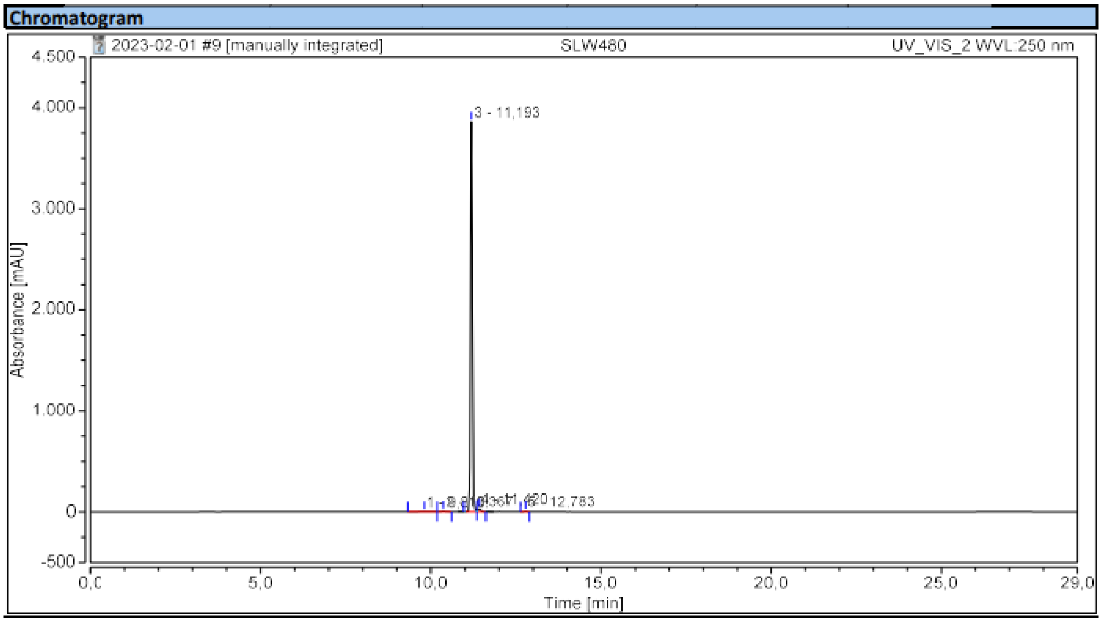

Integration Results

No.	Peak Name	Retention Time<br>min	Area<br>mAU*min	Height<br>mAU	Relative Area<br>%	Relative Height<br>%	Amount<br>n.a.
1		9,810	1,562	3,797	0,57	0,10	n.a.
2		10,367	0,994	6,179	0,36	0,16	n.a.
3		11,193	269,811	3856,641	97,85	98,83	n.a.
4		11,420	2,997	29,965	1,09	0,77	n.a.
5		12,783	0,384	5,681	0,14	0,15	n.a.
**Total:**			**275,748**	**3902,264**	**100,00**	**100,00**	

HPLC chromatogram of **21h**.

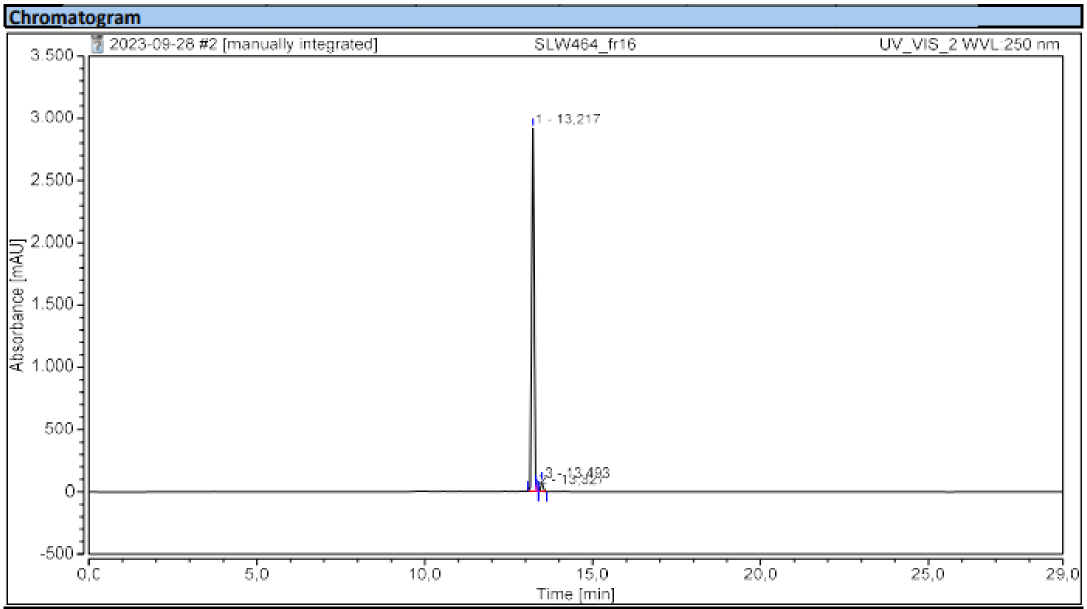

Integration Results

No.	Peak Name	Retention Time<br>min	Area<br>mAU*min	Height<br>mAU	Relative Area<br>%	Relative Height<br>%	Amount<br>n.a.
1		13,217	268,050	2914,265	97,92	97,31	n.a.
2		13,327	0,238	5,846	0,09	0,20	n.a.
3		13,493	5,442	74,692	1,99	2,49	n.a.
**Total:**			**273,730**	**2994,802**	**100,00**	**100,00**	

HPLC chromatogram of **21i**.

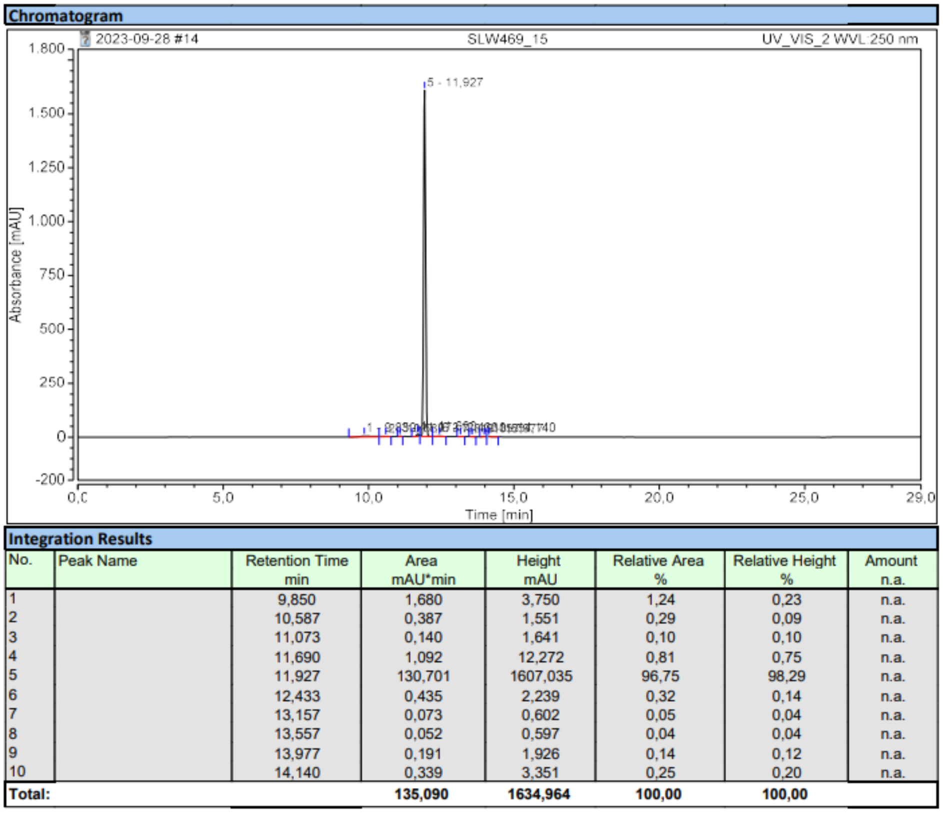

Integration Results

No.	Peak Name	Retention Time min	Area mAU*min	Height mAU	Relative Area %	Relative Height %	Amount n.a.
1		9,850	1,680	3,750	1,24	0,23	n.a.
2		10,587	0,387	1,551	0,29	0,09	n.a.
3		11,073	0,140	1,641	0,10	0,10	n.a.
4		11,690	1,092	12,272	0,81	0,75	n.a.
5		11,927	130,701	1607,035	96,75	98,29	n.a.
6		12,433	0,435	2,239	0,32	0,14	n.a.
7		13,157	0,073	0,602	0,05	0,04	n.a.
8		13,557	0,052	0,597	0,04	0,04	n.a.
9		13,977	0,191	1,926	0,14	0,12	n.a.
10		14,140	0,339	3,351	0,25	0,20	n.a.
**Total:**			**135,090**	**1634,964**	**100,00**	**100,00**	

HPLC chromatogram of **21j**.

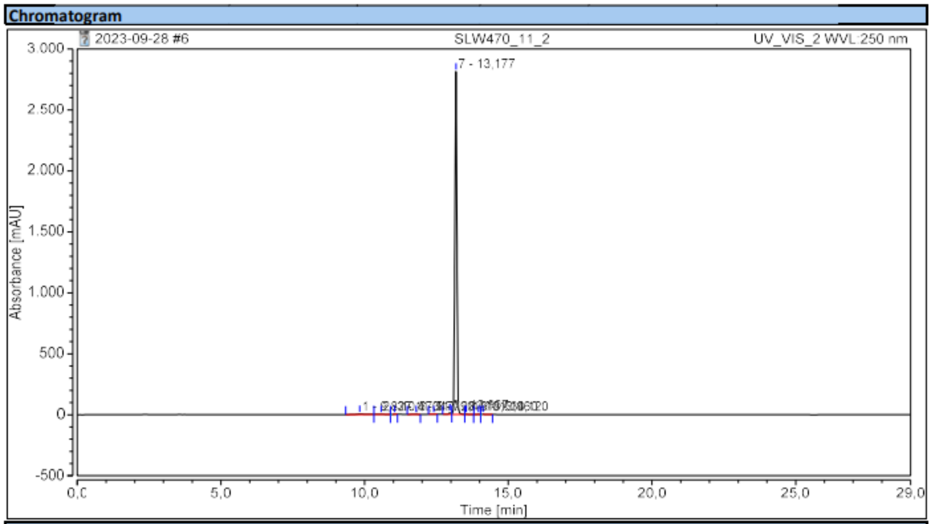

Integration Results

No.	Peak Name	Retention Time min	Area mAU*min	Height mAU	Relative Area %	Relative Height %	Amount n.a.
1		9,827	1,738	3,821	0,70	0,13	n.a.
2		10,573	0,535	1,821	0,22	0,06	n.a.
3		11,047	0,115	1,309	0,05	0,05	n.a.
4		11,790	0,195	1,189	0,08	0,04	n.a.
5		12,407	0,187	1,404	0,08	0,05	n.a.
6		12,967	1,035	15,222	0,42	0,54	n.a.
7		13,177	243,591	2810,983	98,20	98,91	n.a.
8		13,533	0,197	1,340	0,08	0,05	n.a.
9		13,960	0,144	1,376	0,06	0,05	n.a.
10		14,120	0,331	3,505	0,13	0,12	n.a.
**Total:**			**248,067**	**2841,970**	**100,00**	**100,00**	

HPLC chromatogram of **21k**.

Chromatogram

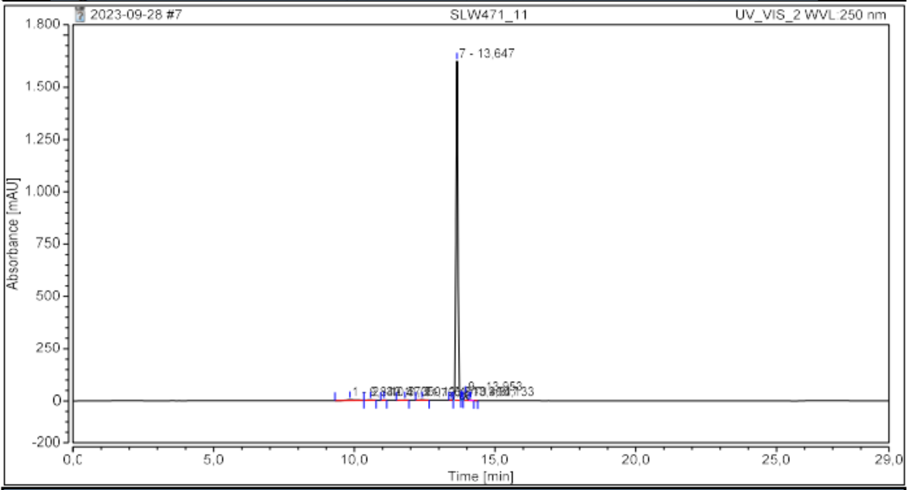

Integration Results

No.	Peak Name	Retention Time min	Area mAU*min	Height mAU	Relative Area %	Relative Height %	Amount n.a.
1		9,840	1,710	3,849	1,26	0,23	n.a.
2		10,573	0,421	1,609	0,31	0,10	n.a.
3		11,050	0,129	1,414	0,10	0,08	n.a.
4		11,793	0,186	1,159	0,14	0,07	n.a.
5		12,407	0,293	1,751	0,22	0,10	n.a.
6		13,460	0,117	1,486	0,09	0,09	n.a.
7		13,647	129,715	1622,722	95,58	97,16	n.a.
8		13,817	0,386	5,375	0,28	0,32	n.a.
9		13,953	2,562	27,954	1,89	1,67	n.a.
10		14,133	0,200	2,838	0,15	0,17	n.a.
**Total:**			**135,720**	**1670,158**	**100,00**	**100,00**	

HPLC chromatogram of **21l**.

Chromatogram

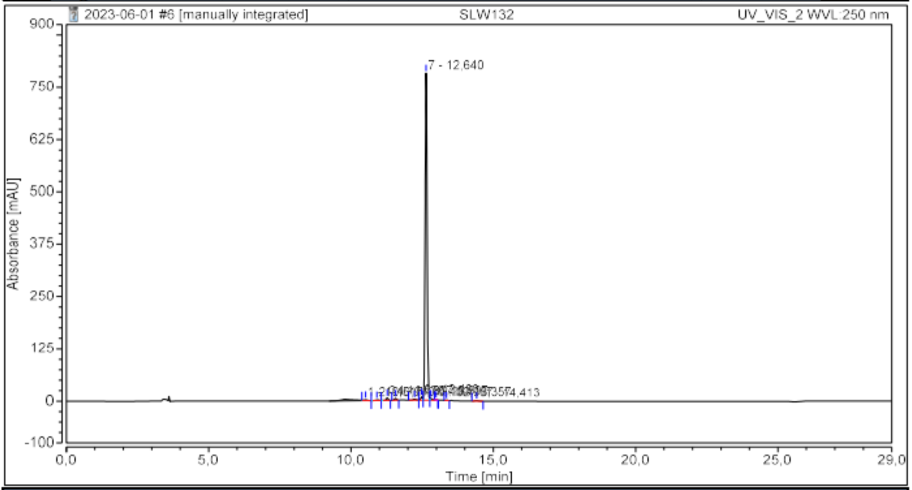

Integration Results

No.	Peak Name	Retention Time min	Area mAU*min	Height mAU	Relative Area %	Relative Height %	Amount n.a.
1		10,517	0,329	2,068	0,51	0,25	n.a.
2		10,910	0,181	1,448	0,28	0,18	n.a.
3		11,273	0,473	6,134	0,73	0,75	n.a.
4		11,567	0,394	4,868	0,61	0,60	n.a.
5		12,233	0,343	2,671	0,53	0,33	n.a.
6		12,480	0,626	8,056	0,97	0,99	n.a.
7		12,640	61,420	781,478	95,15	96,02	n.a.
8		12,807	0,461	4,197	0,71	0,52	n.a.
9		12,987	0,053	0,784	0,08	0,10	n.a.
10		13,357	0,065	0,693	0,10	0,09	n.a.
11		14,413	0,208	1,476	0,32	0,18	n.a.
**Total:**			**64,554**	**813,872**	**100,00**	**100,00**	

HPLC chromatogram of SLW132 (**21m**).

REFERENCES

(1) Zlotnik, A.; Yoshie, O.; Nomiyama, H. The chemokine and chemokine receptor superfamilies and their molecular evolution. *Genome Biol.* **2006**, *7* (12), 243. DOI: 10.1186/gb-2006-7-12-243.

(2) Zheng, Y.; Qin, L.; Zacarías, N. V. O.; Vries, H. de; Han, G. W.; Gustavsson, M.; Dabros, M.; Zhao, C.; Cherney, R. J.; Carter, P.; Stamos, D.; Abagyan, R.; Cherezov, V.; Stevens, R. C.; IJzerman, A. P.; Heitman, L. H.; Tebben, A.; Kufareva, I.; Handel, T. M. Structure of CC chemokine receptor 2 with orthosteric and allosteric antagonists. *Nature* **2016**, *540* (7633), 458–461. DOI: 10.1038/nature20605.

(3) Toy, L.; Huber, M. E.; Schmidt, M. F.; Weikert, D.; Schiedel, M. Fluorescent Ligands Targeting the Intracellular Allosteric Binding Site of the Chemokine Receptor CCR2. *ACS Chem. Biol.* **2022**, *17* (8), 2142–2152. DOI: 10.1021/acschembio.2c00263.

(4) Huber, M. E.; Toy, L.; Schmidt, M. F.; Vogt, H.; Budzinski, J.; Wiefhoff, M. F. J.; Merten, N.; Kostenis, E.; Weikert, D.; Schiedel, M. A Chemical Biology Toolbox Targeting the Intracellular Binding Site of CCR9: Fluorescent Ligands, New Drug Leads and PROTACs. *Angew. Chem. Int. Ed.* **2022**, *61* (12), e202116782. DOI: 10.1002/anie.202116782.
